# Supplementary material for: Targeting SWI/SNF ATPases reduces neuroblastoma cell plasticity
Source: EMBO J. 2024 Aug 22;43(20):4522–41. doi: 10.1038/s44318-024-00206-1 (PMC11480351; doi:10.1038/s44318-024-00206-1)
Supplement: Supplementary file 1 — Appendix [file 44318_2024_206_MOESM1_ESM.pdf]

Appendix

Targeting SWI/SNF ATPases reduces neuroblastoma cell plasticity

Man Xu[1], Jason J. Hong[1], Xiyuan Zhang[1], Ming Sun[1], Xingyu Liu[1], Jeeyoun Kang[1], Hannah Stack[1], Wendy Fang[1], Haiyan Lei[1], Xavier Lacoste[1], Reona Okada[1], Raina Jung[1], Rosa Nguyen[1], Jack F. Shern[1], Carol J. Thiele[1*] and Zhihui Liu[1*]

[1]Pediatric Oncology Branch, Center for Cancer Research, National Cancer Institute, Bethesda, MD, USA

[*]Correspondence: liuzhihu@mail.nih.gov; thielec@mail.nih.gov.

Table of content

Appendix Figure S1	Page 2-6
Appendix Figure S2	Page 7-8
Appendix Figure S3	Page 9-10
Appendix Figure S4	Page 11-12
Appendix Figure S5	Page 13-16
Appendix Figure S6	Page 17-19

Fig. S1

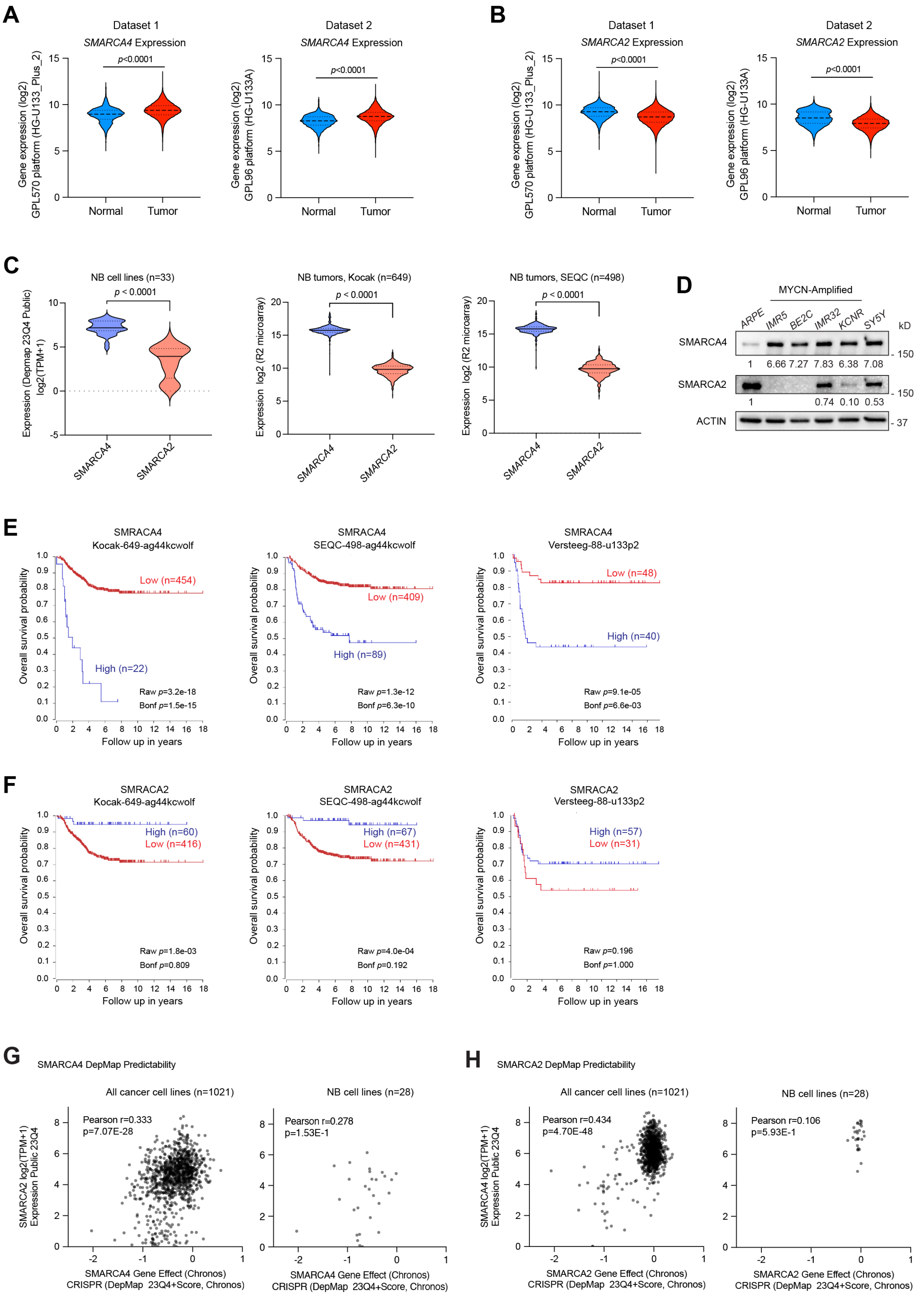

A
Dataset 1
SMARCA4 Expression
Dataset 2
SMARCA4 Expression
p<0.0001
Gene expression (log2) GPL570 platform (HG-U133_Plus_2)
Gene expression (log2) GPL96 platform (HG-U133A)
Normal
Tumor
B
Dataset 1
SMARCA2 Expression
Dataset 2
SMARCA2 Expression
C
NB cell lines (n=33)
NB tumors, Kocak (n=649)
NB tumors, SEQC (n=498)
p < 0.0001
Expression (Depmap 23Q4 Public) log2(TPM+1)
Expression log2 (R2 microarray)
SMARCA4
SMARCA2
D
MYCN-Amplified
ARPE
IMR5
BE2C
IMR32
KCNR
SY5Y
kD
SMARCA4
1 6.66 7.27 7.83 6.38 7.08
- 150
SMARCA2
1 0.74 0.10 0.53
- 150
ACTIN
- 37
E
SMRACA4
Kocak-649-ag44kcwolf
SEQC-498-ag44kcwolf
Versteeg-88-u133p2
Overall survival probability
Follow up in years
Low (n=454)
High (n=22)
Raw p=3.2e-18
Bonf p=1.5e-15
Low (n=409)
High (n=89)
Raw p=1.3e-12
Bonf p=6.3e-10
Low (n=48)
High (n=40)
Raw p=9.1e-05
Bonf p=6.6e-03
F
SMRACA2
Kocak-649-ag44kcwolf
SEQC-498-ag44kcwolf
Versteeg-88-u133p2
High (n=60)
Low (n=416)
Raw p=1.8e-03
Bonf p=0.809
High (n=67)
Low (n=431)
Raw p=4.0e-04
Bonf p=0.192
High (n=57)
Low (n=31)
Raw p=0.196
Bonf p=1.000
G
SMARCA4 DepMap Predictability
All cancer cell lines (n=1021)
NB cell lines (n=28)
Pearson r=0.333
p=7.07E-28
Pearson r=0.278
p=1.53E-1
SMARCA2 log2(TPM+1) Expression Public 23Q4
SMARCA4 Gene Effect (Chronos) CRISPR (DepMap 23Q4+Score, Chronos)
H
SMARCA2 DepMap Predictability
All cancer cell lines (n=1021)
NB cell lines (n=28)
Pearson r=0.434
p=4.70E-48
Pearson r=0.106
p=5.93E-1
SMARCA4 log2(TPM+1) Expression Public 23Q4
SMARCA2 Gene Effect (Chronos) CRISPR (DepMap 23Q4+Score, Chronos)

Fig. S1 continued 1

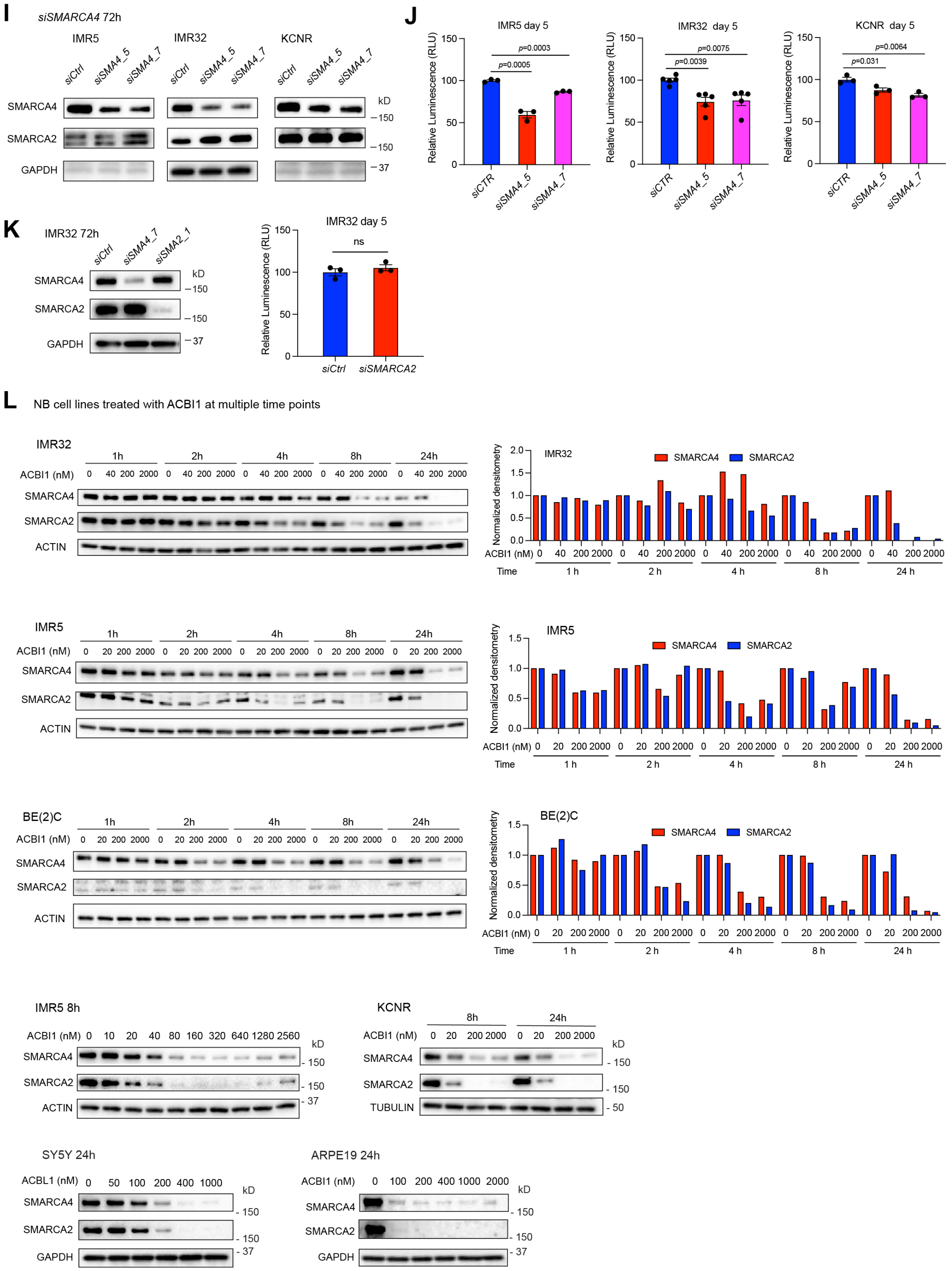

Fig. S1 continued 2

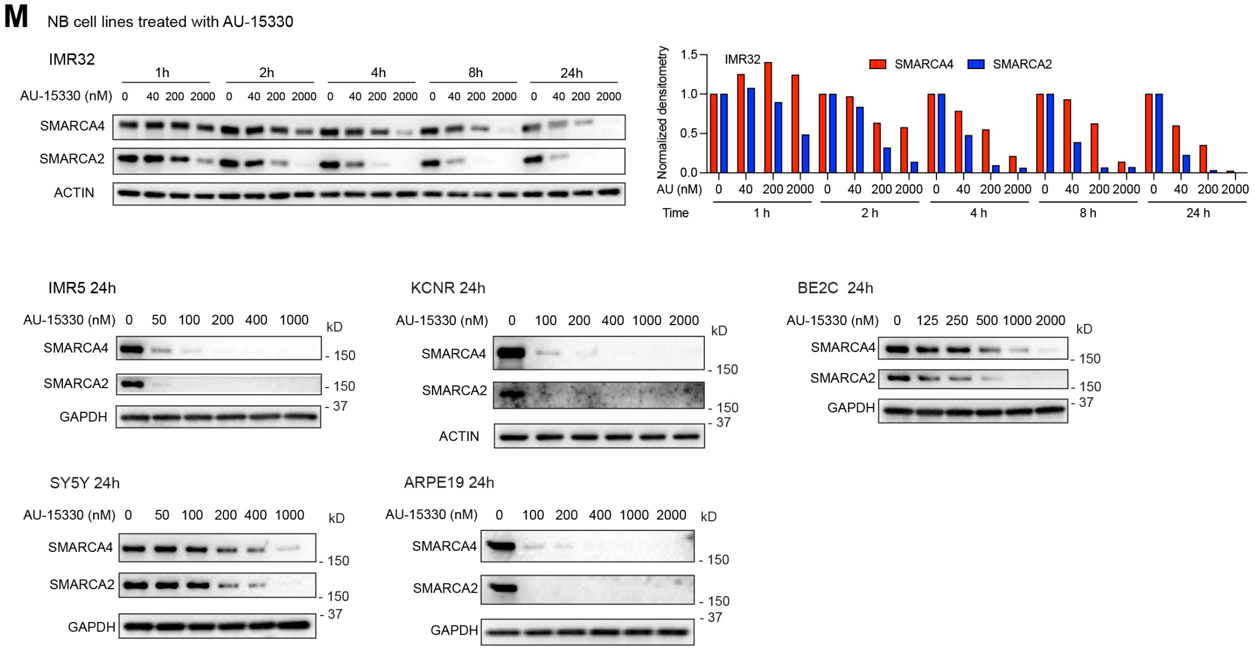

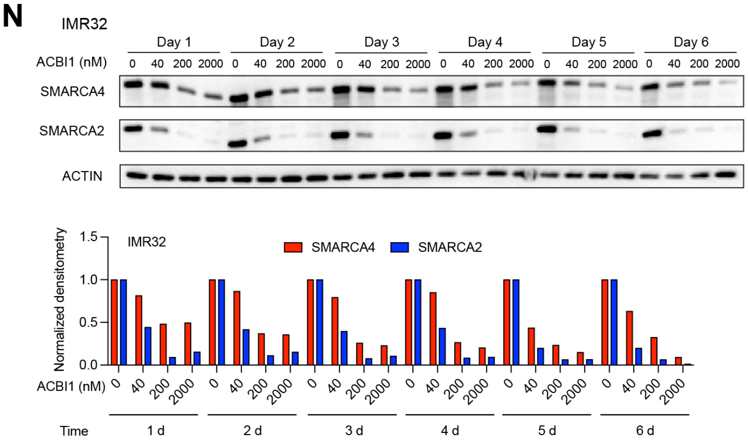

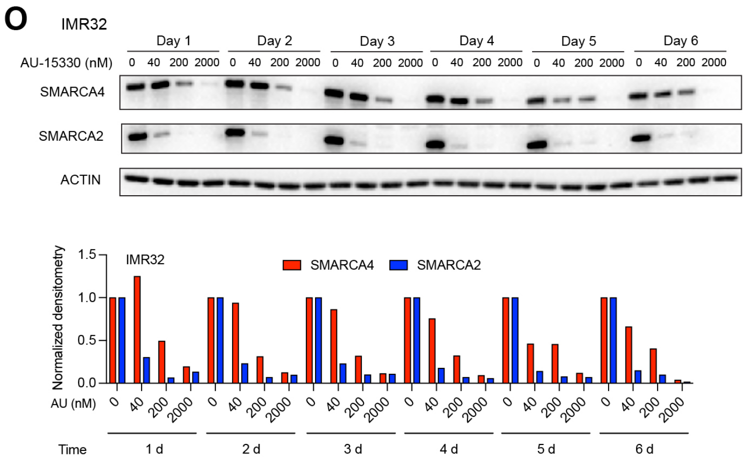

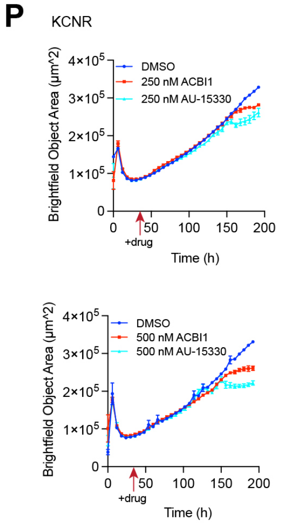

Fig. S1 continued 3

Q IMR5, day 4

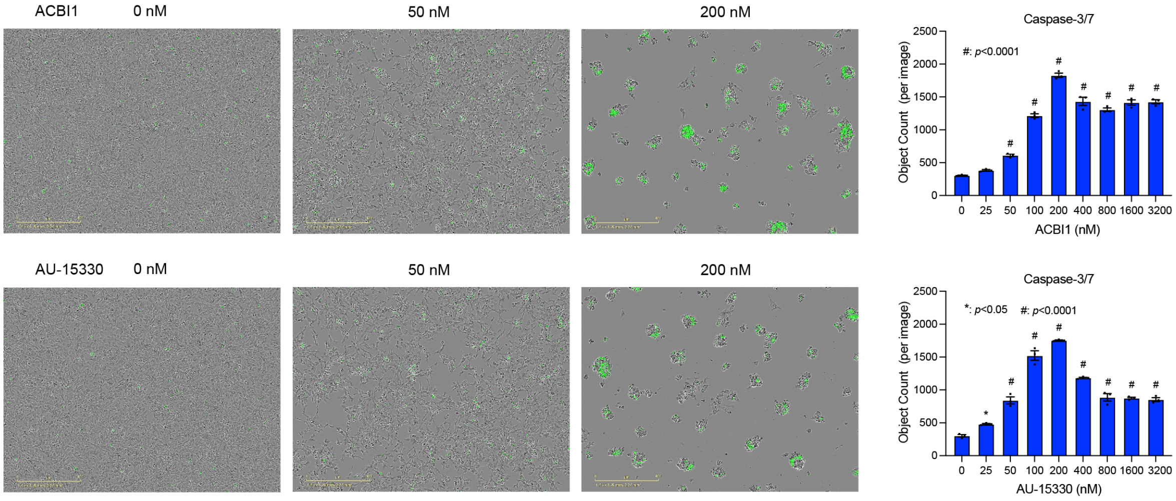

R IMR32, day 4

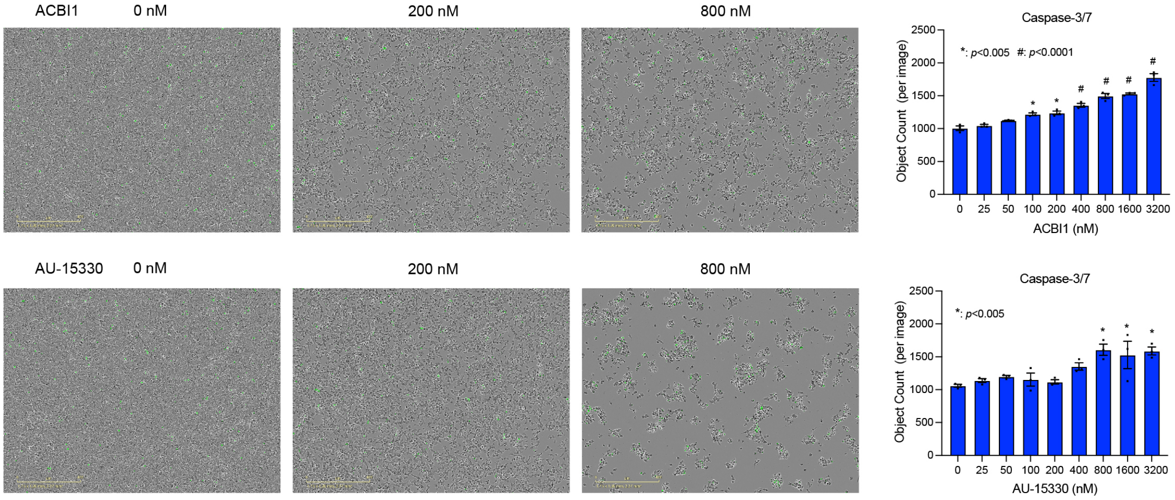

S BE(2)C, day 4

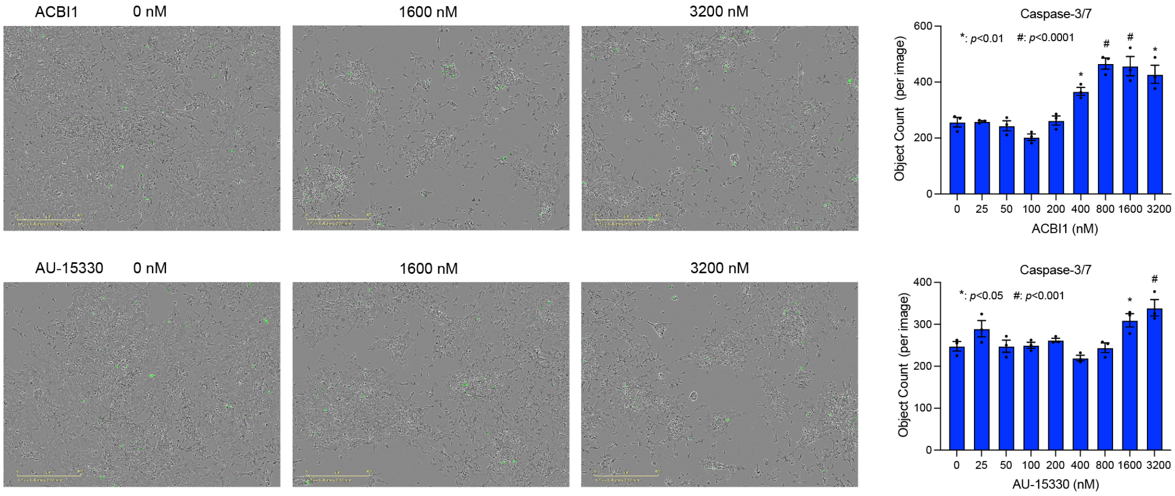

Appendix Figure S1. Targeting SWI/SNF ATPases inhibits NB growth (supplementary to Fig. 1). (**A**) Analysis of the GENT2 gene expression database (gent2.appex.kr) reveals significantly higher *SMARCA4* mRNA levels in all-cancers compared to all-normal tissues from two different datasets. (**B**) Analysis of the GENT2 gene expression database shows significantly lower *SMARCA2* mRNA levels in all-cancers compared to all-normal tissues from two different datasets. (**C**) Comparison of *SMARCA4* mRNA levels and *SMARCA2* mRNA levels in the 25 NB cell lines (queried from depmap.org), as well as in 649 (Kocak-GSE45547) and 498 (SEQC ag44kcwolf – GSE49710) patient NB samples (queried from r2.amc.nl). (**D**) Protein levels of SMARCA2 and SMARCA4 detected by western blot assay in NB cell lines and the ARPE-19 cell line. ACTIN is used as loading control. (**E,F**) The correlation between *SMARCA4* and *SMARCA2* mRNA levels and overall survival in three clinically annotated NB cohorts. *SMARCA4* and *SMARCA2* mRNA expression of 649 (Kocak-GSE45547), 498 (SEQC ag44kcwolf – GSE49710), and 88 (Versteeg-GSE16476) patient NB samples were analyzed using the R2 genomics analysis and visualization platform (r2.amc.nl). (**G**) The expression levels of *SMARCA2* are correlated with the CRISPR knockout effect of *SMARCA4* whether analyzed all the cancer cell lines (with a moderate Pearson correlation coefficient r value of 0.333, left panel) or only analyzed NB cell lines (with a weak Pearson correlation coefficient r value of 0.278, right panel). Note: Pearson correlation coefficient (r) value that is greater than 0.5 represents a strong positive, between 0.3 and 0.5 represents a moderate positive, and between 0 and 0.3 represents a weak positive correlation. (**H**) The expression of *SMARCA4* is correlated with the CRISPR knockout effect of *SMARCA2* when analyzing all the cancer cell lines (Pearson r value = 0.434, left panel), but the expression of *SMARCA4* is barely correlated with the knockout effect of *SMARCA2* if only analyzed NB cell lines (Pearson r=0.106, right panel). (**I**) Transient transfection of different *SMARCA4* siRNAs (*siSMA4_5* and *siSMA4_7*) in NB cells results in decreased SMARCA4 protein levels compared to control *siRNA* transfected cells (*siCtrl*). (**J**) CellTiter-Glo assay shows the effect of genetically silencing *SMARCA4* on cell proliferation. (**K**) Genetic silencing of *SMARCA2* in IMR32 cells results in a decrease of SMARCA2 protein levels but does not affect cell proliferation. (**L,M**) ACBI1 or AU-15330 treatment decreases SMARCA2 and SMARCA4 protein levels in all tested cell lines, as detected by western blot. (**N,O**) Western blot analysis reveals that ACBI1 or AU-15330 treatment of IMR32 cells leads to SMARCA2/4degradation without any subsequent recovery of protein levels, even at day 5. (**P**) Impact of different doses of ACBI1 or AU-15330 treatment on KCNR cell single spheroid growth in liquid 3D culture. (**Q-S**) Impact of different doses of ACBI1 or AU-15330 treatment on NB cell apoptosis through using Incucyte Caspase-3/7 Green Apoptosis Assay. Left panel, representative images of NB cells treated with ACBI1 or AU-15330 for 4 days in the presence of Caspase-3/7 green dye. Right panel, apoptotic NB cell counts were acquired through the Incucyte Live-Cell Analysis System when cells were treated with increasing concentrations of ACBI1 or AU-15330 for 4 days. Note: In panel (**A-C**), data are presented as violin plots, where the middle solid lines indicate medians, and the dashed lines represent the 25th and 75th percentiles. Statistical differences were calculated using a two-sided unpaired Student's *t*-test. In panel (**Q-S**), the data are represented as mean ± SEM. Statistical differences were calculated using ordinary one-way ANOVA.

Fig. S2

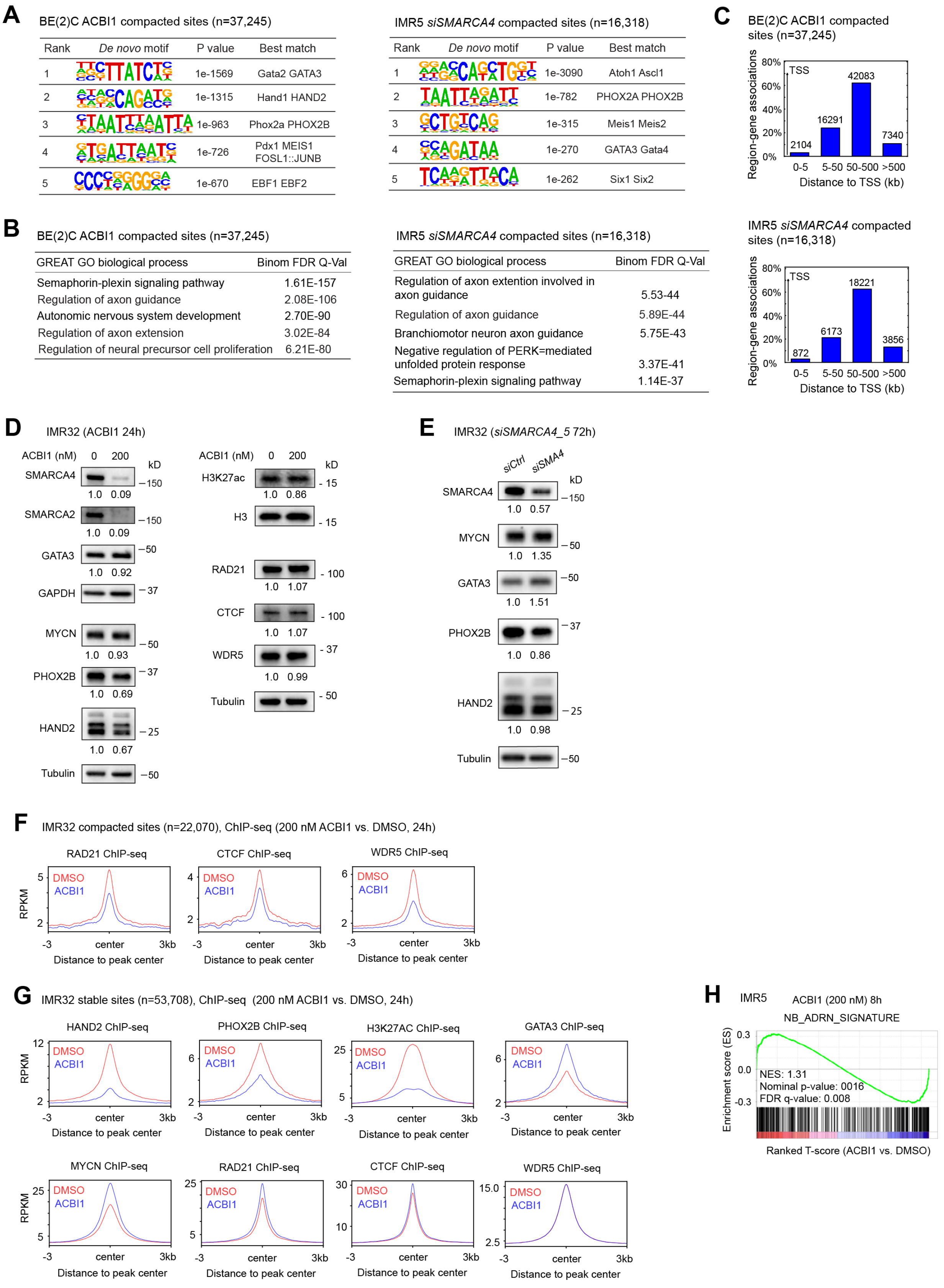
A
BE(2)C ACBI1 compacted sites (n=37,245)
Rank | De novo motif | P value | Best match
1 | 1e-1569 | Gata2 GATA3
2 | 1e-1315 | Hand1 HAND2
3 | 1e-963 | Phox2a PHOX2B
4 | 1e-726 | Pdx1 MEIS1 FOSL1::JUNB
5 | 1e-670 | EBF1 EBF2
IMR5 siSMARCA4 compacted sites (n=16,318)
Rank | De novo motif | P value | Best match
1 | 1e-3090 | Atoh1 Ascl1
2 | 1e-782 | PHOX2A PHOX2B
3 | 1e-315 | Meis1 Meis2
4 | 1e-270 | GATA3 Gata4
5 | 1e-262 | Six1 Six2
B
BE(2)C ACBI1 compacted sites (n=37,245)
GREAT GO biological process | Binom FDR Q-Val
Semaphorin-plexin signaling pathway | 1.61E-157
Regulation of axon guidance | 2.08E-106
Autonomic nervous system development | 2.70E-90
Regulation of axon extension | 3.02E-84
Regulation of neural precursor cell proliferation | 6.21E-80
IMR5 siSMARCA4 compacted sites (n=16,318)
GREAT GO biological process | Binom FDR Q-Val
Regulation of axon extention involved in axon guidance | 5.53-44
Regulation of axon guidance | 5.89E-44
Branchiomotor neuron axon guidance | 5.75E-43
Negative regulation of PERK=mediated unfolded protein response | 3.37E-41
Semaphorin-plexin signaling pathway | 1.14E-37
C
BE(2)C ACBI1 compacted sites (n=37,245)
Region-gene associations
TSS
2104 16291 42083 7340
0-5 5-50 50-500 >500
Distance to TSS (kb)
IMR5 siSMARCA4 compacted sites (n=16,318)
872 6173 18221 3856
D
IMR32 (ACBI1 24h)
ACBI1 (nM) 0 200
SMARCA4 1.0 0.09
SMARCA2 1.0 0.09
GATA3 1.0 0.92
GAPDH
MYCN 1.0 0.93
PHOX2B 1.0 0.69
HAND2 1.0 0.67
Tubulin
H3K27ac 1.0 0.86
H3
RAD21 1.0 1.07
CTCF 1.0 1.07
WDR5 1.0 0.99
Tubulin
E
IMR32 (siSMARCA4_5 72h)
siCtrl siSMA4
SMARCA4 1.0 0.57
MYCN 1.0 1.35
GATA3 1.0 1.51
PHOX2B 1.0 0.86
HAND2 1.0 0.98
Tubulin
F
IMR32 compacted sites (n=22,070), ChIP-seq (200 nM ACBI1 vs. DMSO, 24h)
RAD21 ChIP-seq
CTCF ChIP-seq
WDR5 ChIP-seq
DMSO
ACBI1
RPKM
Distance to peak center
G
IMR32 stable sites (n=53,708), ChIP-seq (200 nM ACBI1 vs. DMSO, 24h)
HAND2 ChIP-seq
PHOX2B ChIP-seq
H3K27AC ChIP-seq
GATA3 ChIP-seq
MYCN ChIP-seq
RAD21 ChIP-seq
CTCF ChIP-seq
WDR5 ChIP-seq
H
IMR5
ACBI1 (200 nM) 8h
NB_ADRN_SIGNATURE
Enrichment score (ES)
NES: 1.31
Nominal p-value: 0016
FDR q-value: 0.008
Ranked T-score (ACBI1 vs. DMSO)

Appendix Figure S2. Depletion of SWI/SNF ATPases decreases chromatin accessibility and disrupts core TFs from binding to DNA (supplementary to Fig. 2). (A) HOMER *de novo* motif scan of sites with compacted chromatin after ACBI1 treatment of BE(2)C cells or genetic silencing of *SMARCA4* in IMR5 cells reveal enrichment of non-canonical E-boxes and binding motifs of core TFs HAND2, PHOX2B, GATA3, and MEIS2. (**B)** GREAT GO analysis shows that genes associated with reduced chromatin accessibility in BE(2)C and IMR5 are enriched in the regulation of axon guidance and semaphorin-plexin signaling pathway**. (C)** ATAC-seq peak distribution analysis in BE(2)C and IMR5 indicates that compacted sites are enriched in the distal regulatory regions. (**D,E**) Western blot analysis of the protein levels of indicated proteins after ACBI1 treatment or genetic silencing of *SMARCA4*. (**F**) Metagene plots illustrate the decreased average ChIP-seq signals in RAD21, CTCF, and WDR5 at the sites with reduced chromatin accessibility after ACBI1 treatment of IMR32 cells for 24 h. (**G**) Metagene plots illustrate the changes in the average ChIP-seq signals in core TFs, RAD21, CTCF, and WDR5 at the sites with unchanged chromatin accessibility after ACBI1 treatment of IMR32 cells for 24 h. (**H**) Gene set enrichment analysis (GSEA) of RNAs-seq data shows negative enrichment of genes highly expressed in ADRN-type of NB following treatment with ACBI1 for 8 h in IMR5 cells.

Fig. S3

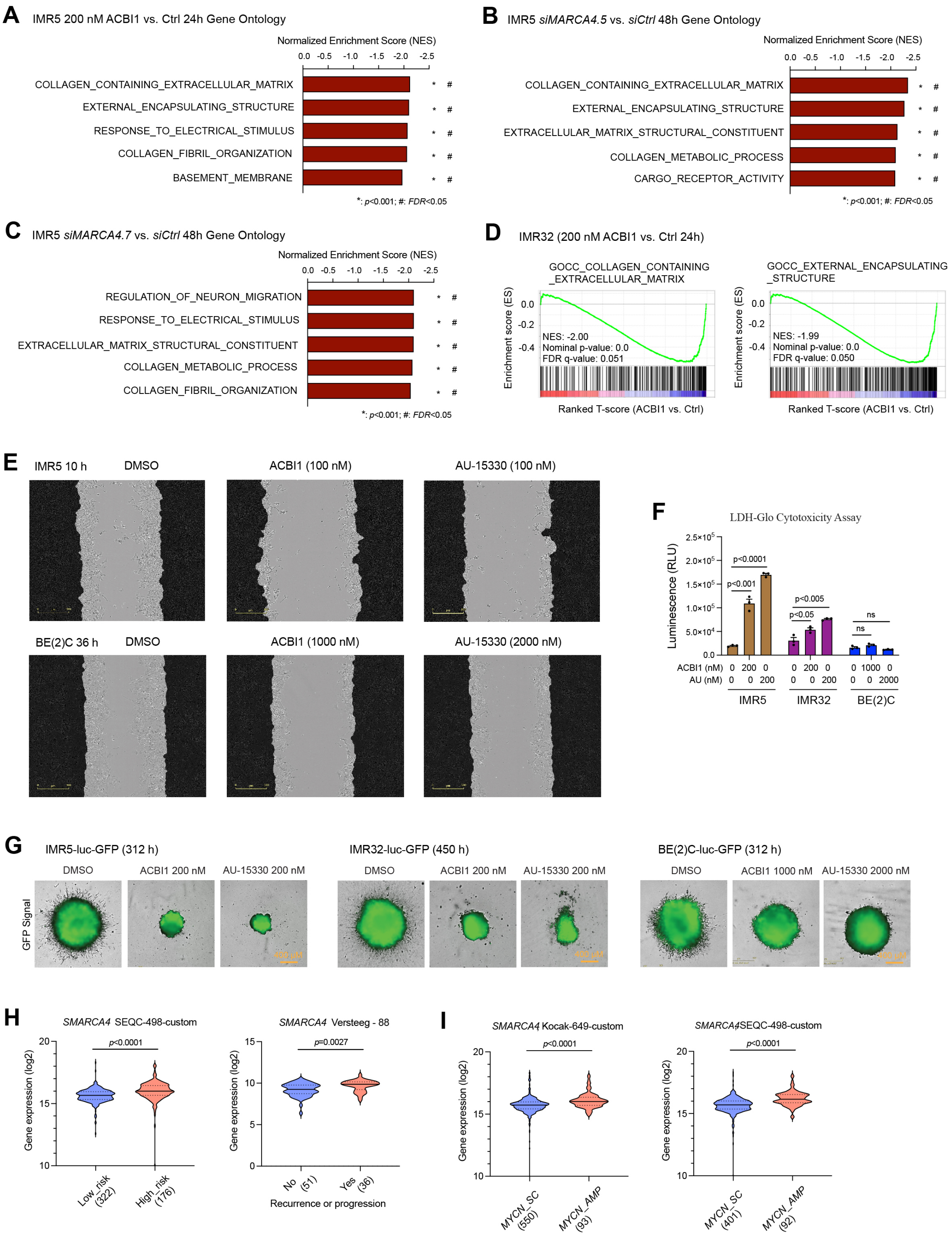

A
IMR5 200 nM ACBI1 vs. Ctrl 24h Gene Ontology
Normalized Enrichment Score (NES)
COLLAGEN_CONTAINING_EXTRACELLULAR_MATRIX
EXTERNAL_ENCAPSULATING_STRUCTURE
RESPONSE_TO_ELECTRICAL_STIMULUS
COLLAGEN_FIBRIL_ORGANIZATION
BASEMENT_MEMBRANE
*: p<0.001; #: FDR<0.05
B
IMR5 siMARCA4.5 vs. siCtrl 48h Gene Ontology
Normalized Enrichment Score (NES)
COLLAGEN_CONTAINING_EXTRACELLULAR_MATRIX
EXTERNAL_ENCAPSULATING_STRUCTURE
EXTRACELLULAR_MATRIX_STRUCTURAL_CONSTITUENT
COLLAGEN_METABOLIC_PROCESS
CARGO_RECEPTOR_ACTIVITY
*: p<0.001; #: FDR<0.05
C
IMR5 siMARCA4.7 vs. siCtrl 48h Gene Ontology
Normalized Enrichment Score (NES)
REGULATION_OF_NEURON_MIGRATION
RESPONSE_TO_ELECTRICAL_STIMULUS
EXTRACELLULAR_MATRIX_STRUCTURAL_CONSTITUENT
COLLAGEN_METABOLIC_PROCESS
COLLAGEN_FIBRIL_ORGANIZATION
*: p<0.001; #: FDR<0.05
D
IMR32 (200 nM ACBI1 vs. Ctrl 24h)
GOCC_COLLAGEN_CONTAINING_EXTRACELLULAR_MATRIX
NES: -2.00
Nominal p-value: 0.0
FDR q-value: 0.051
GOCC_EXTERNAL_ENCAPSULATING_STRUCTURE
NES: -1.99
Nominal p-value: 0.0
FDR q-value: 0.050
Enrichment score (ES)
Ranked T-score (ACBI1 vs. Ctrl)
E
IMR5 10 h
DMSO
ACBI1 (100 nM)
AU-15330 (100 nM)
BE(2)C 36 h
DMSO
ACBI1 (1000 nM)
AU-15330 (2000 nM)
F
LDH-Glo Cytotoxicity Assay
Luminescence (RLU)
p<0.0001
p<0.001
p<0.005
p<0.05
ns
ns
ACBI1 (nM)
AU (nM)
IMR5
IMR32
BE(2)C
G
IMR5-luc-GFP (312 h)
IMR32-luc-GFP (450 h)
BE(2)C-luc-GFP (312 h)
DMSO
ACBI1 200 nM
AU-15330 200 nM
ACBI1 1000 nM
AU-15330 2000 nM
GFP Signal
400 uM
H
SMARCA4 SEQC-498-custom
p<0.0001
Gene expression (log2)
Low_risk (322)
High_risk (176)
SMARCA4 Versteeg - 88
p=0.0027
No (51)
Yes (36)
Recurrence or progression
I
SMARCA4 Kocak-649-custom
p<0.0001
MYCN_SC (550)
MYCN_AMP (93)
SMARCA4 SEQC-498-custom
p<0.0001
MYCN_SC (401)
MYCN_AMP (92)

Appendix Figure S3. SWI/SNF ATPases drive invasive transcriptional program and depletion of SWI/SNF ATPases suppresses NB invasion (supplementary to Fig. 3). (**A-C**) GSEA gene ontologies (GO) analysis reveals a negative enrichment of gene sets related to collagen-containing extracellular matrix and collagen fibril organization after ACBI1 treatment or genetically silencing of *SMARCA4* in IMR5 cells. (**D**) GSEA GO analysis reveals a significantly negative enrichment of genes encoding collagen-containing extracellular matrix and external encapsulating structure proteins after ACBI1 treatment in IMR32 cells. (**E**) IncuCyte scratch wound healing assay shows that the treatment of IMR5 and BE(2)C with ACBI1 or AU-15330 reduces relative would density, as depicted by cell images. **(F)** LDH-Glo Cytotoxity Assays show that treatment with ACBI1 and AU-15330 significantly increased cell death in IMR5 and IMR32 cells, but not in BE(2)C cells. **(G)** Images from IncuCyte single spheroid matrigel invasion assay show similar GFP signal intensity in IMR5-luc-GFP, IMR32-luc-GFP and BE(2)C-luc-GFP cells with or without ACBI1, or AU-15330 treatment. (**H**) *SMARCA4* mRNA levels are higher in high-risk NB patients or patients with recurrence compared to the rest patients. *SMARCA4* mRNA expression was analyzed in 498 (SEQC ag44kcwolf – GSE49710) and 88 (Versteeg-GSE16476) patient NB (data queried from r2.amc.nl). (**I**) *SMARCA4* mRNA levels are higher in *MYCN* amplified (*MYCN_AMP*) NB patients compared to *MYCN* single copy (*MYCN_SC*) NB patients. *SMARCA4* mRNA levels in 649 (Kocak-GSE45547) and 498 (SEQC ag44kcwolf – GSE49710) patient NB samples were analyzed using the data queried from R2 genomics analysis and visualization platform (r2.amc.nl). Note: In panel (**H,I**), data are presented as violin plots, where the middle solid lines indicate medians, and the dash lines represent the 25th and 75th percentiles. Statistical differences were calculated using a two-sided unpaired Student's *t*-test.

Fig. S4

A

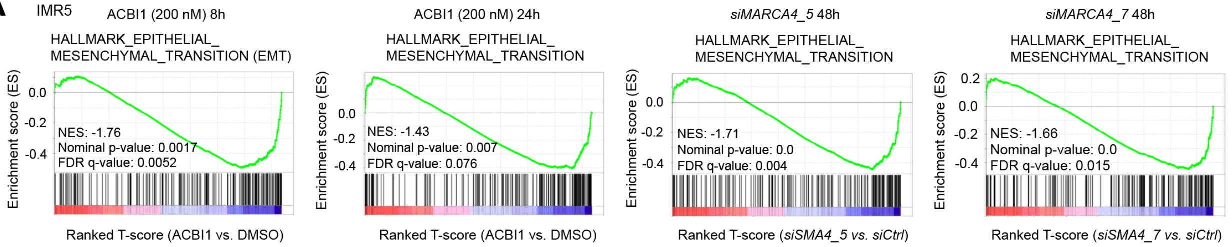

B

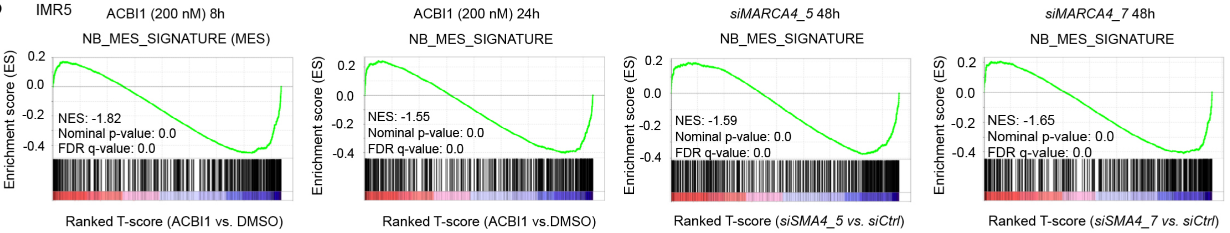

C

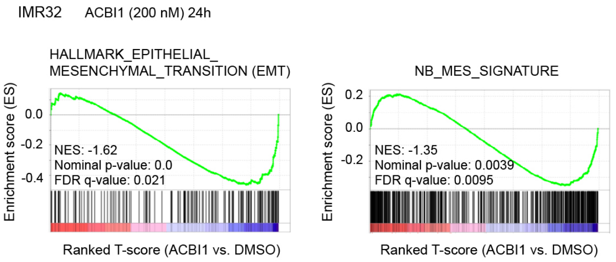

D IMR32

ADRN genes associated ATAC-seq peaks

Rank	*De novo* motif	P value	Best match
1	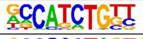	1e-254	NeuroD1 HAND2
2	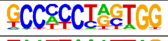	1e-140	BORIS CTCF
3	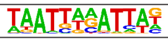	1e-96	Phox2a Phox2b
4	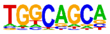	1e-64	HIC1 MEIS2
5	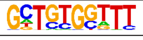	1e-49	RUNX1

MES genes associated ATAC-seq peaks

Rank	*De novo* motif	P value	Best match
1	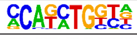	1e-243	BHLHA15 NeuroD1
2	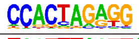	1e-115	CTCF BORIS
3	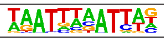	1e-86	Phox2a Phox2b
4	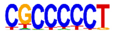	1e-67	CTCFL
5	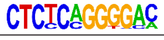	1e-52	EBF

E KELLY

ADRN genes associated ATAC-seq peaks

Rank	*De novo* motif	P value	Best match
1	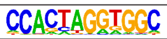	1e-93	CTCF BORIS
2	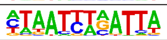	1e-53	Phox2a PHOX2B
3	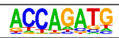	1e-51	TWIST1 HAND2
4	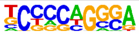	1e-49	EBF
5	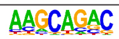	1e-41	Smad4

MES genes associated ATAC-seq peaks

Rank	*De novo* motif	P value	Best match
1	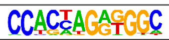	1e-112	BORIS CTCF
2	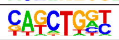	1e-47	Atoh1 Ascl1
3	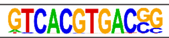	1e-44	BHLHE41 E-box
4	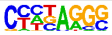	1e-43	AP-2alpha
5	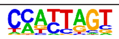	1e-42	Isl1

Fig. S4 continued

F IMR32

ADRN genes associated ATAC-seq peaks

GREAT GO biological process	Binom FDR Q-Val
Neuron development	0
Neuron differentiation	4.99E-270
Noradrenergic neuron differentiation	5.06E-228
Axonogenesis	6.74E-217
Axon development	1.21E-208
Noradrenergic neuron development	1.73E-203

MES genes associated ATAC-seq peaks

GREAT GO biological process	Binom FDR Q-Val
Extracellular matrix organization	8.45E-192
Extracellular structure organization	1.03E-191
IRE1-mediated unfolded protein response	8.09E-145
Substrate adhesion-dependent cell spreading	8.00E-136
Heart development	2.38E-122
Regulation of cell migration	2.60E-119

G KELLY

ADRN genes associated ATAC-seq peaks

GREAT GO biological process	Binom FDR Q-Val
Neuron development	3.12E-214
Regulation of neurogenesis	8.12E-153
Cytoskeleton organization	9.62E-119
Neuron projection development	1.59E-115
Positive regulation of neurogenesis	1.05E-113
Regulation of cell projection organization	4.84E-111

MES genes associated ATAC-seq peaks

GREAT GO biological process	Binom FDR Q-Val
Extracellular matrix organization	1.53E-313
Extracellular structure organization	2.90E-313
Heart development	6.97E-271
Regulation of cell migration	3.54E-255
Regulation of cell motility	1.52E-239
Regulation of locomotion	2.13E-232

H

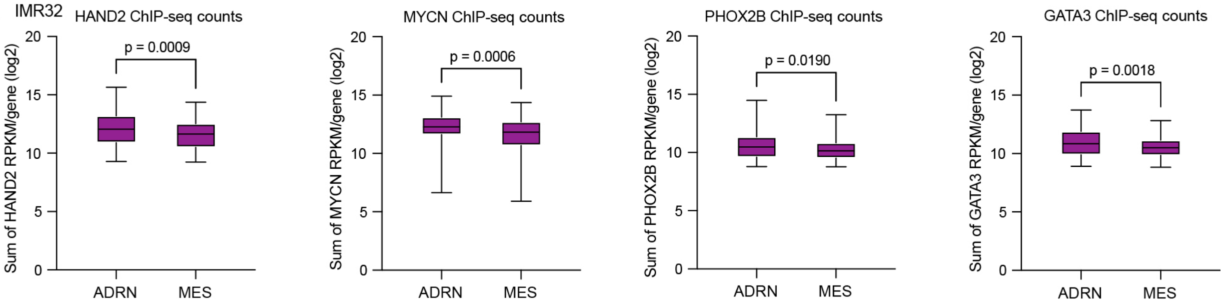

Appendix Figure S4. SWI/SNF ATPases maintain a permissive chromatin state for MES genes in ADRN-type NB (supplementary to Fig. 4). (**A,B**) GSEA reveals a significantly negative enrichment of EMT and MES signature genes after ACBI1 treatment or genetically silencing of *SMARCA4* in IMR5 cells. **(C)** GSEA reveals a significantly negative enrichment of EMT and MES signature genes after ACBI1 treatment in IMR32 cells. **(D,E)** HOMER motif scan analysis of ATAC-seq peaks associated with ADRN genes and MES genes reveals the enrichment of core adrenergic TFs binding motifs in IMR32 and KELLY cells. (**F,G**) GREAT GO analysis shows that ATAC-seq peaks associated with ADRN genes are enriched in neuron development, while peaks associated with MES genes are enriched in extracellular matrix organization in both IMR32 cells and KELLY cells. (**H**) ChIP-seq peaks of HAND2, MYCN, PHOX2B, and GATA3 are observed on both ADRN genes and MES genes, although the ChIP-seq signal intensities on MES genes are lower than on ADRN genes. Note: In panel (**H**), the data represent mean ± SEM, and statistical differences were calculated using a two-sided unpaired Student's *t*-test.

Fig. S5

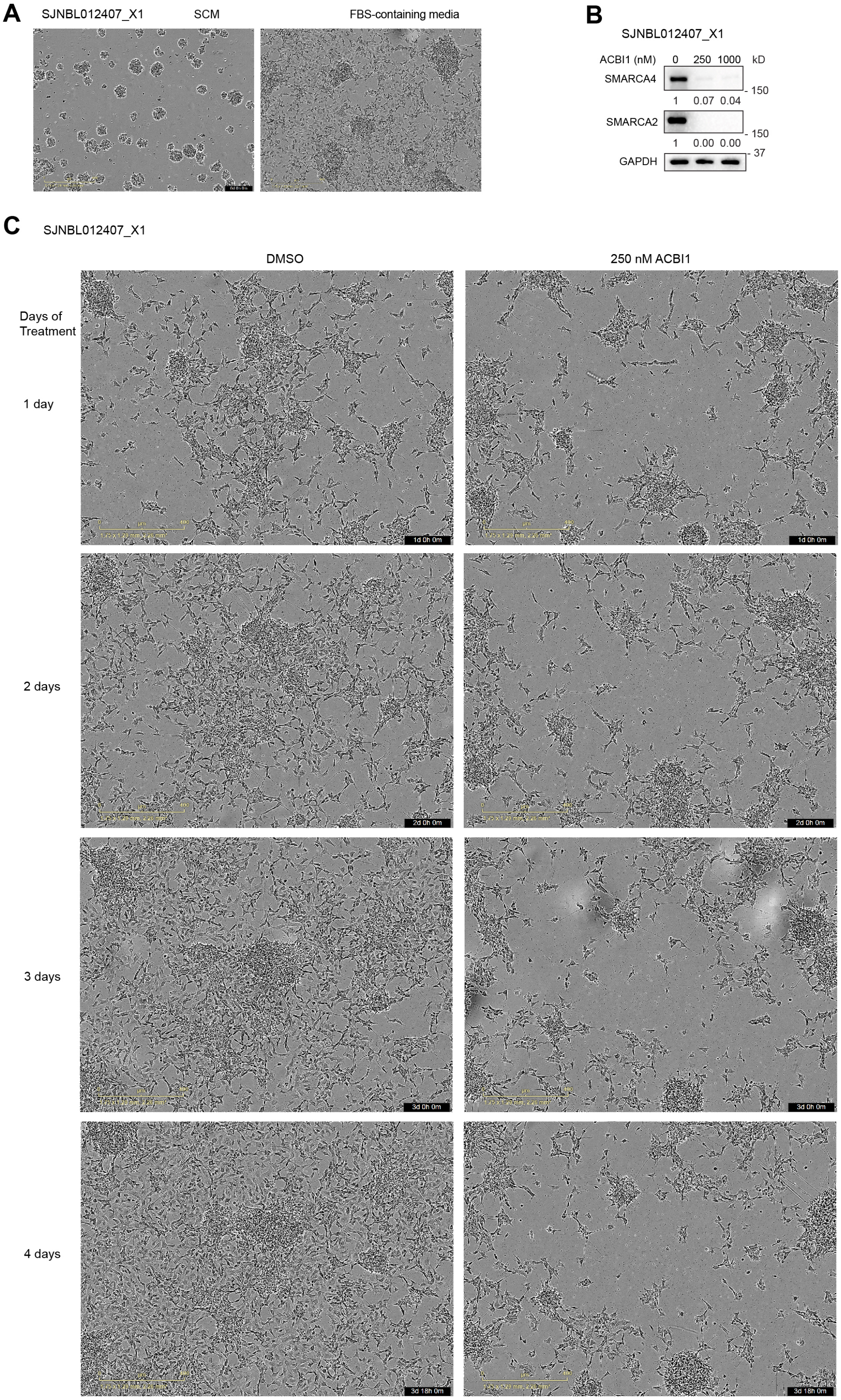

A
SJNBL012407_X1
SCM
FBS-containing media
B
SJNBL012407_X1
ACBI1 (nM) 0 250 1000 kD
SMARCA4
150
1 0.07 0.04
SMARCA2
150
1 0.00 0.00
37
GAPDH
C
SJNBL012407_X1
DMSO
250 nM ACBI1
Days of Treatment
1 day
2 days
3 days
4 days
1d 0h 0m
2d 0h 0m
3d 0h 0m
3d 18h 0m

Fig. S5 continued 1

D SJNBL012407_X1 in SCM

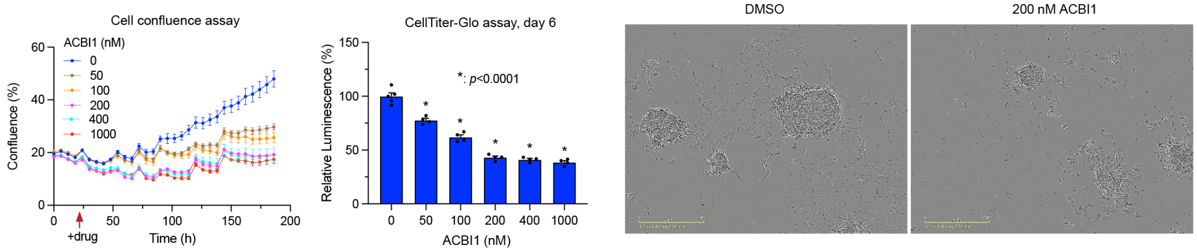

E SJNBL012407_X1, Incucyte Cytotox Green Dye staining

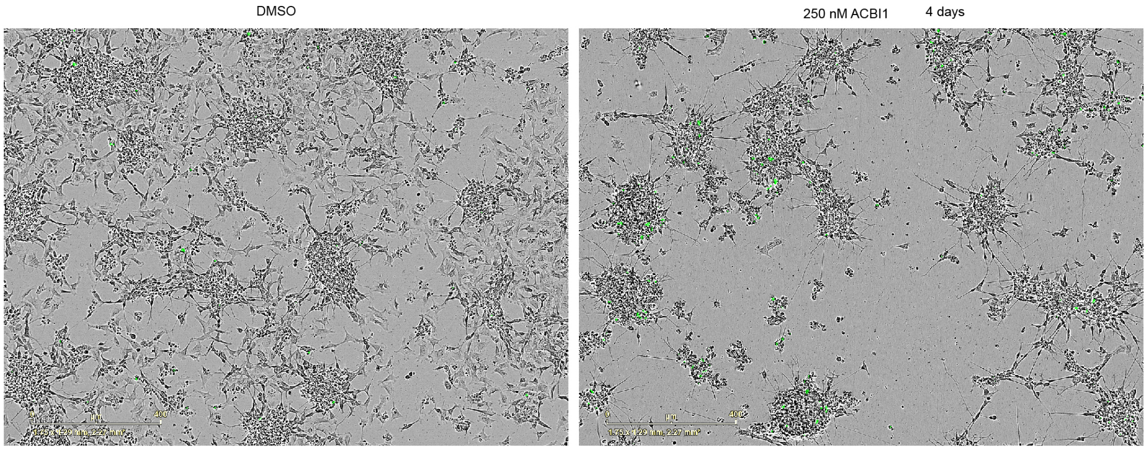

F

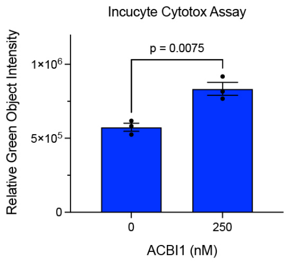

G SJNBL012407_X1, cultured in FBS-containing media for 3 days, then treated with DMSO or ACBI1

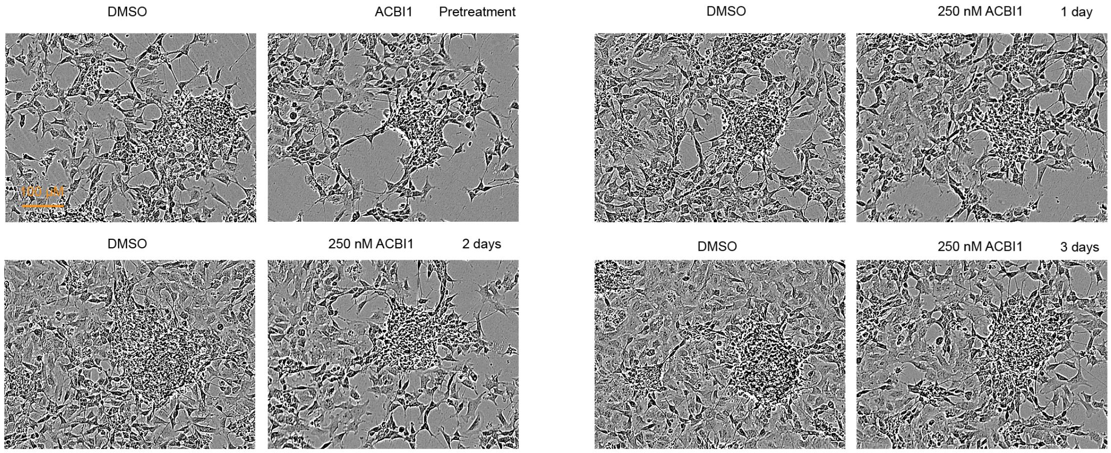

Fig. S5 continued 2

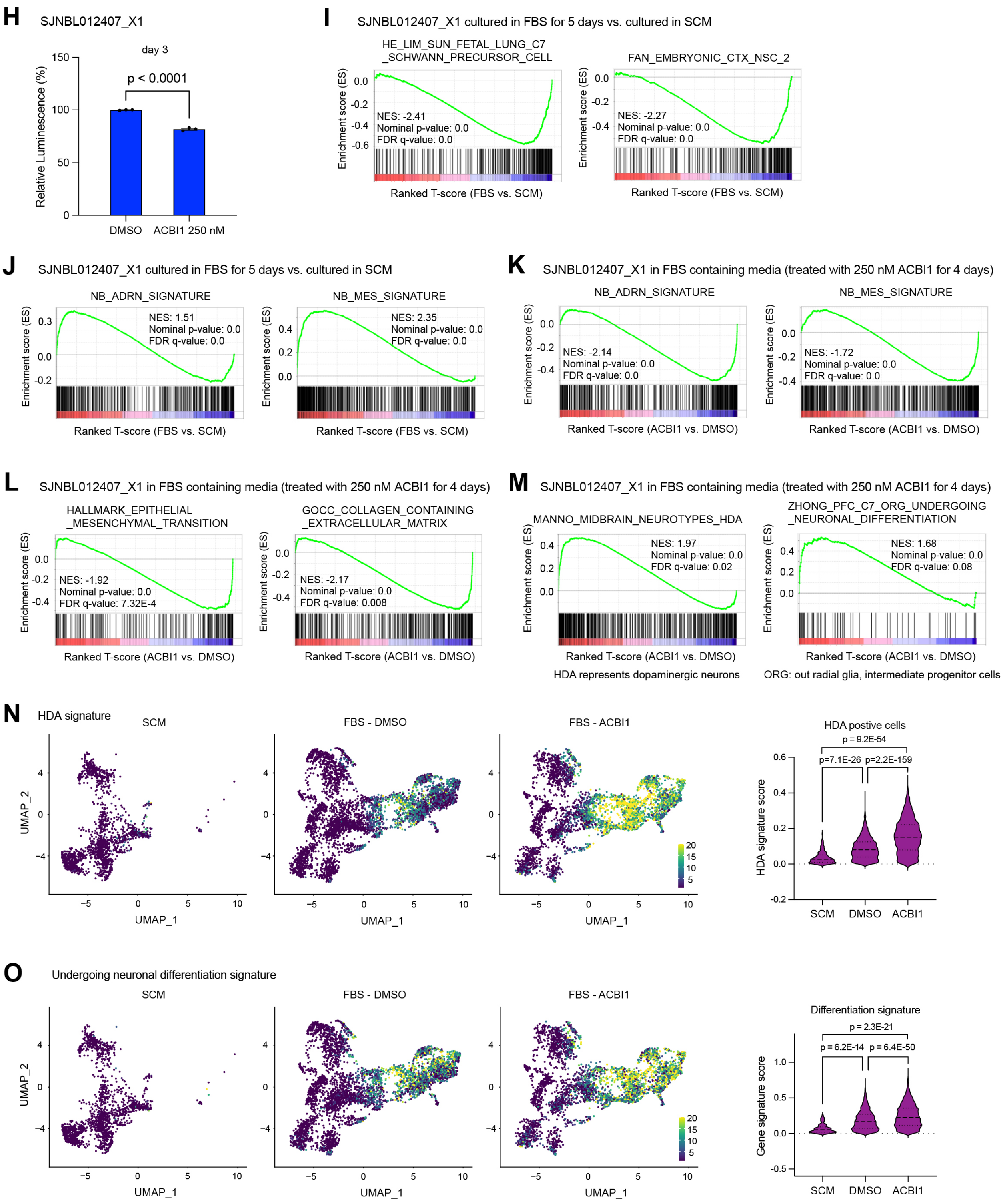

Appendix Figure S5. SWI/SNF ATPases depletion reduces NB cell heterogeneity and plasticity (supplementary to Fig. 5). (**A**) Images of SJNBL012407_X1 cells cultured in neural stem cell culture media (SCM) or complete media (RPMI 1640 containing 10% FBS). (**B**) Protein levels of SMARCA2 and

SMARCA4 detected by western blot assay in SJNBL012407_X1 cells after 24 h ACBI1 treatment. GAPDH is used as a loading control. **(C**) SJNBL012407_X1 was initially cultured in serum-free media (SCM) and then shifted to FBS-containing media overnight. Subsequently, it was treated with either DMSO or ACBI1. Time-lapse cell images illustrate the expansion of flattened, enlarged, MES-type monolayer cells in DMSO-treated SJNBL012407_X1 cells, and the reduced monolayer flat cells in the ACBI1-treated groups. **(D**) ACBI1 treatment of SJNBL012407_X1 cells cultured in SCM leads to a significant decrease in cell proliferation, as indicated by Incucyte cell confluence assay (left panel), CellTiter-Glo assay (middle panel), and the Incucyte captured cell images (right panel) (n=4; Error bars indicate SEM). Data are representative of two independent experiments. (**E**) Incucyte cytotoxicity assay indicates that ACBI1 treatment of SJNBL012407 induces cell death, as evidenced by the representative cell images stained with the Incucyte Cytotox Green Dye. (**F**) The significant increase in cell death following ACBI1 treatment is determined using the Incucyte Live-Cell Analysis System (n=3; Error bars indicate SEM). Data are representative of two independent experiments. **(G**) Time-lapse cell images show that initiating ACBI1 treatment after 3 days of culturing in FBS-containing media did not prevent the expansion of monolayer flat cells to the same extent as treating the cells with ACBI1 after overnight culturing in FBS-containing media. (**H**) CellTiter-Glo assay measures the impact of ACBI1 treatment on cell growth. In this experiment, ACBI1 treatment was initiated after 3 days of culturing in FBS-containing media for 3 days. The cell viabilities of DMSO-treated cells are set to 100%, and the bar graph was generated using GraphPad Prism software (n=3; Error bars indicate SEM). Data are representative of two independent experiments. (**I**) GSEA of the bulk RNA-seq shows the negative enrichment of SCP (Schwann cell precursor) genes and NSC (neural stem cell) genes after switching cell culture media from SCM to FBS-containing media for SJNBL012407_X1 cells. (**J**) GSEA of the bulk RNA-seq shows the positive enrichment of ADRN genes and MES genes after switching cell culture media from SCM to FBS-containing media. (**K,L**) GSEA of the bulk RNA-seq shows that ACBI1 treatment of PDX cells results in a negative enrichment of ADRN, MES, EMT, and collagen-containing extracellular matrix (CCEM) genes. (**M**) GSEA of the bulk RNA-seq shows that ACBI1 treatment results in a positive enrichment of HDA (immature dopaminergic neuron) signature genes and genes associated with cells undergoing neuronal differentiation. **(N,O**) UMAP plots show HDA and neuronal differentiation signature scores in SJNBL012407_X1 cells under indicated culture conditions. Left panels: gene signature score high cells are indicated by green and yellow dots; Right panel: statistical analysis of the average HDA or neuronal differentiation signature score per cell under different conditions. Data information: In (**D**) middle panel, the data are represented as mean ± SEM. Statistical differences were calculated using ordinary one-way ANOVA. In (**F,H**), statistical differences were calculated using a two-sided unpaired Student's *t*-test. In the right panels of **(N,O**), data are presented as violin plots, where the dashed lines indicate the median and the 25th and 75th percentiles. Statistical differences were calculated using a two-sided unpaired Student's *t*-test.

Fig. S6

A SJNBL012407_X1

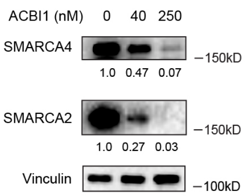

B SJNBL012407_X1

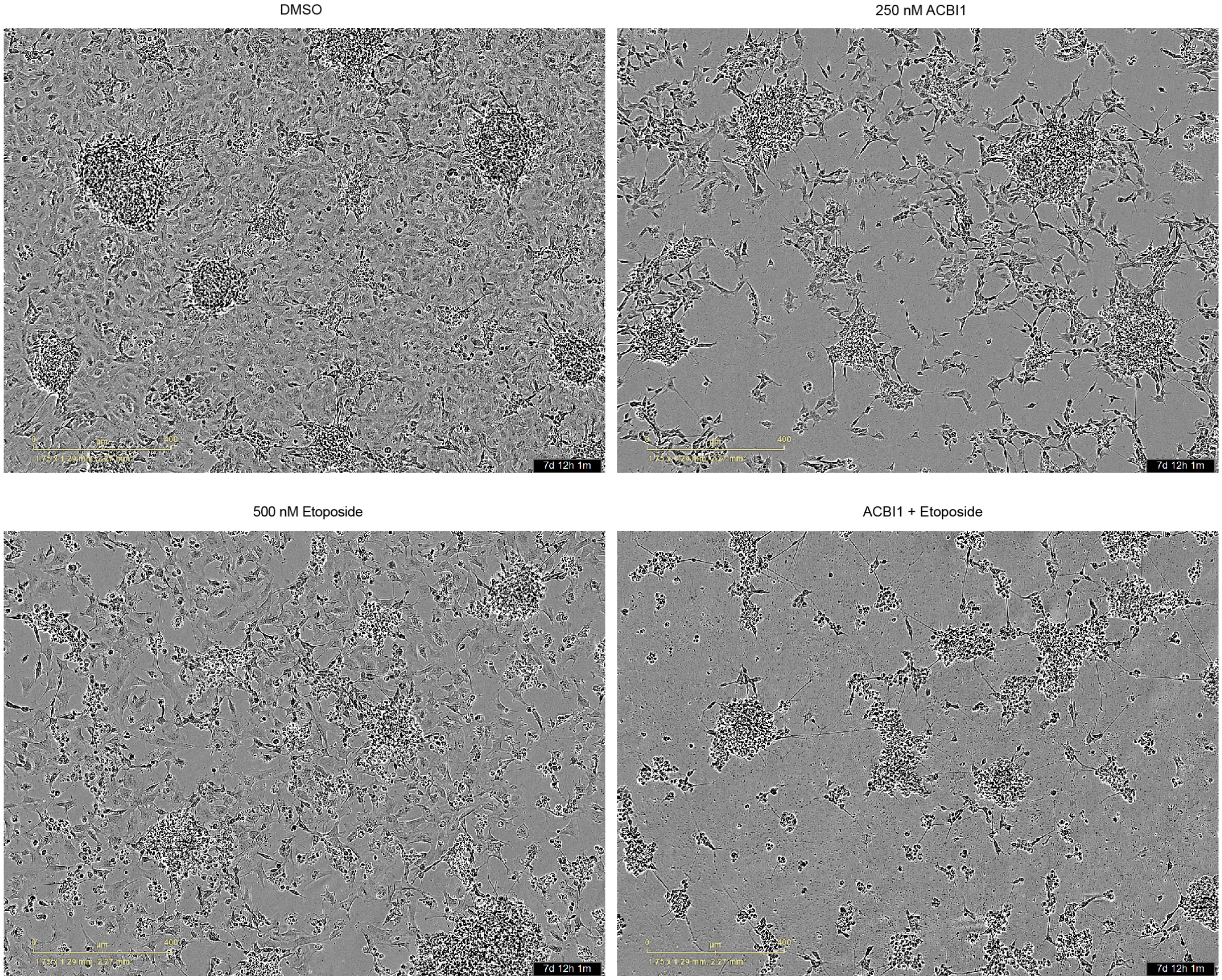

Fig. S6 continued

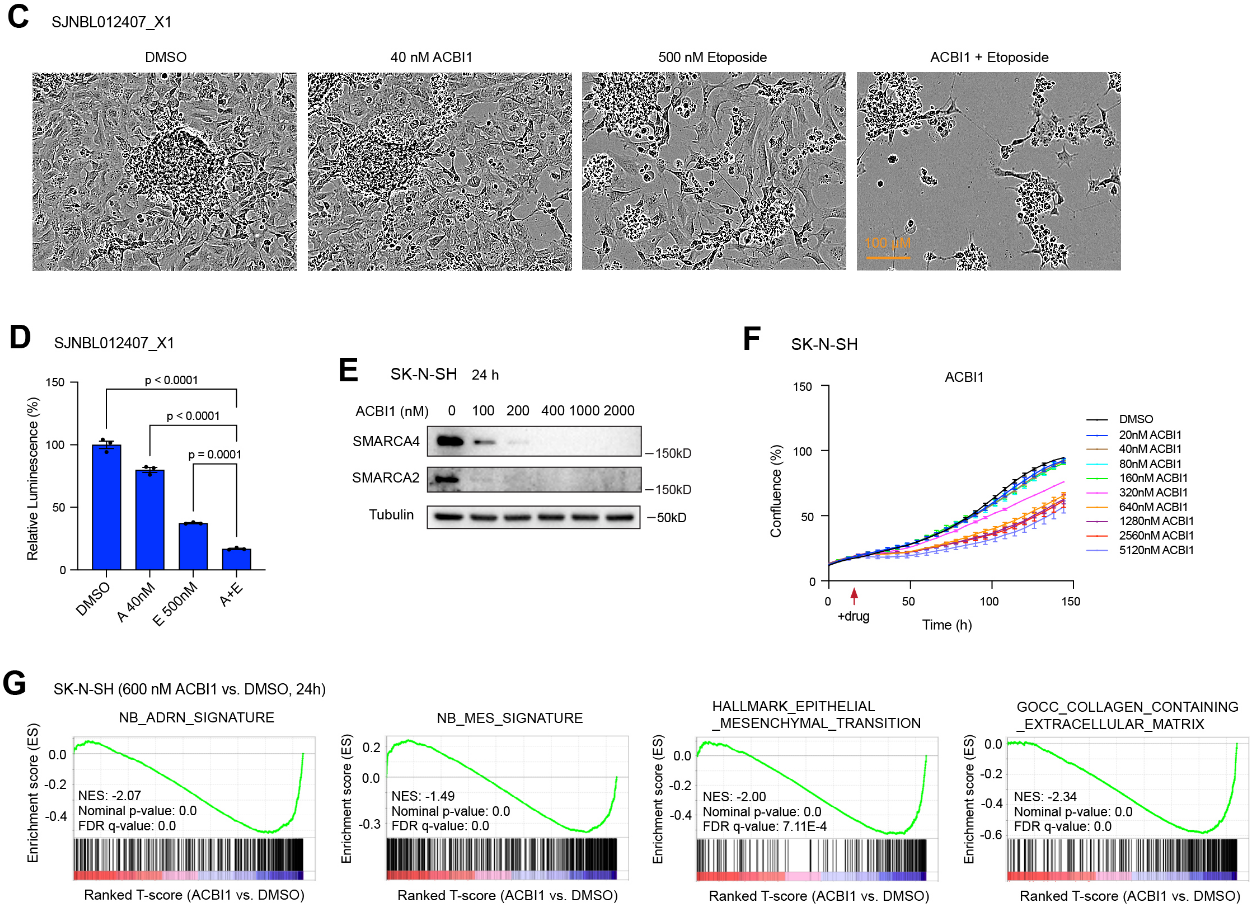

Appendix Figure S6: Sensitization of NB cells to chemotherapeutic drug treatment through depletion of SWI/SNF ATPases (supplementary to Fig. 6). (**A**) Protein levels of SMARCA2 and SMARCA4 detected by western blot assay in the SJNBL012407_X1 cells after ACBI1 treatment. **(B**) Representative uncropped images of SJNBL012407_X1 PDX cells treated with DMSO, 250 nM ACBI1, 500 nM etoposide, and ACBI1 + etoposide are presented. Cell images reveal distinct changes, with DMSO-treated cells showing spheres, as well as enlargement and flattening of MES-type monolayer cells. ACBI1-treated cells predominantly exhibit spheres and neuroblast-like cells, with a rare presence of flattened, enlarged MES-type cells. Etoposide-treated cells exhibit a reduced number of ADRN-type cells, but a substantial population of MES-type cells persists in the cultures. In contrast, cells treated with a combination of ACBI1 and etoposide exhibit predominantly unhealthy or decreased cells, indicated by the altered cell morphology. (**C**) Representative images of SJNBL012407_X1 PDX cells treated with DMSO (a vehicle control), 40 nM ACBI1, 500 nM etoposide, and ACBI1 + etoposide are presented. These images are captured using the IncuCyte SX5 imaging system. DMSO-treated cells showing spheres, as well as enlargement and flattening of MES-type monolayer cells. Like DMSO-treated group, 40 nM ACBI1-treated cells exhibit spheres and MES-type monolayer cells. Etoposide-treated cells exhibit a reduced number of ADRN-type cells, but a substantial population of MES-type cells persists in the cultures. In contrast, cells treated with a combination of ACBI1 and etoposide exhibit predominantly unhealthy or decreased cells, indicated by the altered cell morphology. (**D**) CellTiter-Glo assay measures the effect of 40 nM ACBI1 or 500 nM etoposide, or ACBI1 plus etoposide treatment on cell growth. Cell viabilities of DMSO-treated cells are set to 100%, and the bar graph is generated using GraphPad Prism software. (**E**) Protein levels of SMARCA2 and SMARCA4 detected by western blot assay in the SK-N-SH cell line after ACBI1 treatment. (**F**) ACBI1

treatment of SK-N-SH reduces cell proliferation indicated by IncuCyte confluence assay. **(G)** GSEA of bulk RNA-seq shows that SWI/SNF ATPases depletion in SK-N-SH cell line leads to negative enrichment of ADRN, MES, EMT, and CCEM signature genes. Note: In panel **(D)**, the data are represented as mean ± SEM. Statistical differences were calculated using ordinary one-way ANOVA.
