# Supplementary material for: Targeting SWI/SNF ATPases reduces neuroblastoma cell plasticity
Source: EMBO J. 2024 Aug 22;43(20):4522–41. doi: 10.1038/s44318-024-00206-1 (PMC11480351; doi:10.1038/s44318-024-00206-1)
Supplement: Supplementary file 14 — Movie EV2 [file 44318_2024_206_MOESM14_ESM.zip › EMBOJ-2023-115707_MovieEV2/Movie EV2 Legend.docx]

**Movie EV2**

This movie shows the time-lapse cell images indicating rarely observed MES-type monolayer cells in ACBI1-treated SJNBL012407_X1 cells.
