# Supplementary figures and images for: Targeting SWI/SNF ATPases reduces neuroblastoma cell plasticity
Source: EMBO J. 2024 Aug 22;43(20):4522–41. doi: 10.1038/s44318-024-00206-1 (PMC11480351; doi:10.1038/s44318-024-00206-1)

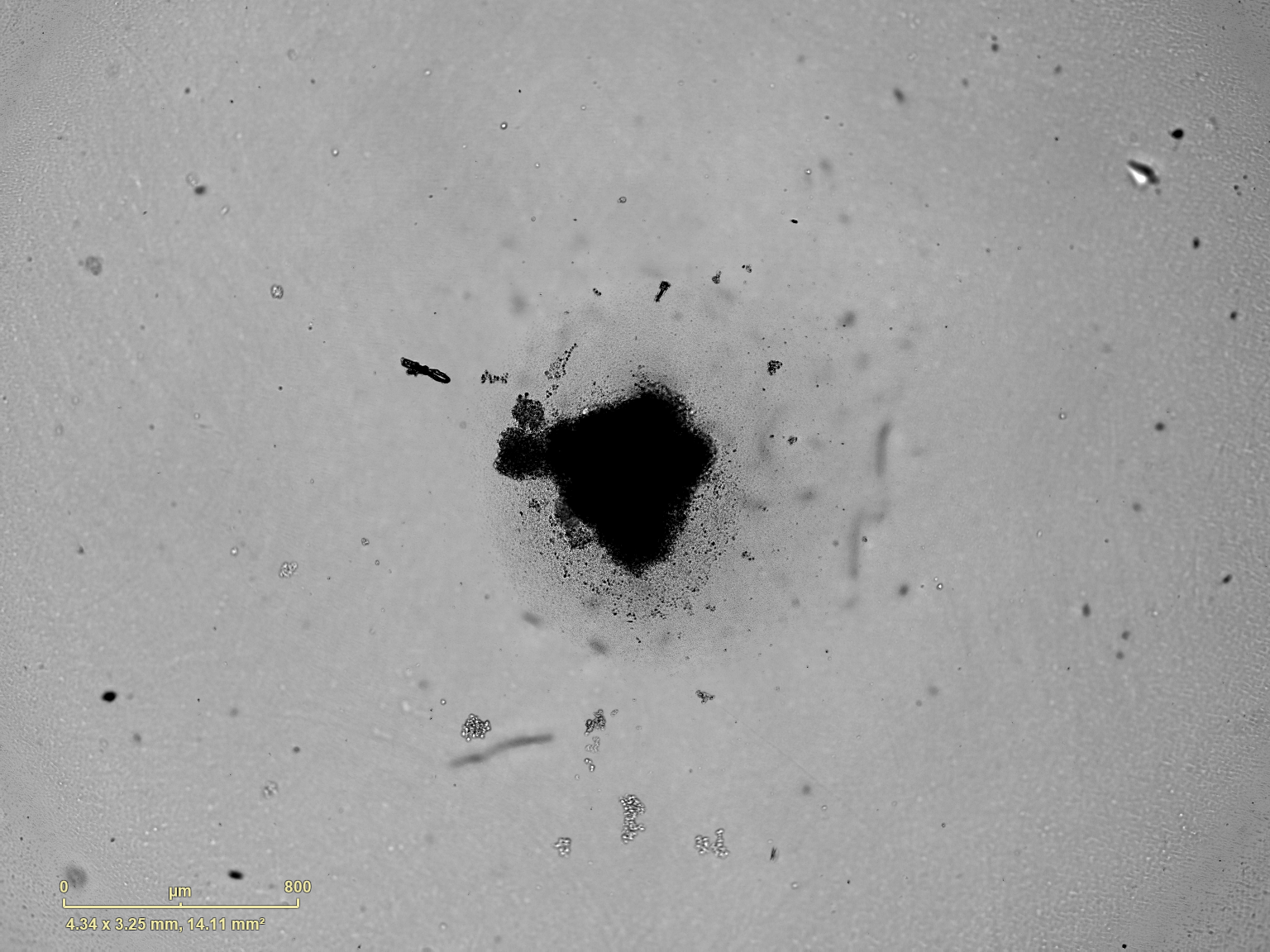

Supplement: Supplementary file 15 — Source data Fig. 1 [file 44318_2024_206_MOESM15_ESM.zip › Figure 1/1G/IMR5 - ACBI1.tif]

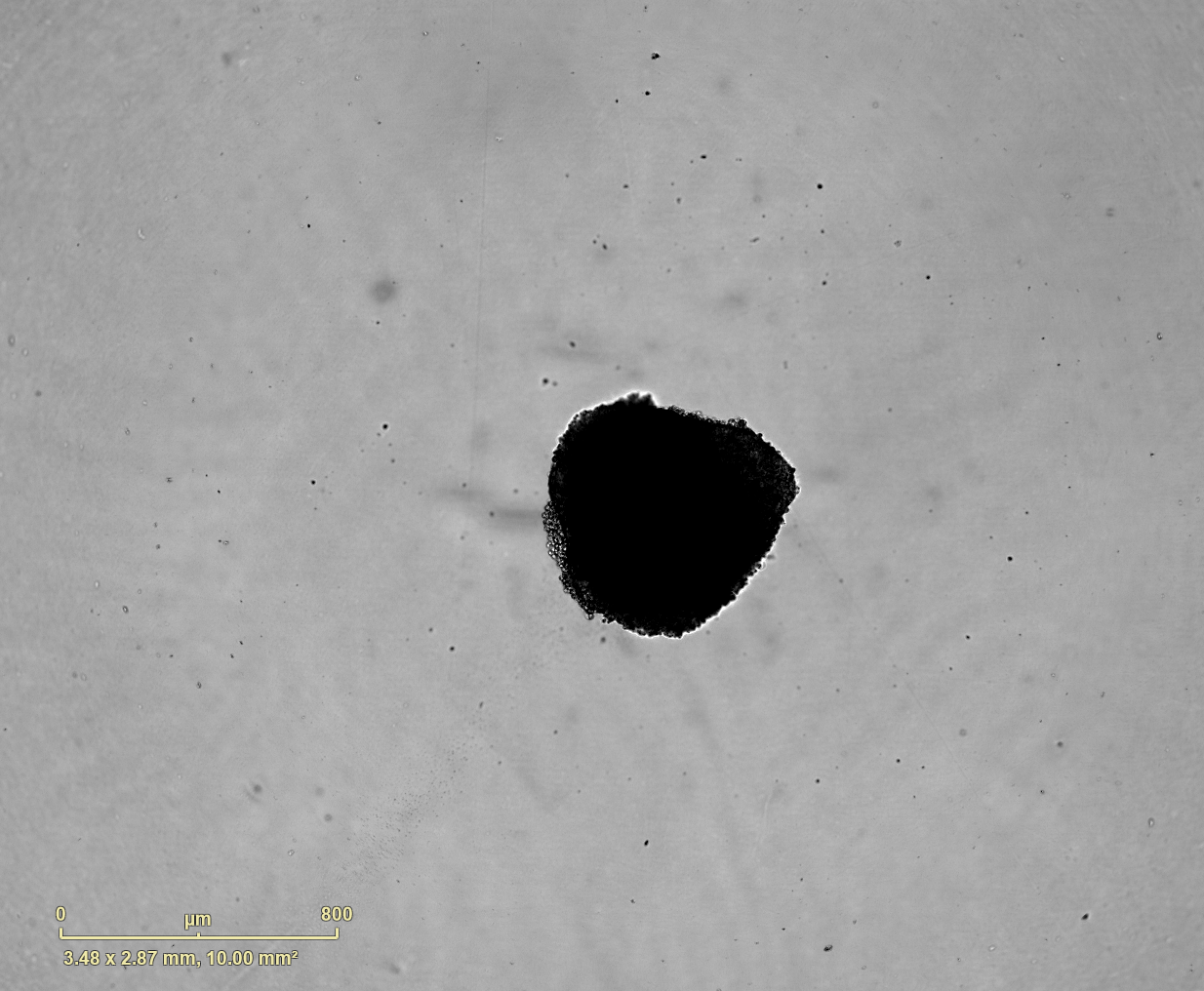

Supplement: Supplementary file 15 — Source data Fig. 1 [file 44318_2024_206_MOESM15_ESM.zip › Figure 1/1G/IMR32 - DMSO.tif]

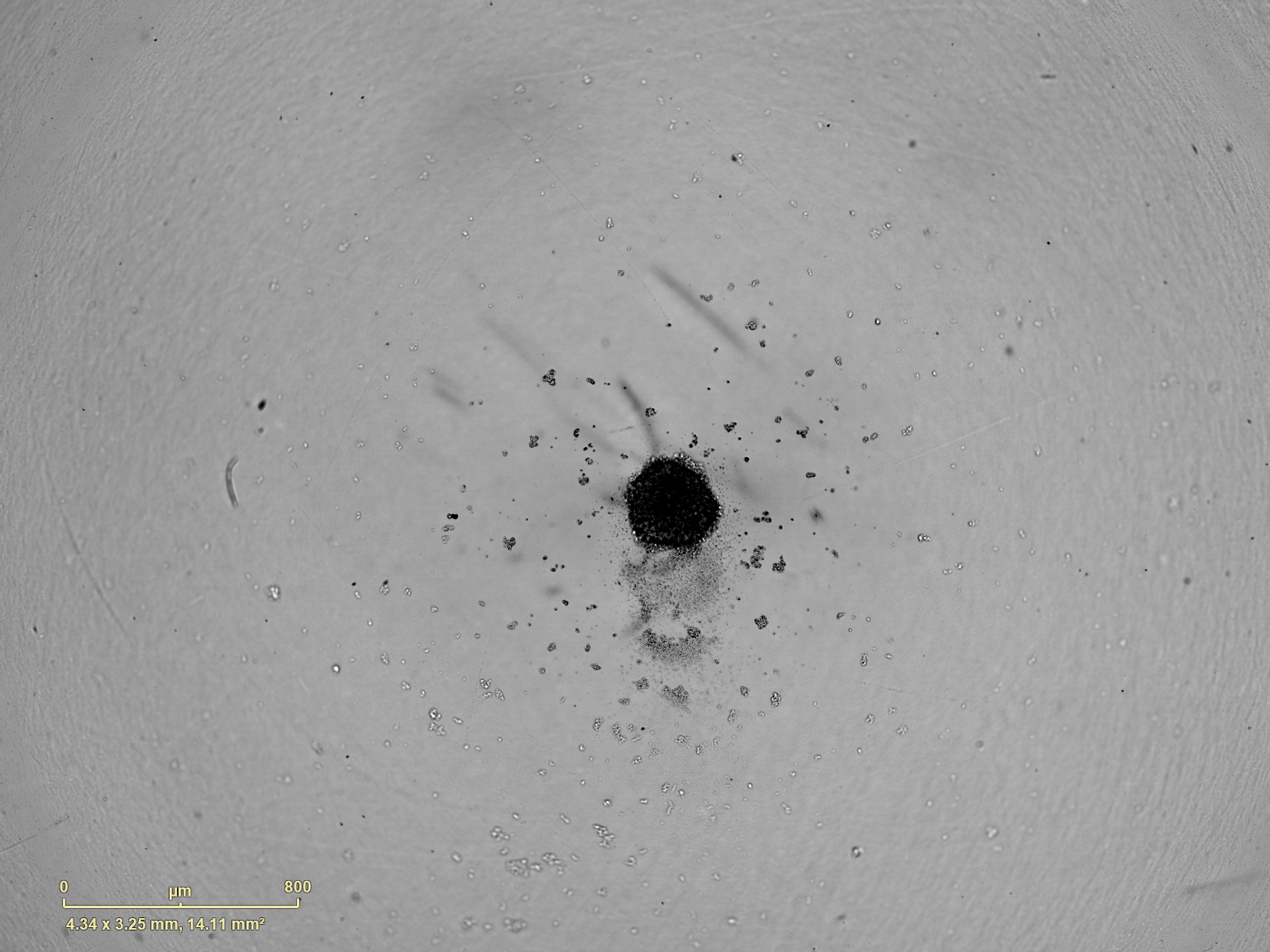

Supplement: Supplementary file 15 — Source data Fig. 1 [file 44318_2024_206_MOESM15_ESM.zip › Figure 1/1G/SY5Y - ACBI1.tif]

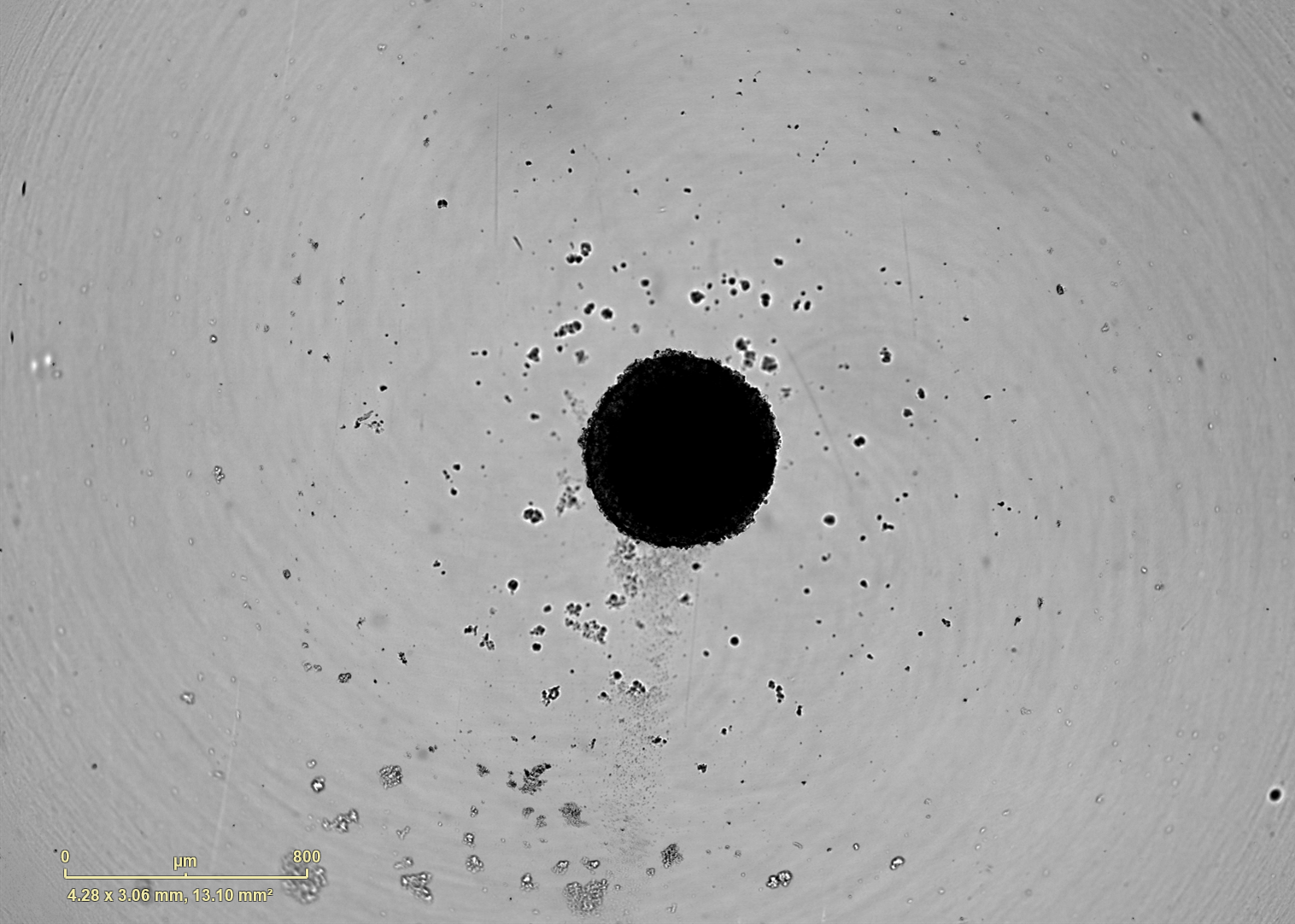

Supplement: Supplementary file 15 — Source data Fig. 1 [file 44318_2024_206_MOESM15_ESM.zip › Figure 1/1G/KCNR - DMSO.tif]

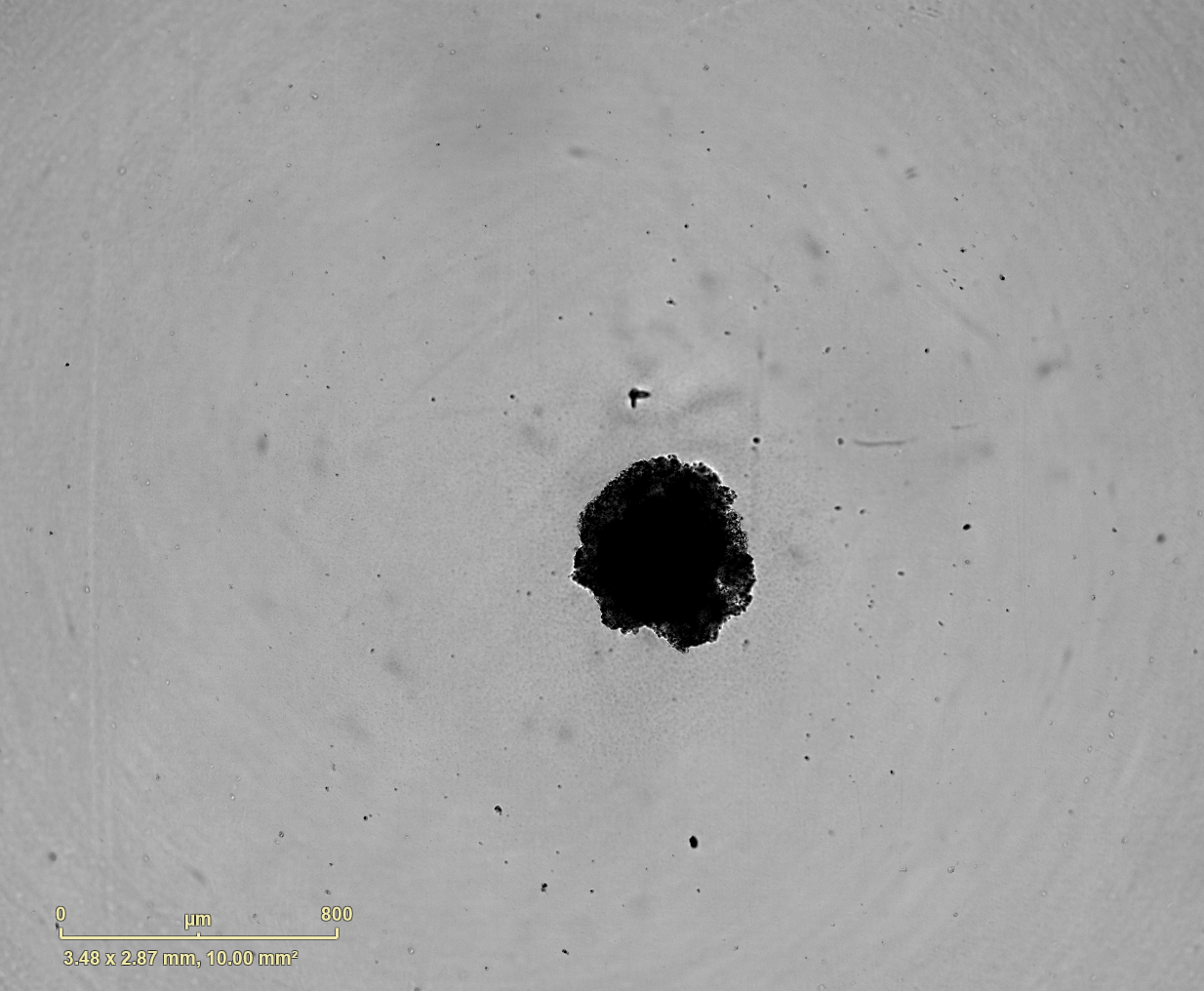

Supplement: Supplementary file 15 — Source data Fig. 1 [file 44318_2024_206_MOESM15_ESM.zip › Figure 1/1G/IMR32 - ACBI1.tif]

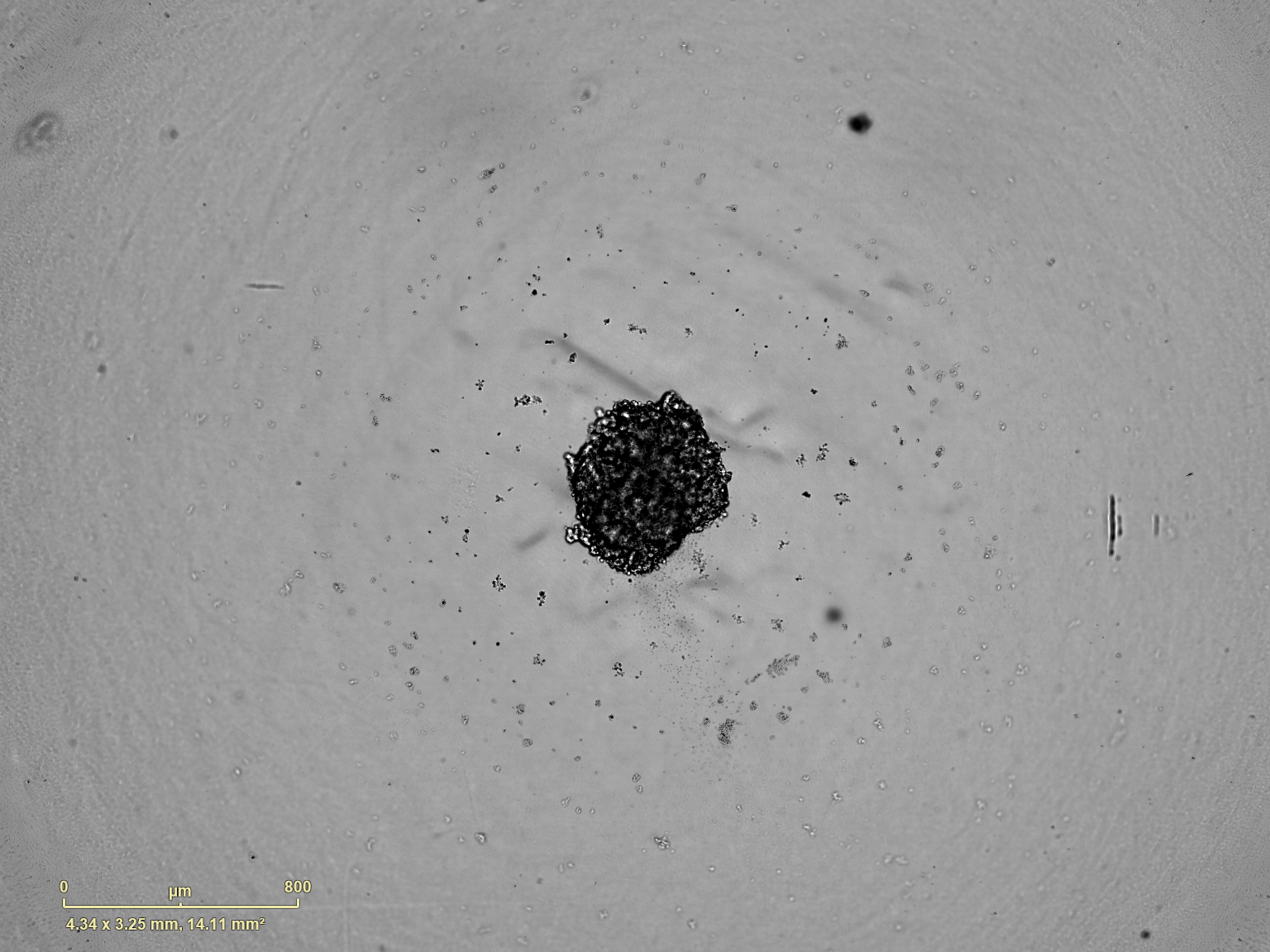

Supplement: Supplementary file 15 — Source data Fig. 1 [file 44318_2024_206_MOESM15_ESM.zip › Figure 1/1G/SY5Y - DMSO.tif]

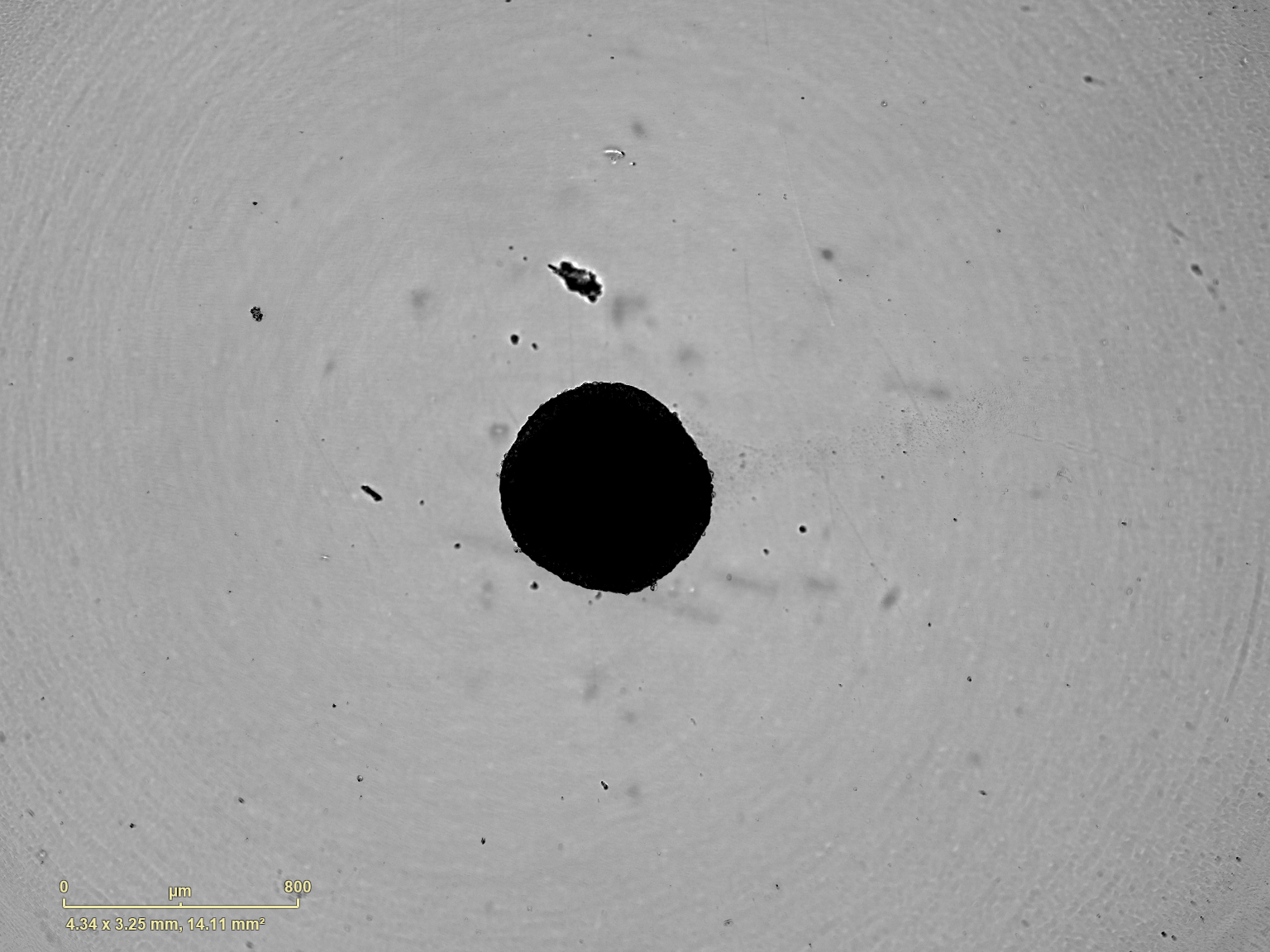

Supplement: Supplementary file 15 — Source data Fig. 1 [file 44318_2024_206_MOESM15_ESM.zip › Figure 1/1G/IMR5 - DMSO.tif]

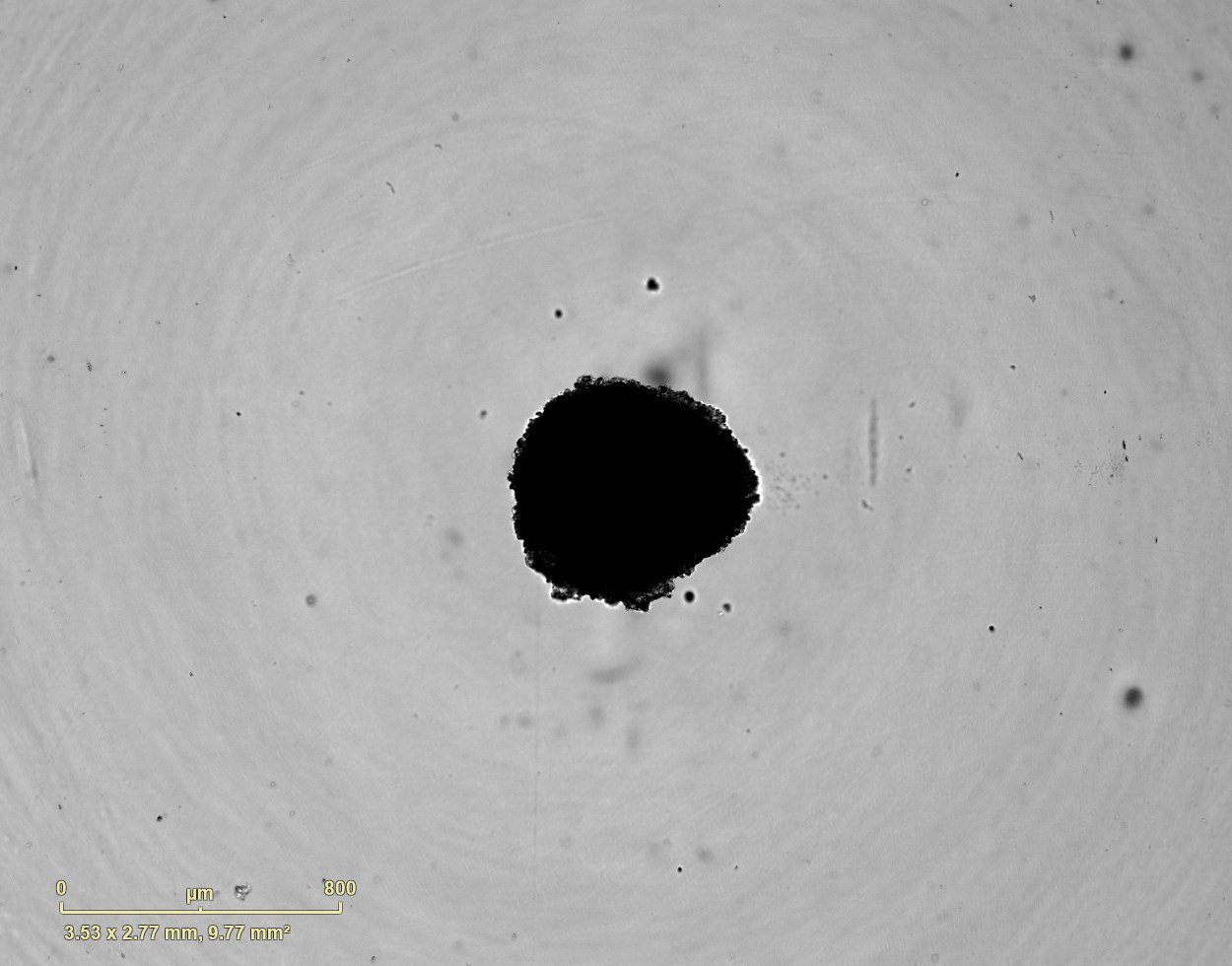

Supplement: Supplementary file 15 — Source data Fig. 1 [file 44318_2024_206_MOESM15_ESM.zip › Figure 1/1G/BE2C - AU-15330.tif]

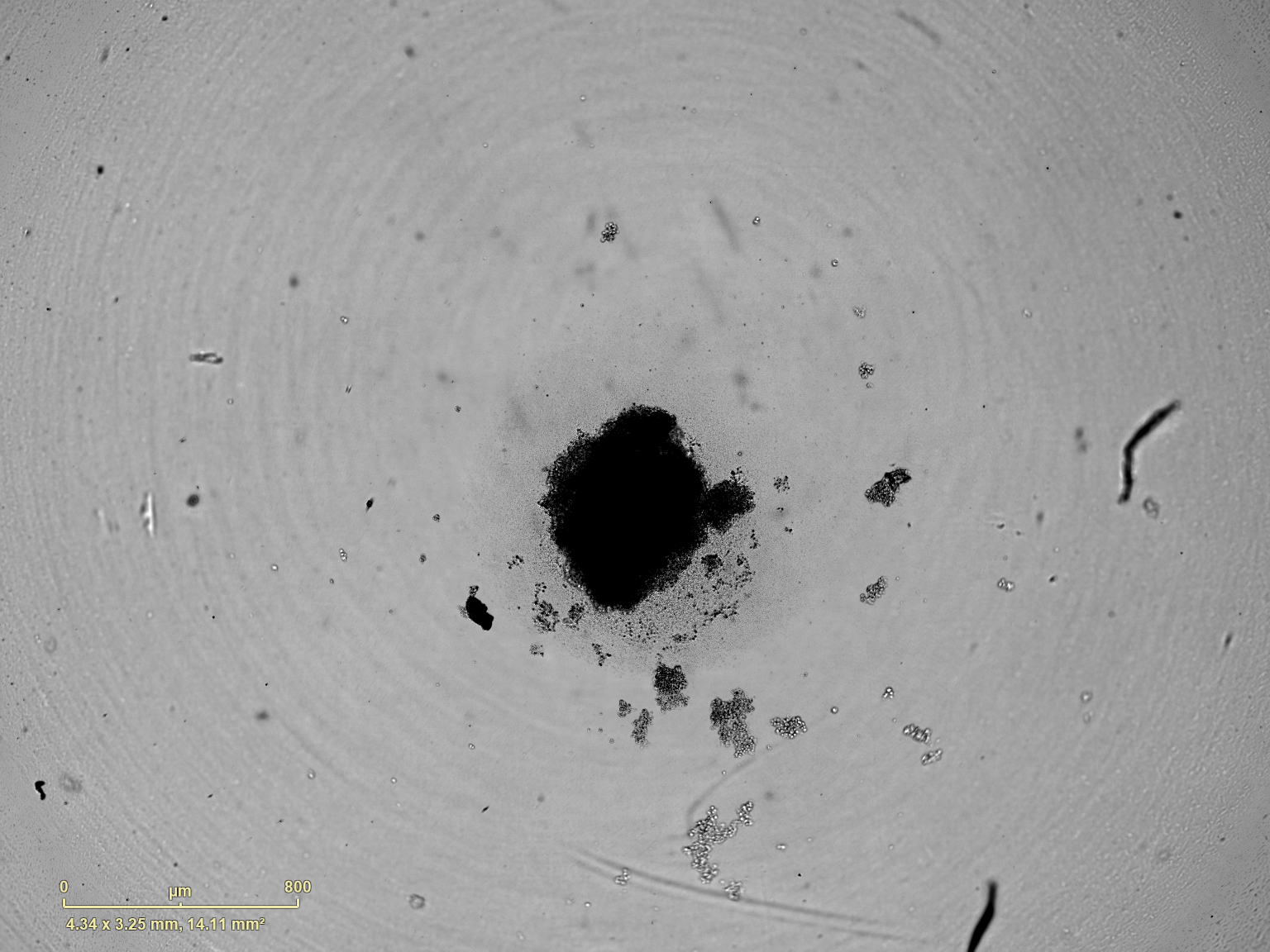

Supplement: Supplementary file 15 — Source data Fig. 1 [file 44318_2024_206_MOESM15_ESM.zip › Figure 1/1G/IMR5 - AU-15330.tif]

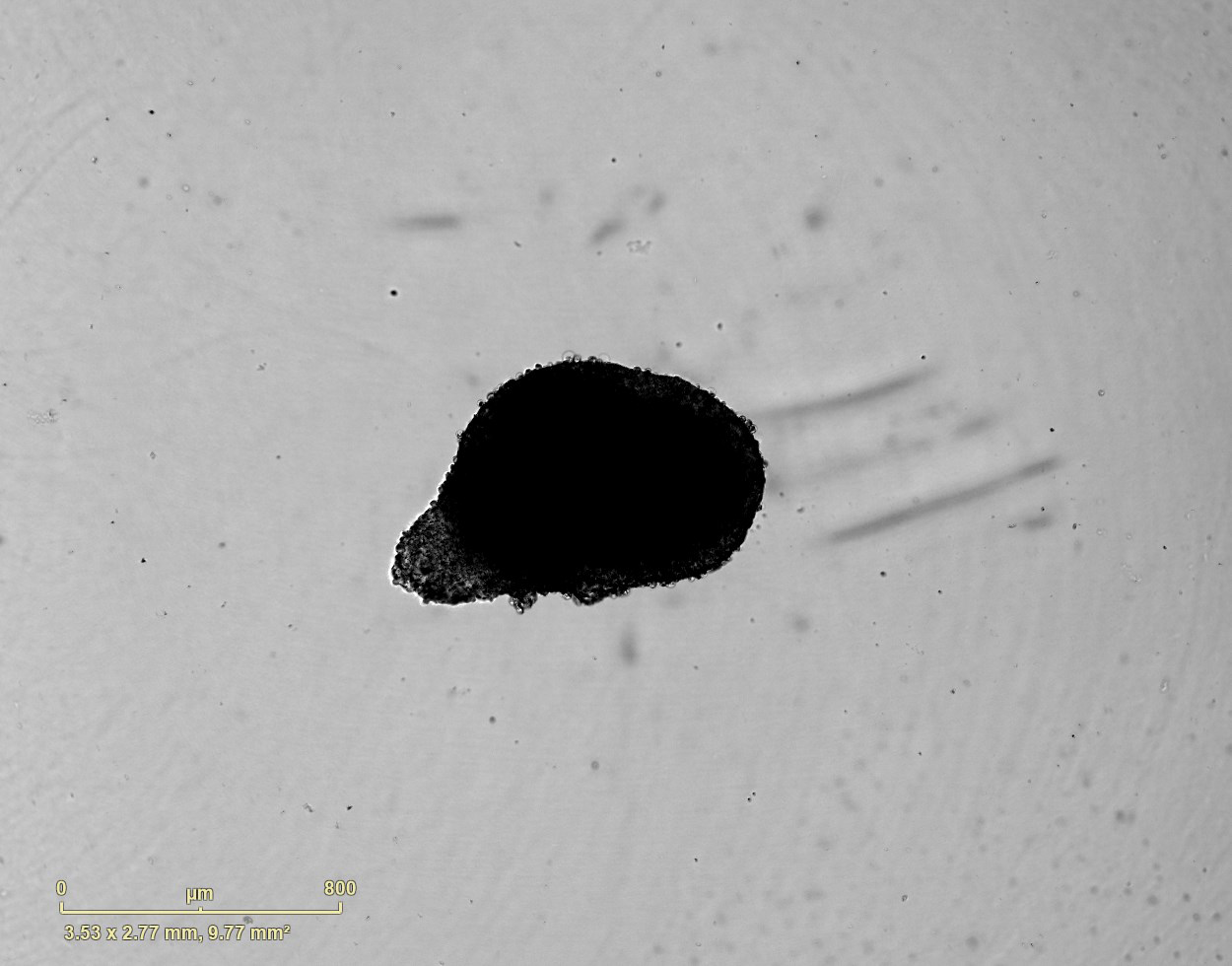

Supplement: Supplementary file 15 — Source data Fig. 1 [file 44318_2024_206_MOESM15_ESM.zip › Figure 1/1G/BE2C - DMSO.tif]

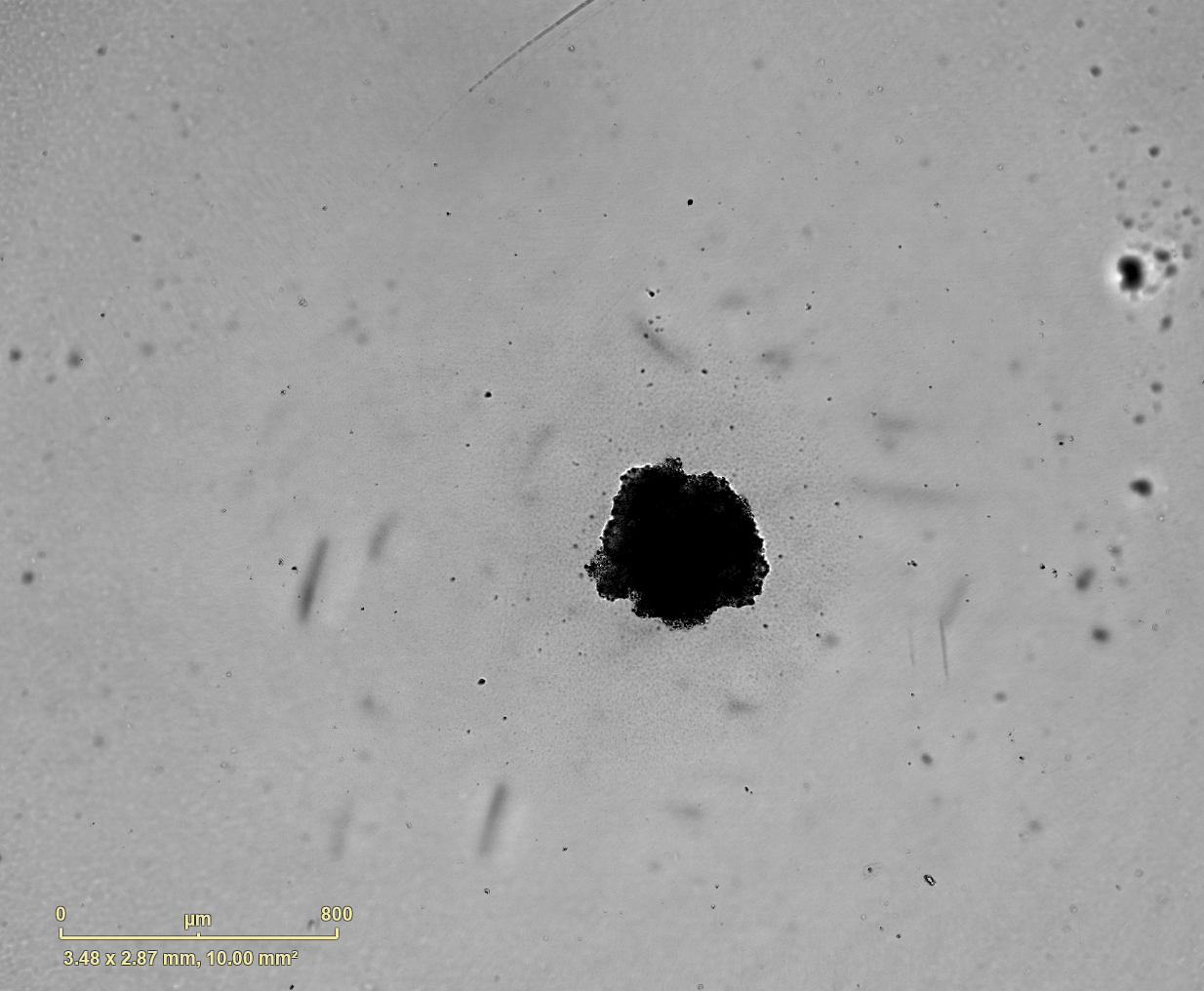

Supplement: Supplementary file 15 — Source data Fig. 1 [file 44318_2024_206_MOESM15_ESM.zip › Figure 1/1G/IMR32 - AU-15330.tif]

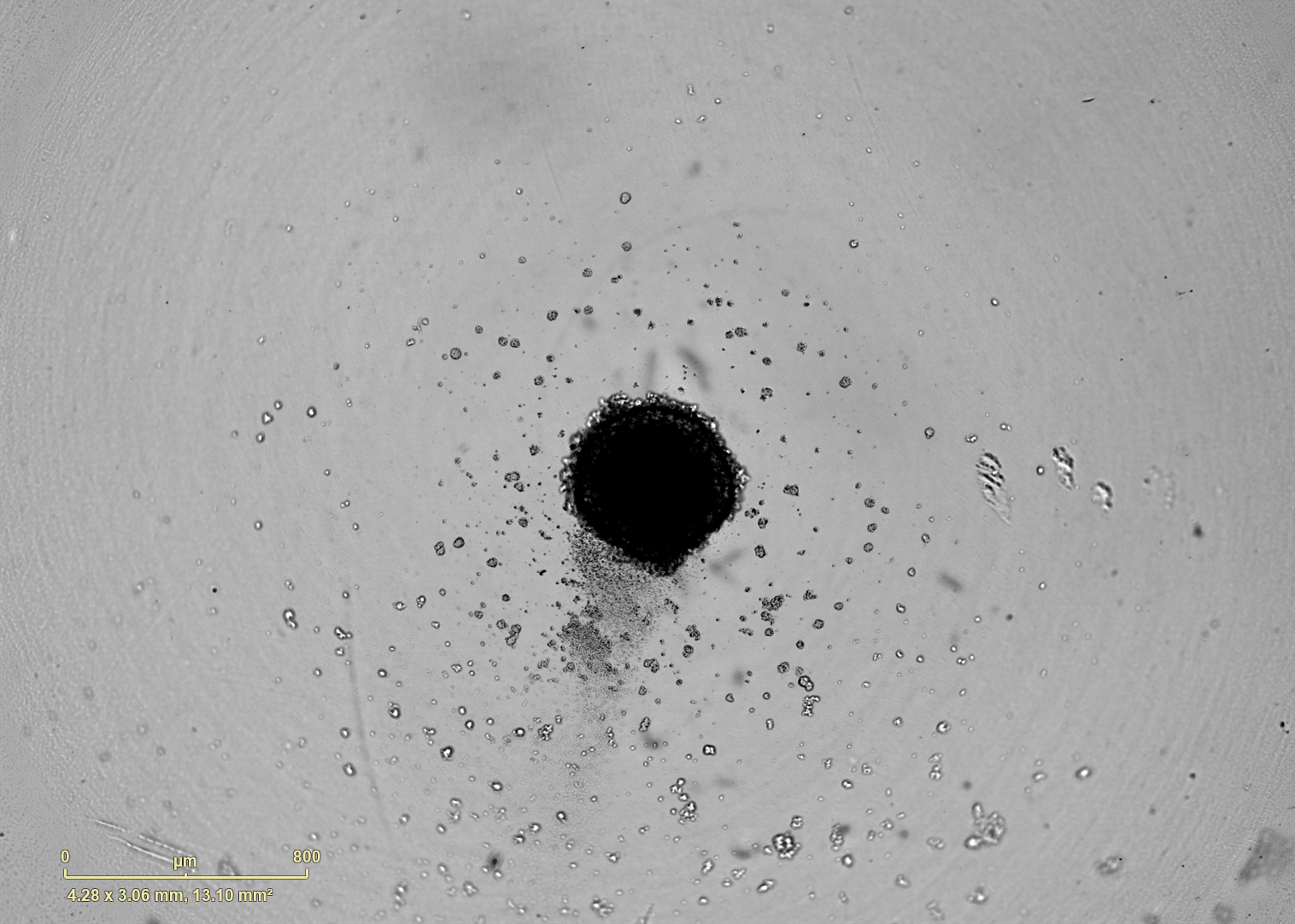

Supplement: Supplementary file 15 — Source data Fig. 1 [file 44318_2024_206_MOESM15_ESM.zip › Figure 1/1G/KCNR - ACBI1.tif]

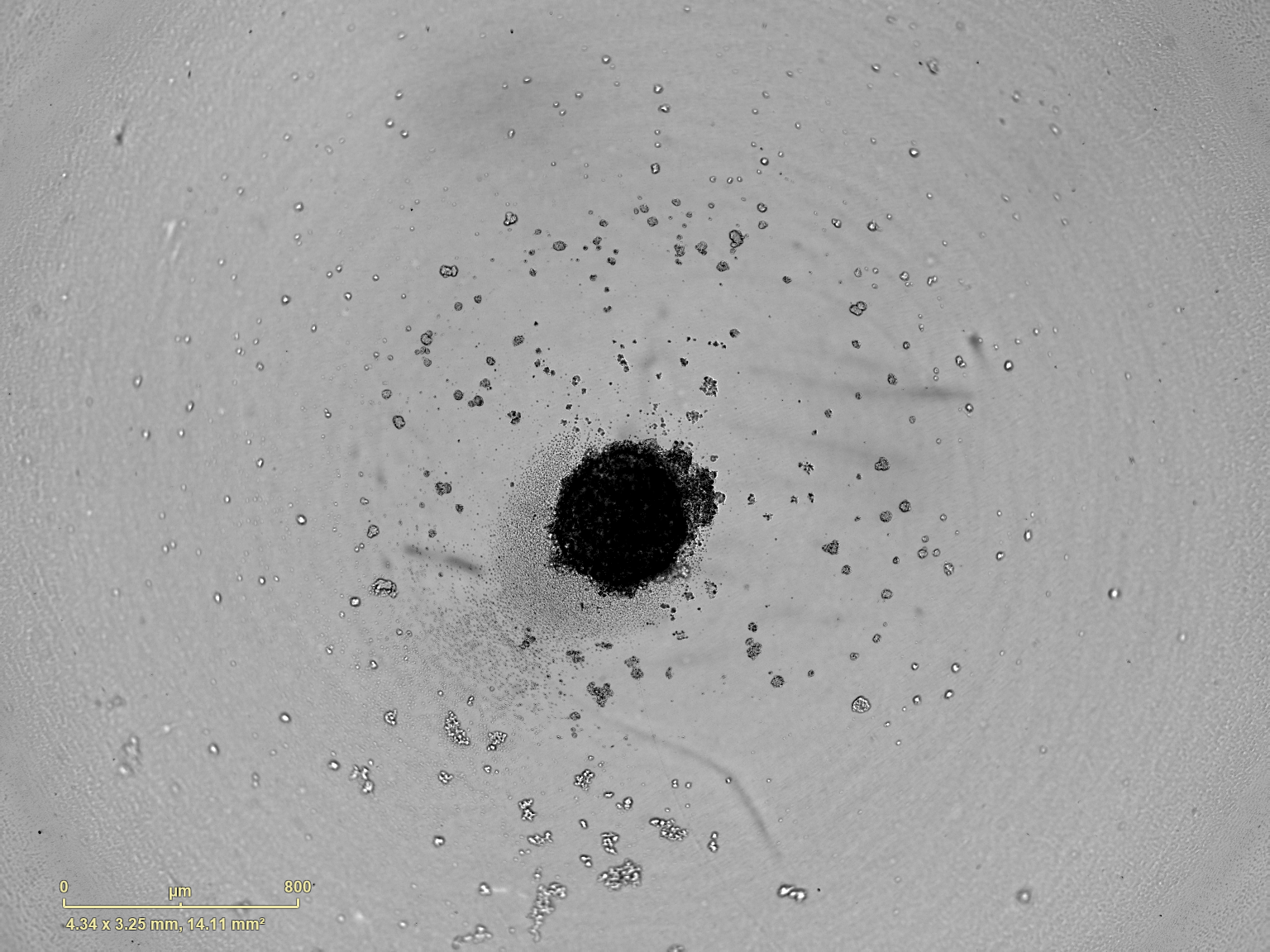

Supplement: Supplementary file 15 — Source data Fig. 1 [file 44318_2024_206_MOESM15_ESM.zip › Figure 1/1G/KCNR - AU-15330.tif]

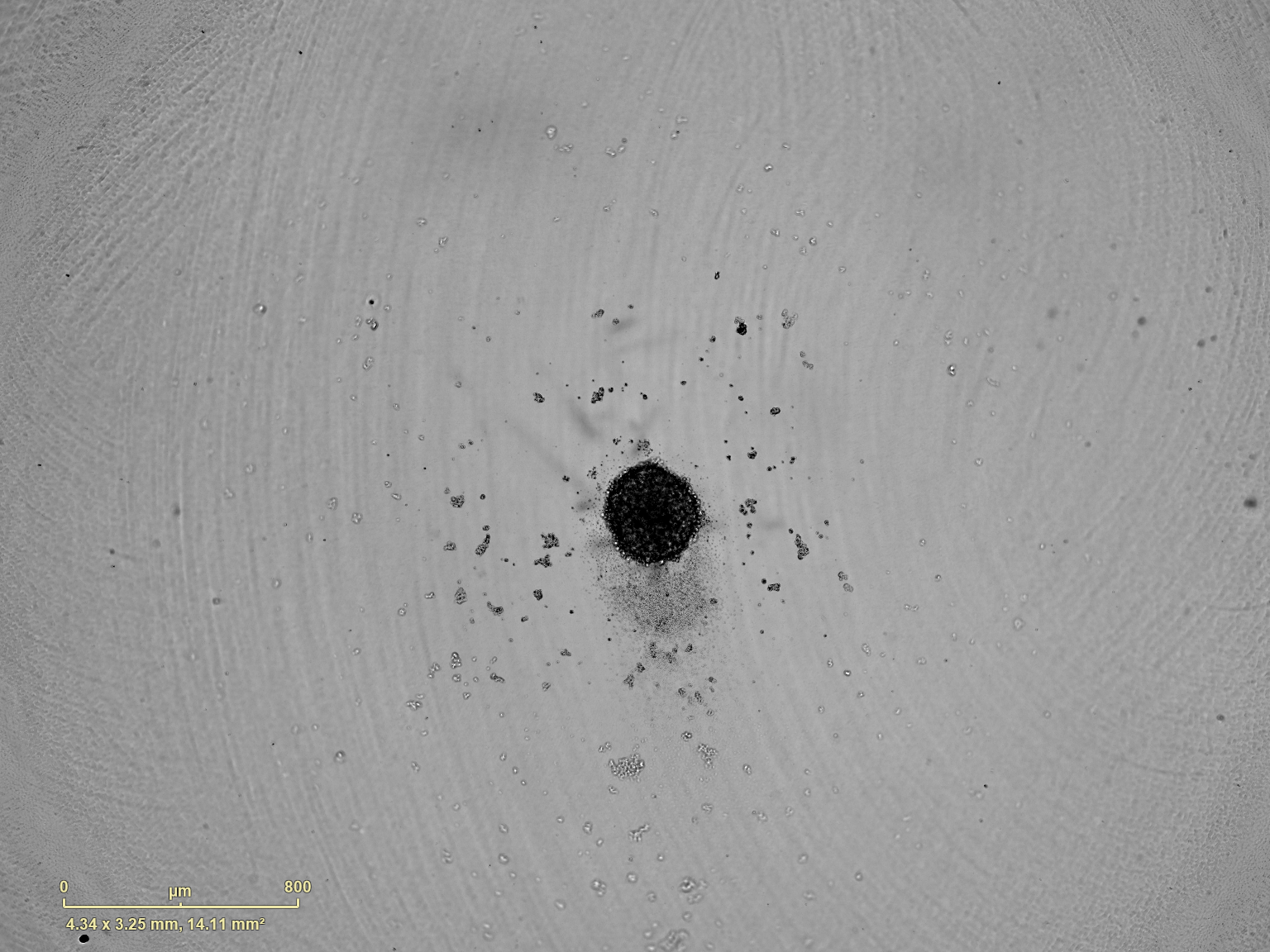

Supplement: Supplementary file 15 — Source data Fig. 1 [file 44318_2024_206_MOESM15_ESM.zip › Figure 1/1G/SY5Y - AU-15330.tif]

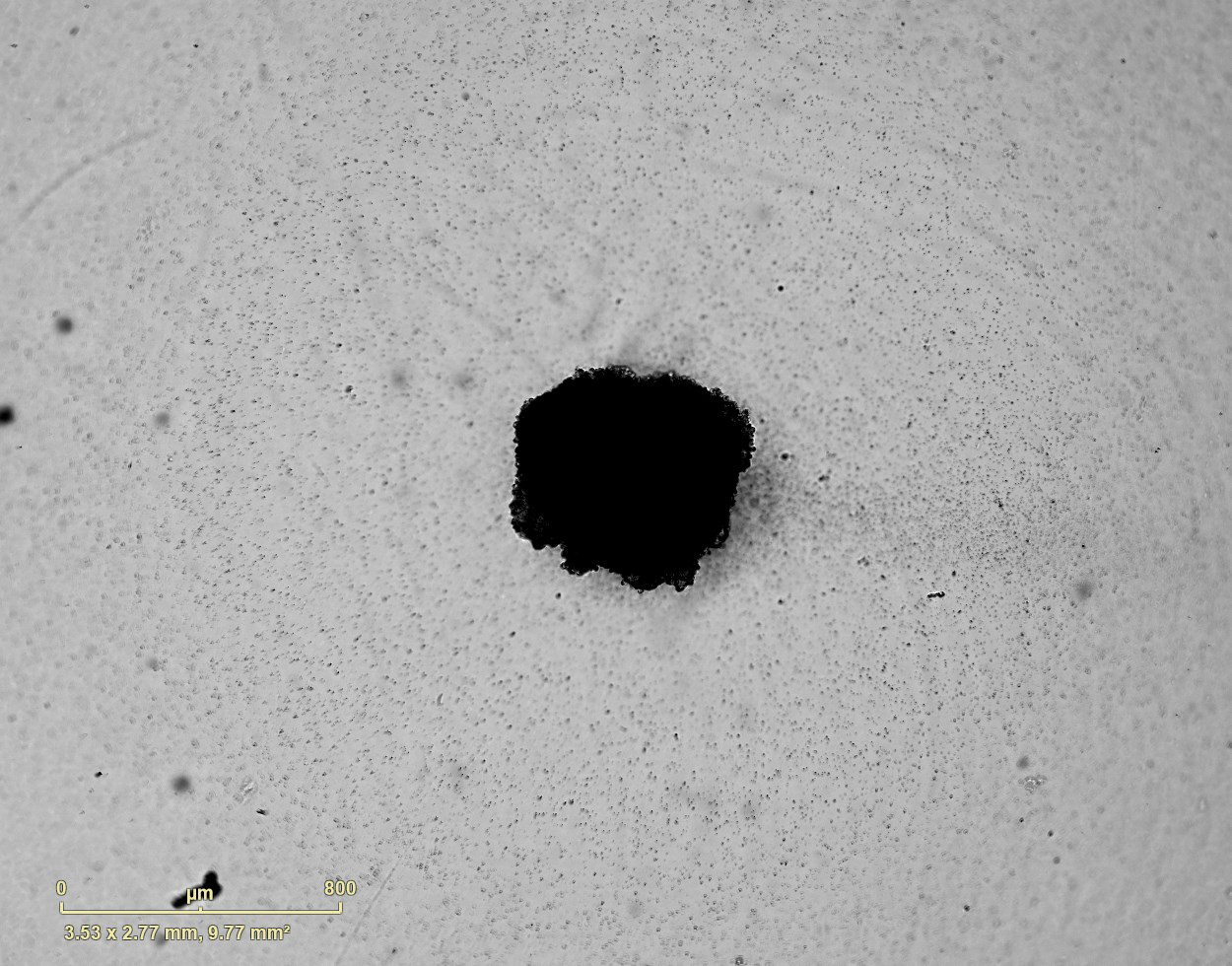

Supplement: Supplementary file 15 — Source data Fig. 1 [file 44318_2024_206_MOESM15_ESM.zip › Figure 1/1G/BE2C - ACBI1.tif]

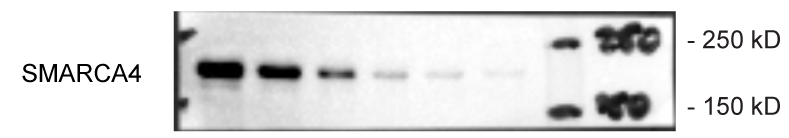

Supplement: Supplementary file 15 — Source data Fig. 1 [file 44318_2024_206_MOESM15_ESM.zip › Figure 1/1D/Western SMARCA4.tif]

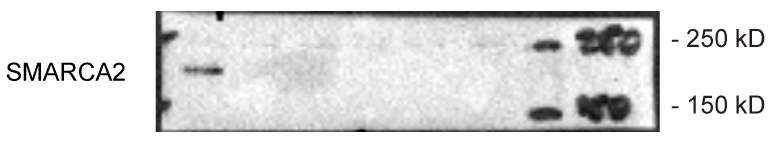

Supplement: Supplementary file 15 — Source data Fig. 1 [file 44318_2024_206_MOESM15_ESM.zip › Figure 1/1D/Western SMARCA2.tif]

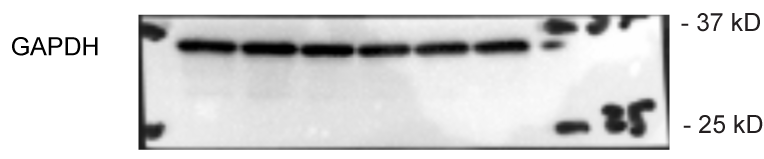

Supplement: Supplementary file 15 — Source data Fig. 1 [file 44318_2024_206_MOESM15_ESM.zip › Figure 1/1D/Western GAPDH.tif]

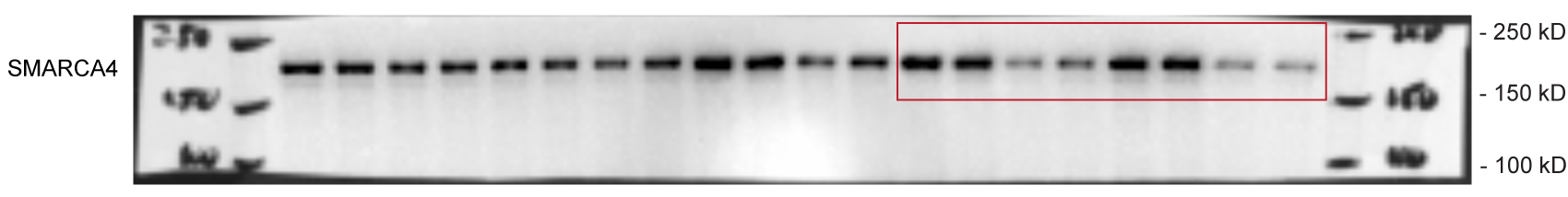

Supplement: Supplementary file 15 — Source data Fig. 1 [file 44318_2024_206_MOESM15_ESM.zip › Figure 1/1C/Western SMARCA4.tif]

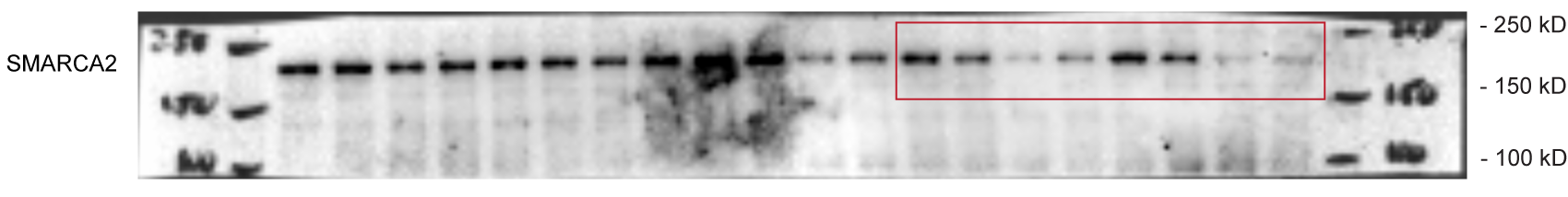

Supplement: Supplementary file 15 — Source data Fig. 1 [file 44318_2024_206_MOESM15_ESM.zip › Figure 1/1C/Western SMARCA2.tif]

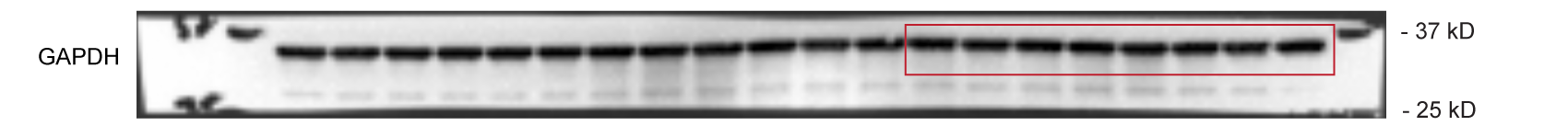

Supplement: Supplementary file 15 — Source data Fig. 1 [file 44318_2024_206_MOESM15_ESM.zip › Figure 1/1C/Western GAPDH.tif]

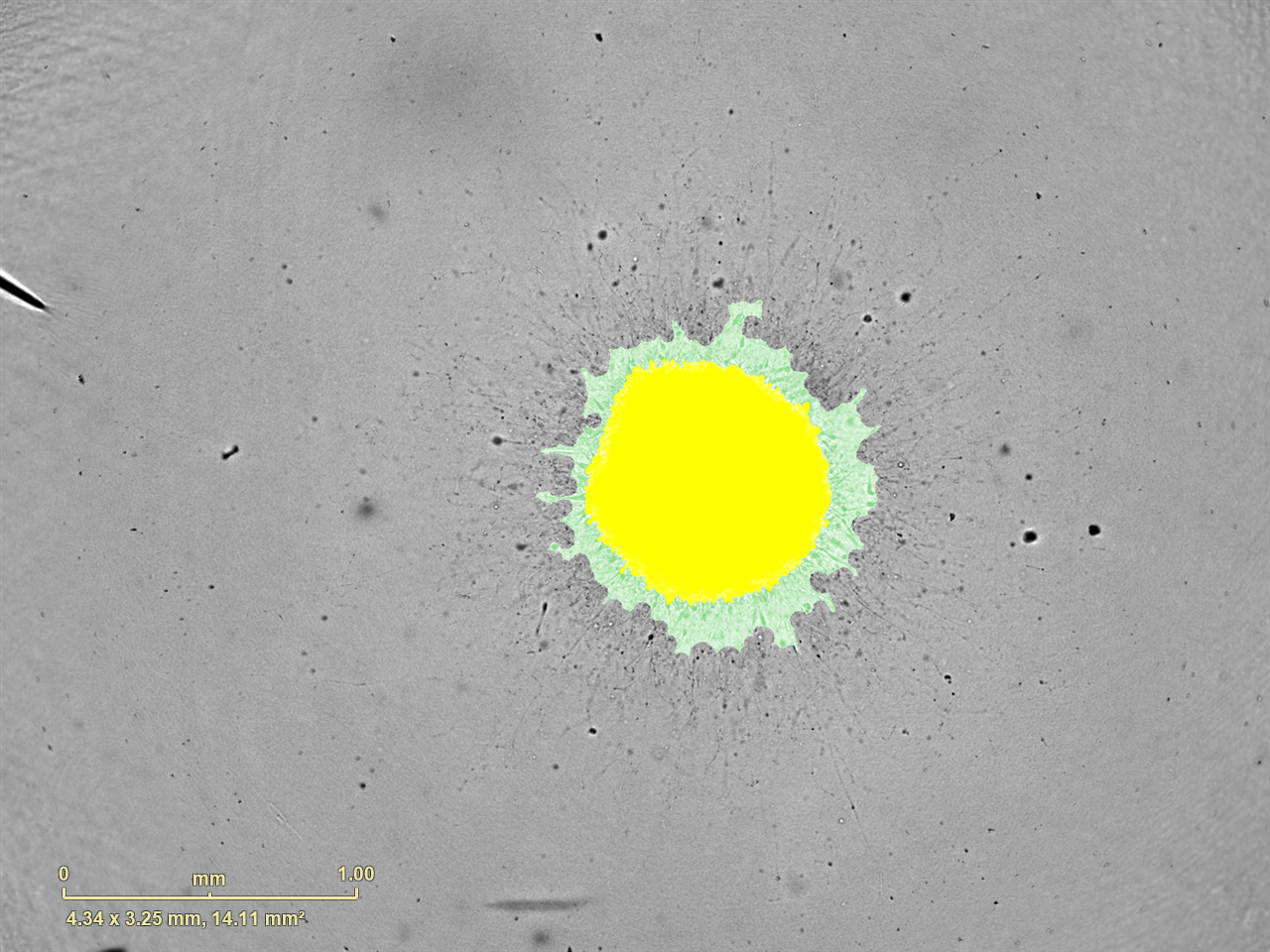

Supplement: Supplementary file 16 — Source data Fig. 3 [file 44318_2024_206_MOESM16_ESM.zip › Figure 3/3E/IMR32 invasion mask - DMSO.tif]

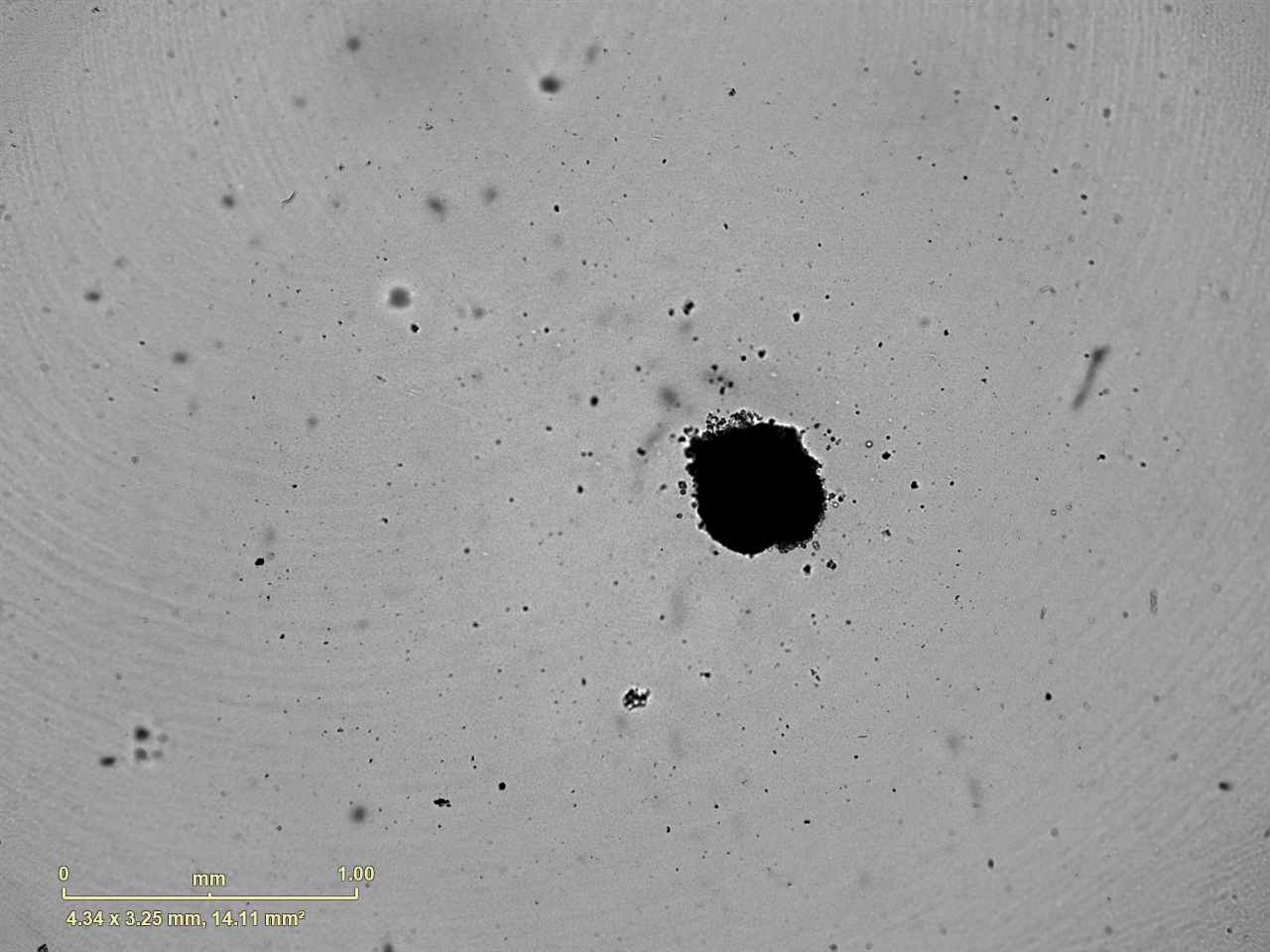

Supplement: Supplementary file 16 — Source data Fig. 3 [file 44318_2024_206_MOESM16_ESM.zip › Figure 3/3E/IMR32 Spheroid - ACBI1.tif]

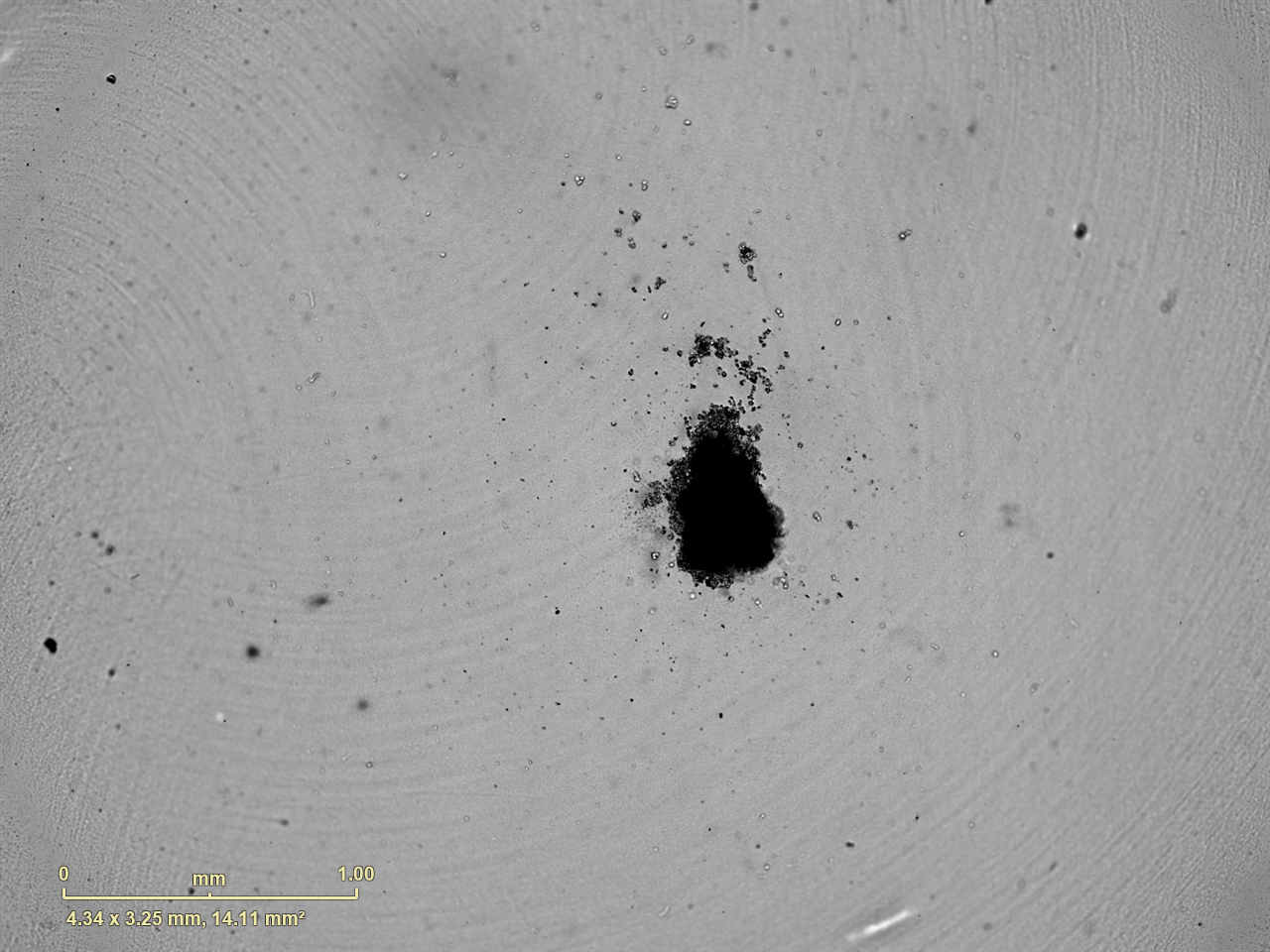

Supplement: Supplementary file 16 — Source data Fig. 3 [file 44318_2024_206_MOESM16_ESM.zip › Figure 3/3E/IMR32 Spheroid - AU-15330.tif]

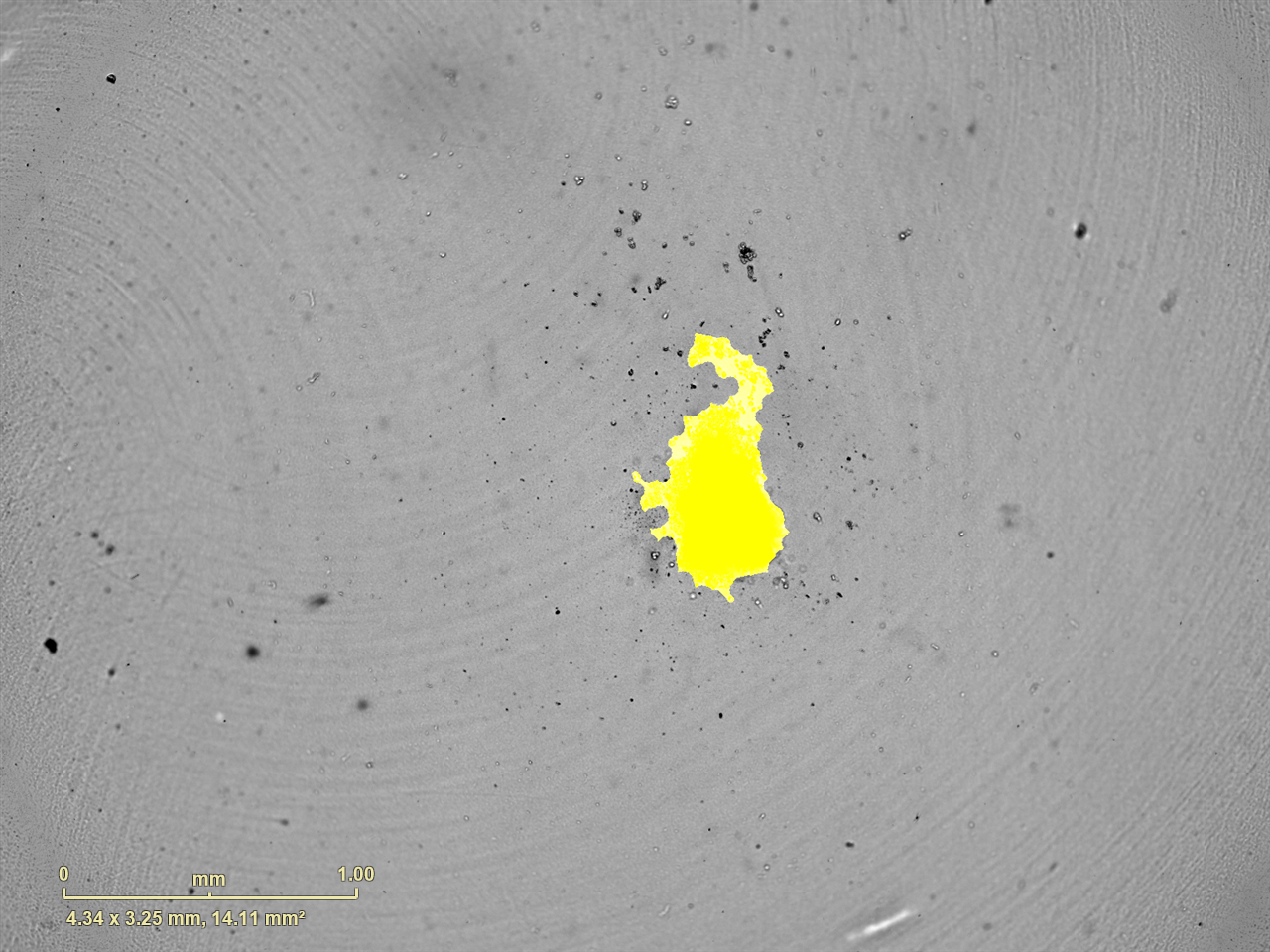

Supplement: Supplementary file 16 — Source data Fig. 3 [file 44318_2024_206_MOESM16_ESM.zip › Figure 3/3E/IMR32 invasion mask - AU-15330.tif]

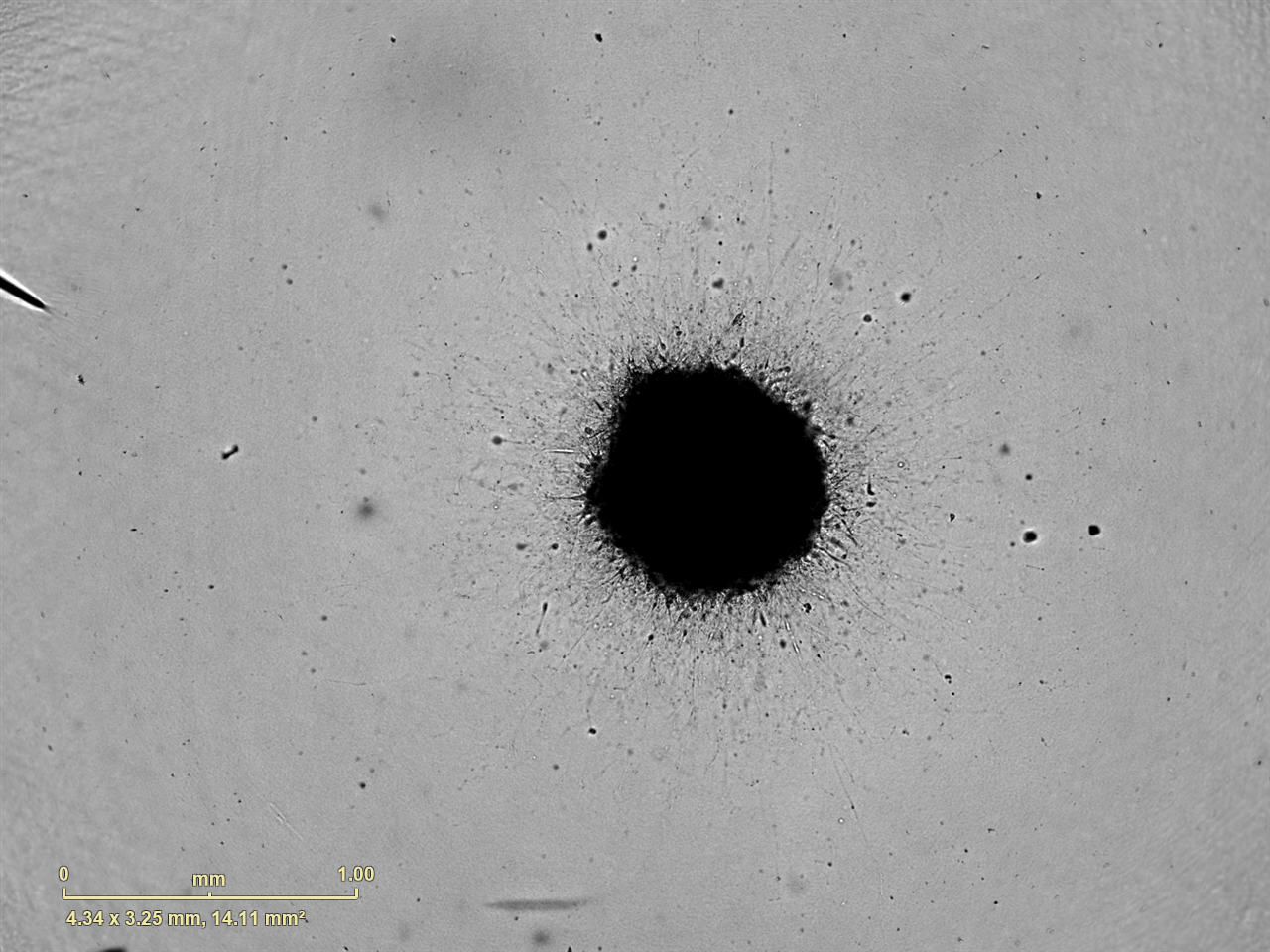

Supplement: Supplementary file 16 — Source data Fig. 3 [file 44318_2024_206_MOESM16_ESM.zip › Figure 3/3E/IMR32 Spheroid - DMSO.tif]

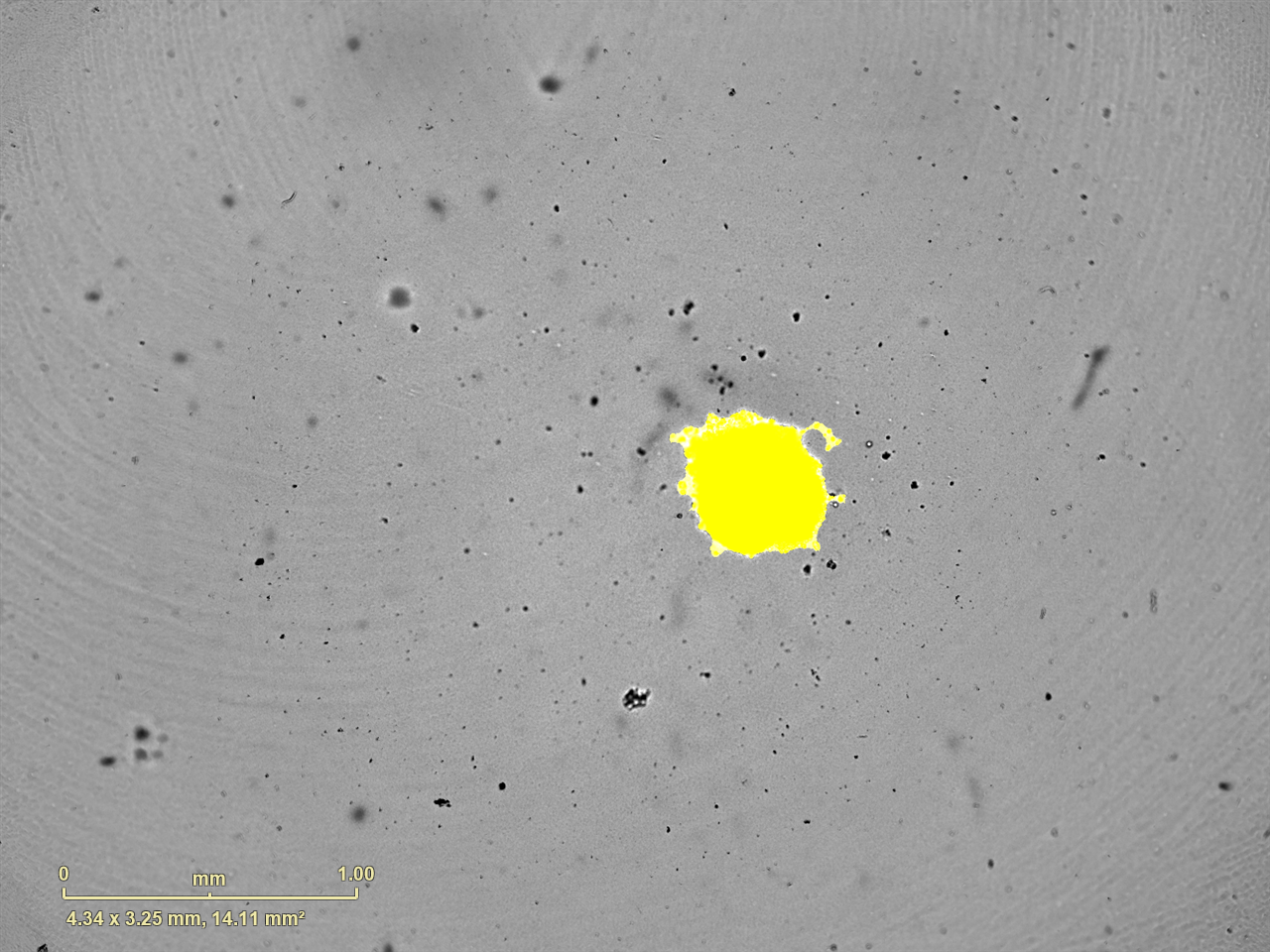

Supplement: Supplementary file 16 — Source data Fig. 3 [file 44318_2024_206_MOESM16_ESM.zip › Figure 3/3E/IMR32 invasion mask - ACBI1.tif]

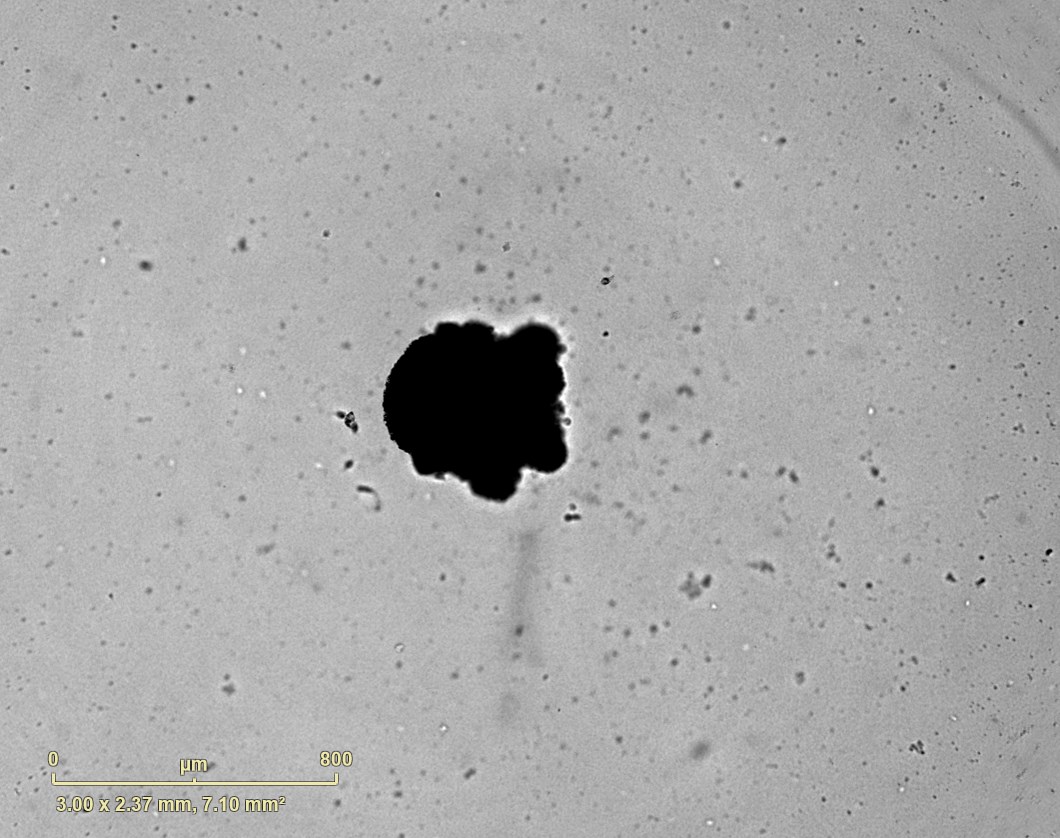

Supplement: Supplementary file 16 — Source data Fig. 3 [file 44318_2024_206_MOESM16_ESM.zip › Figure 3/3D/IMR5 whole spheroid - AU-15330.jpg]

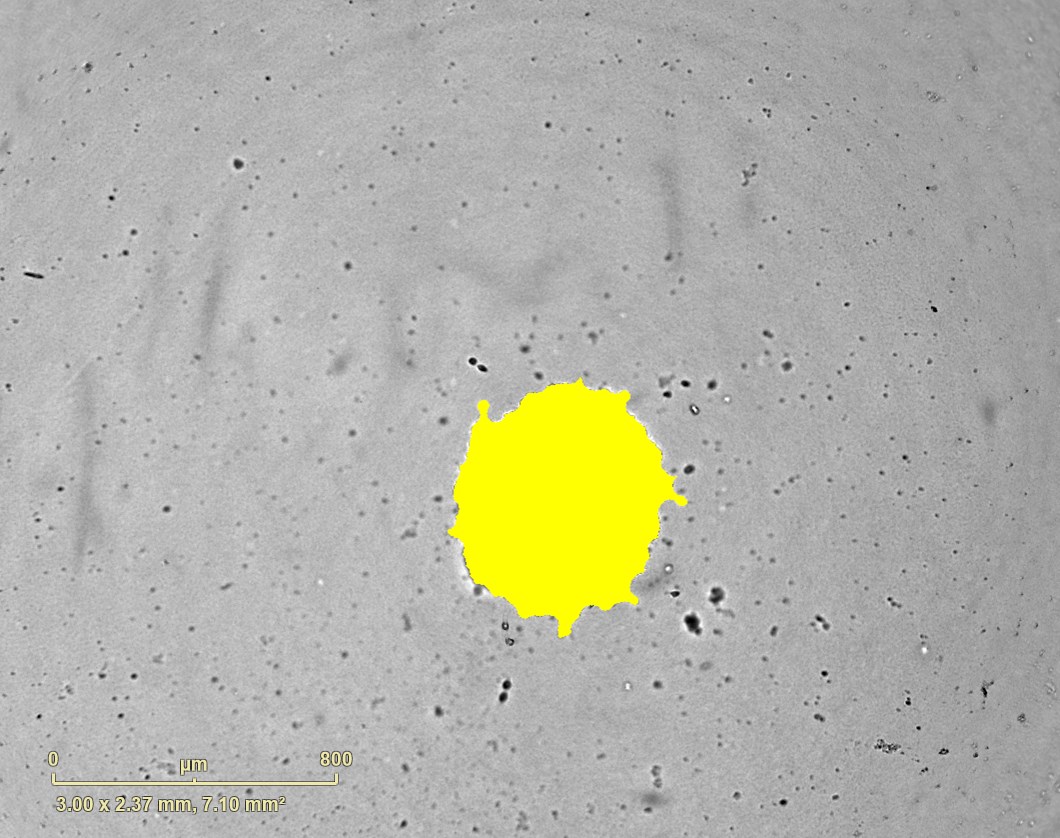

Supplement: Supplementary file 16 — Source data Fig. 3 [file 44318_2024_206_MOESM16_ESM.zip › Figure 3/3D/IMR5 invasion mask - ACBI1.jpg]

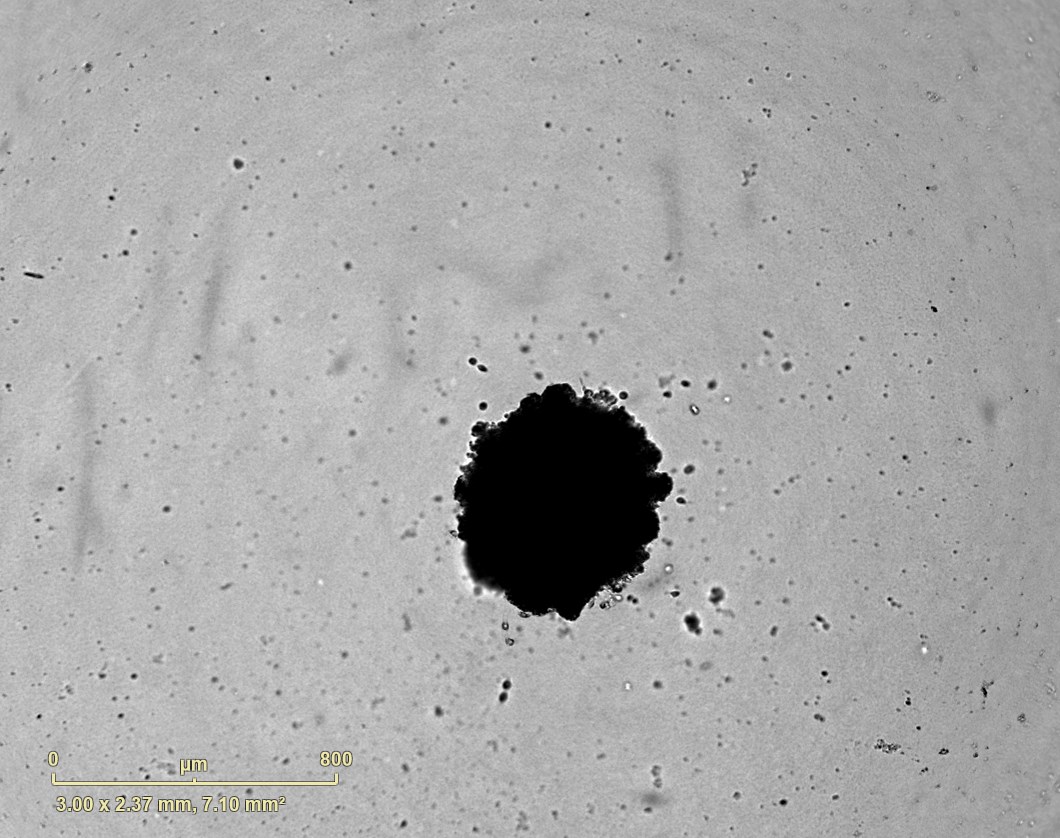

Supplement: Supplementary file 16 — Source data Fig. 3 [file 44318_2024_206_MOESM16_ESM.zip › Figure 3/3D/IMR5 whole spheroid - ACBI1.jpg]

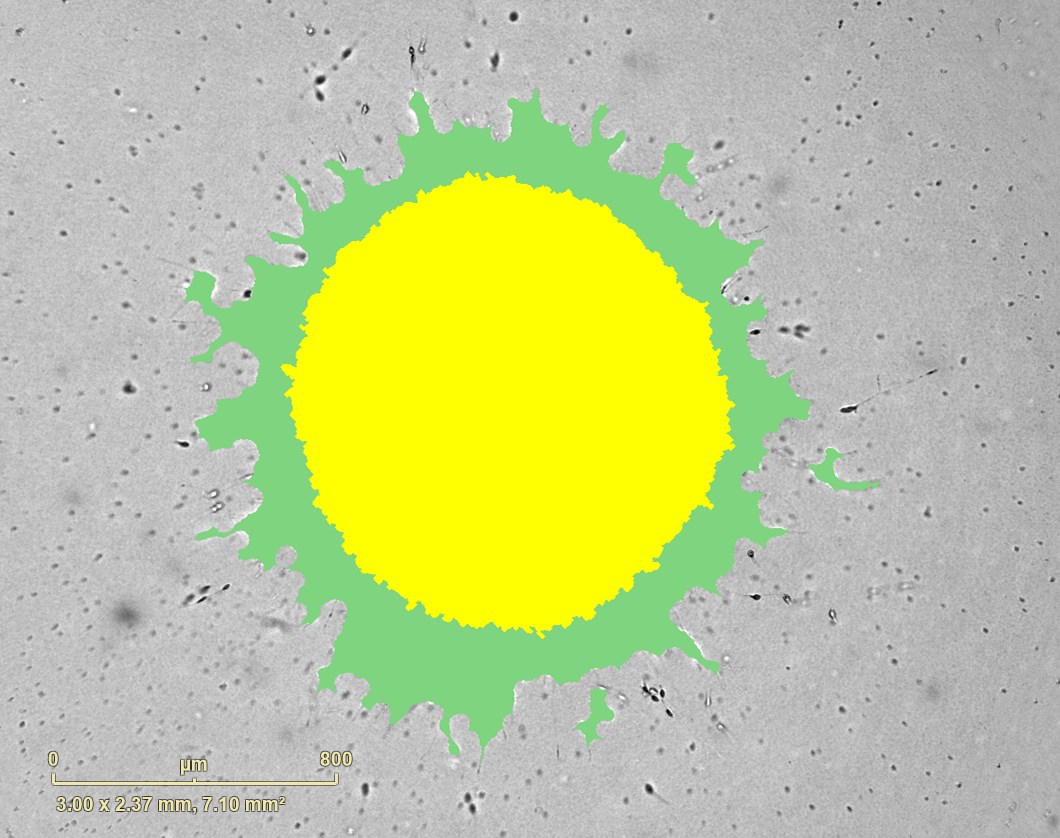

Supplement: Supplementary file 16 — Source data Fig. 3 [file 44318_2024_206_MOESM16_ESM.zip › Figure 3/3D/IMR5 invasion mask - DMSO.jpg]

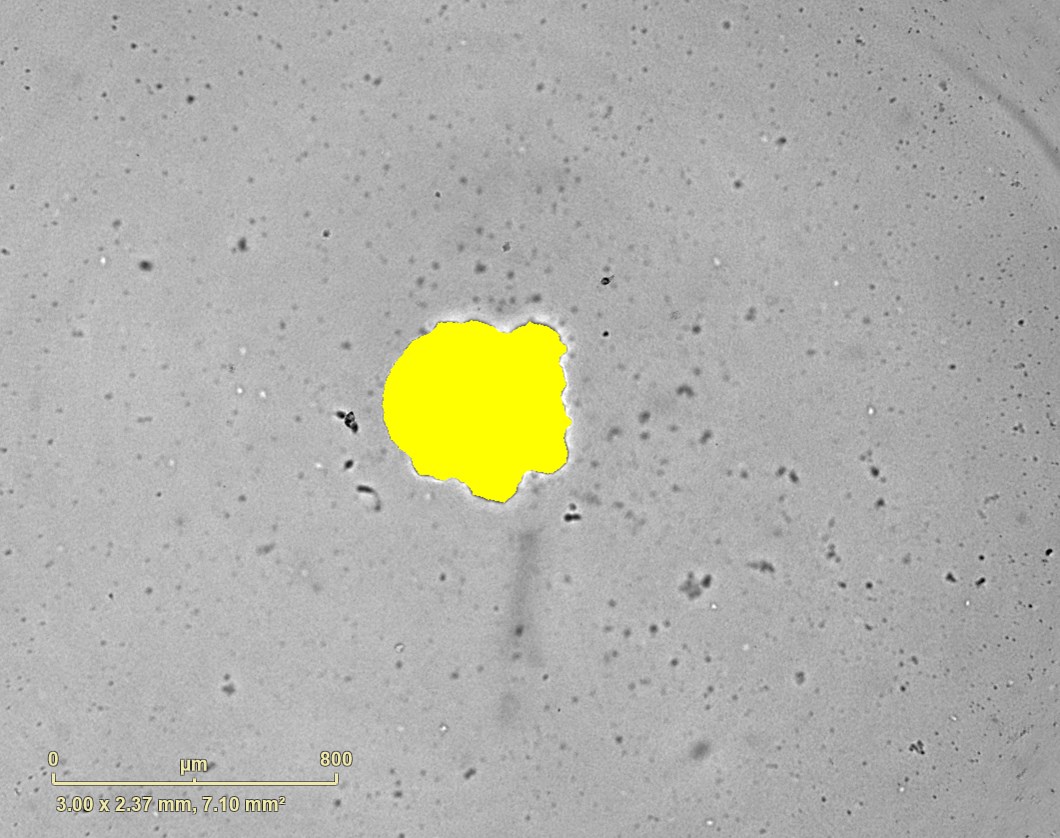

Supplement: Supplementary file 16 — Source data Fig. 3 [file 44318_2024_206_MOESM16_ESM.zip › Figure 3/3D/IMR5 invasion mask - AU-15330.jpg]

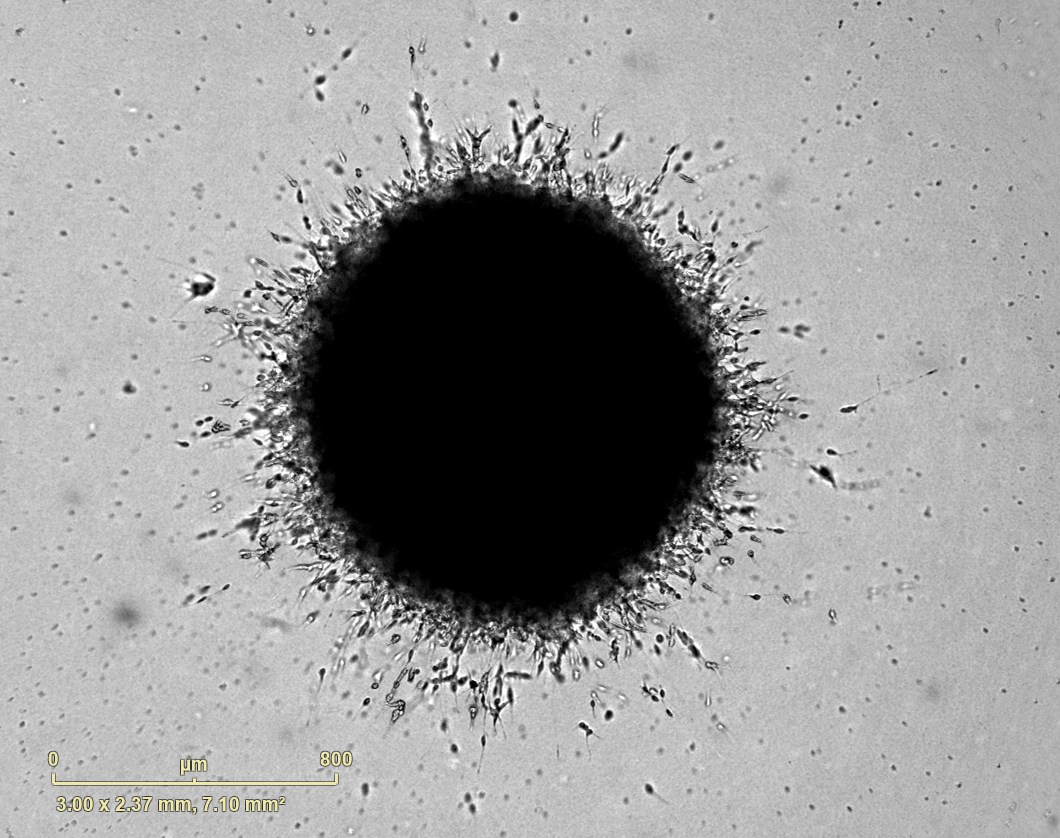

Supplement: Supplementary file 16 — Source data Fig. 3 [file 44318_2024_206_MOESM16_ESM.zip › Figure 3/3D/IMR5 whole spheroid - DMSO.jpg]

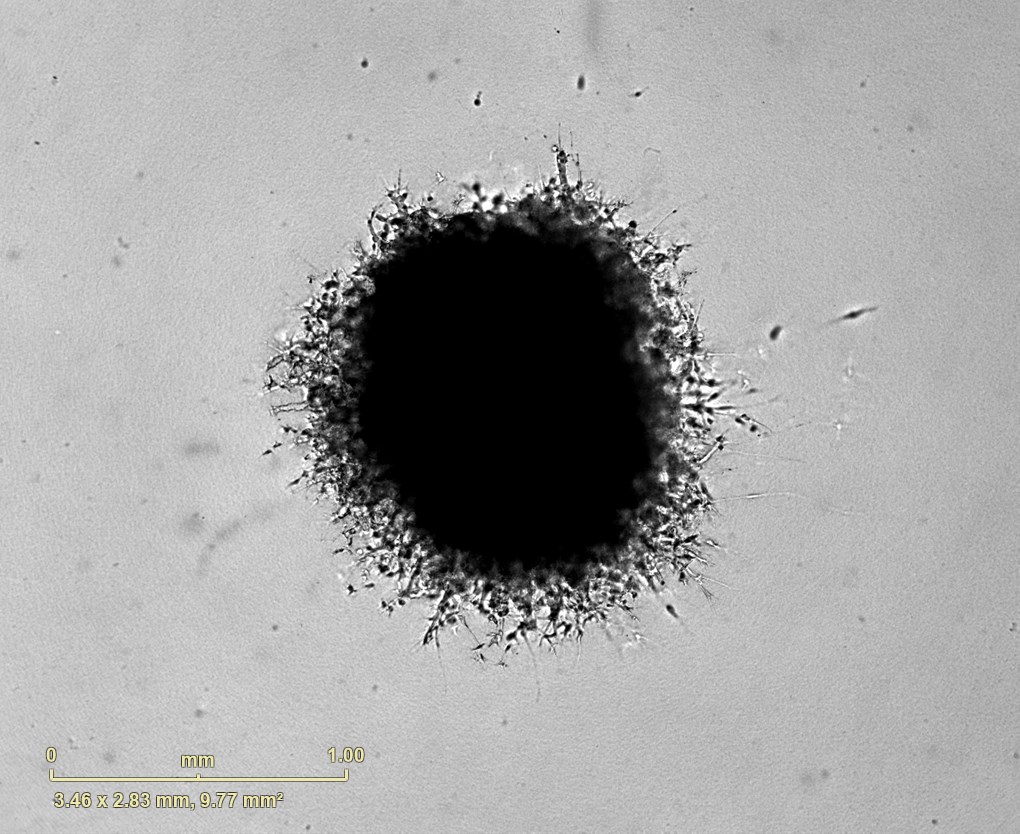

Supplement: Supplementary file 16 — Source data Fig. 3 [file 44318_2024_206_MOESM16_ESM.zip › Figure 3/3F/BE2C Spheroid - DMSO.jpg]

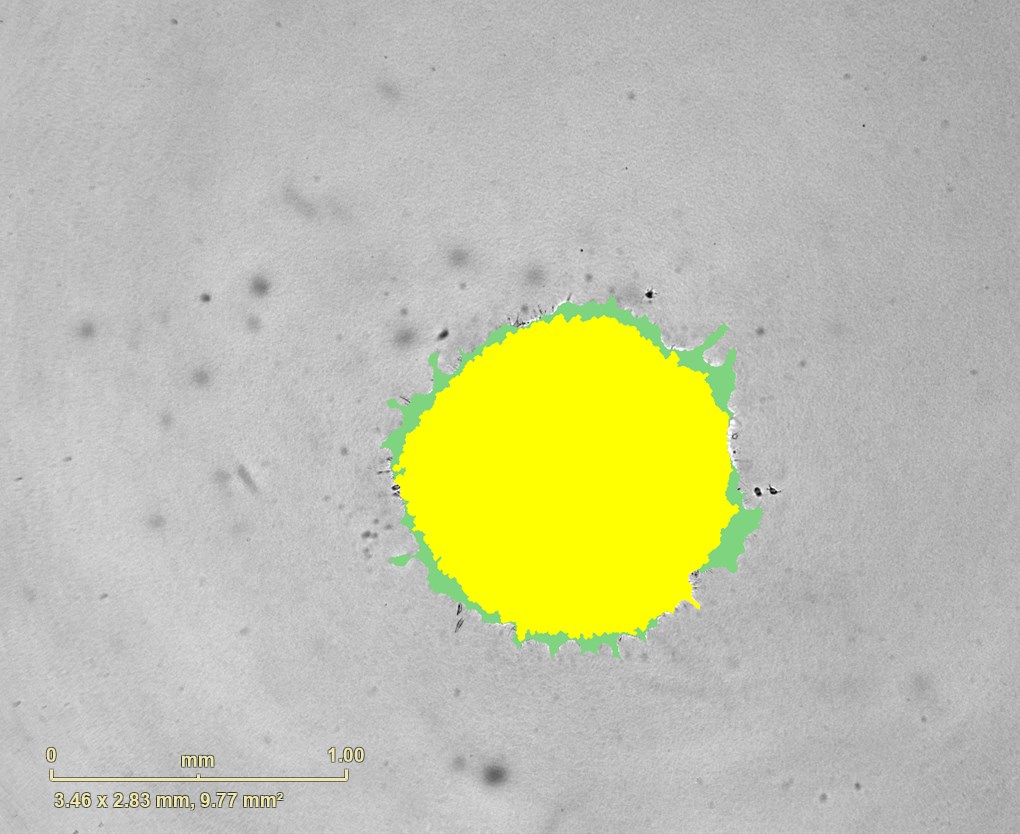

Supplement: Supplementary file 16 — Source data Fig. 3 [file 44318_2024_206_MOESM16_ESM.zip › Figure 3/3F/BE2C invasion mask - ACBI1.jpg]

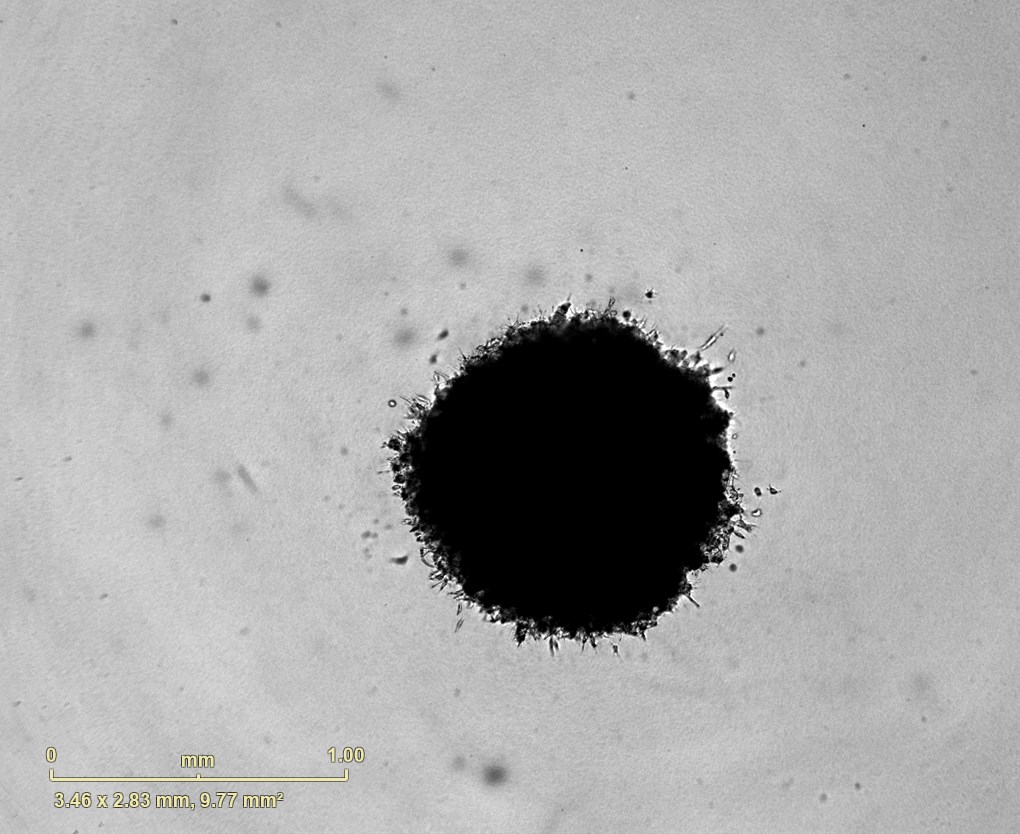

Supplement: Supplementary file 16 — Source data Fig. 3 [file 44318_2024_206_MOESM16_ESM.zip › Figure 3/3F/BE2C Spheroid - ACBI1.jpg]

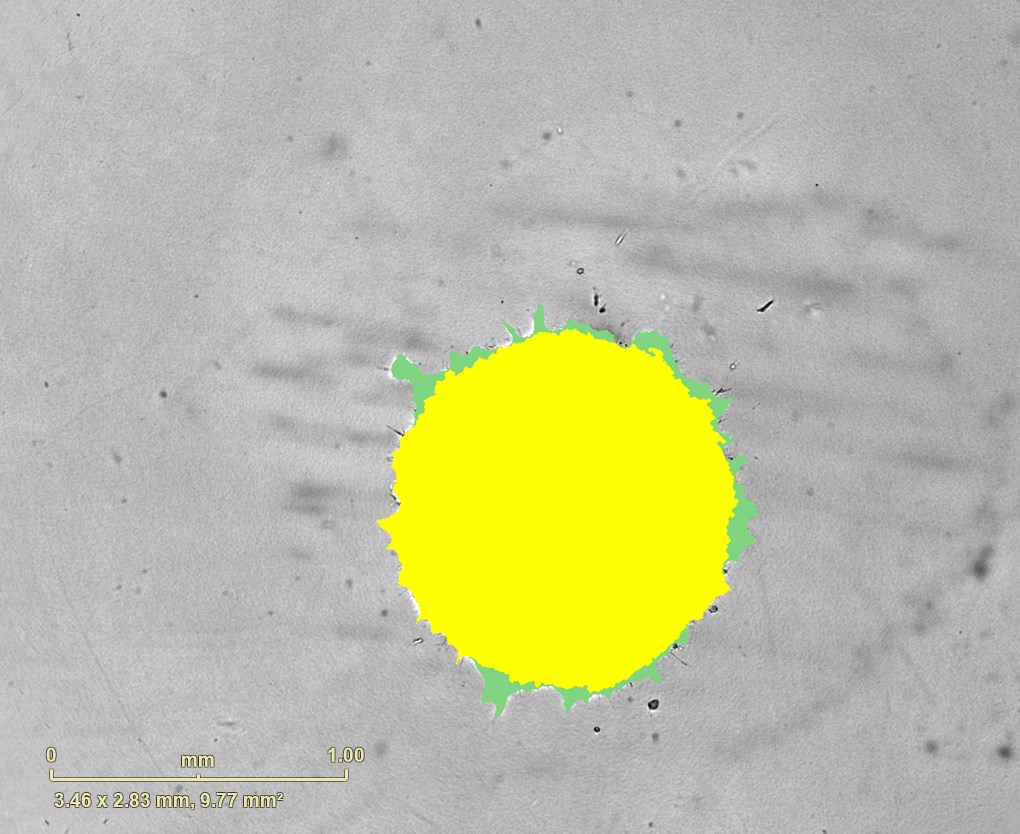

Supplement: Supplementary file 16 — Source data Fig. 3 [file 44318_2024_206_MOESM16_ESM.zip › Figure 3/3F/BE2C invasion mask - AU-15330.jpg]

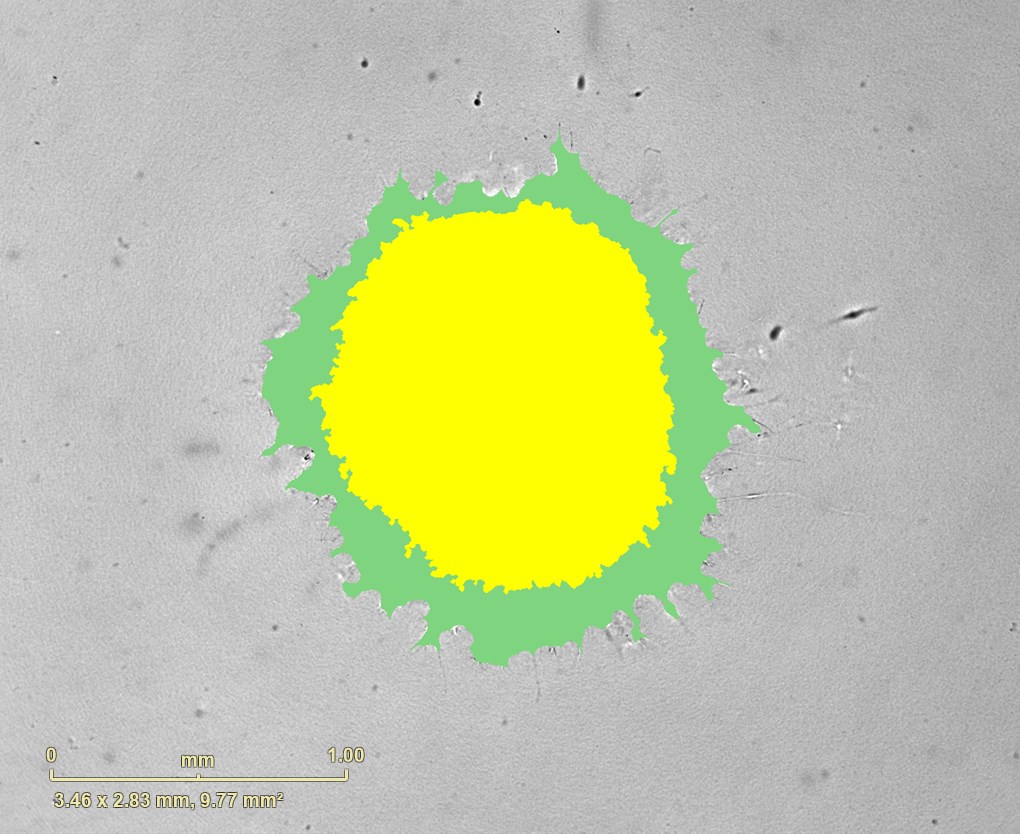

Supplement: Supplementary file 16 — Source data Fig. 3 [file 44318_2024_206_MOESM16_ESM.zip › Figure 3/3F/BE2C invasion mask - DMSO.jpg]

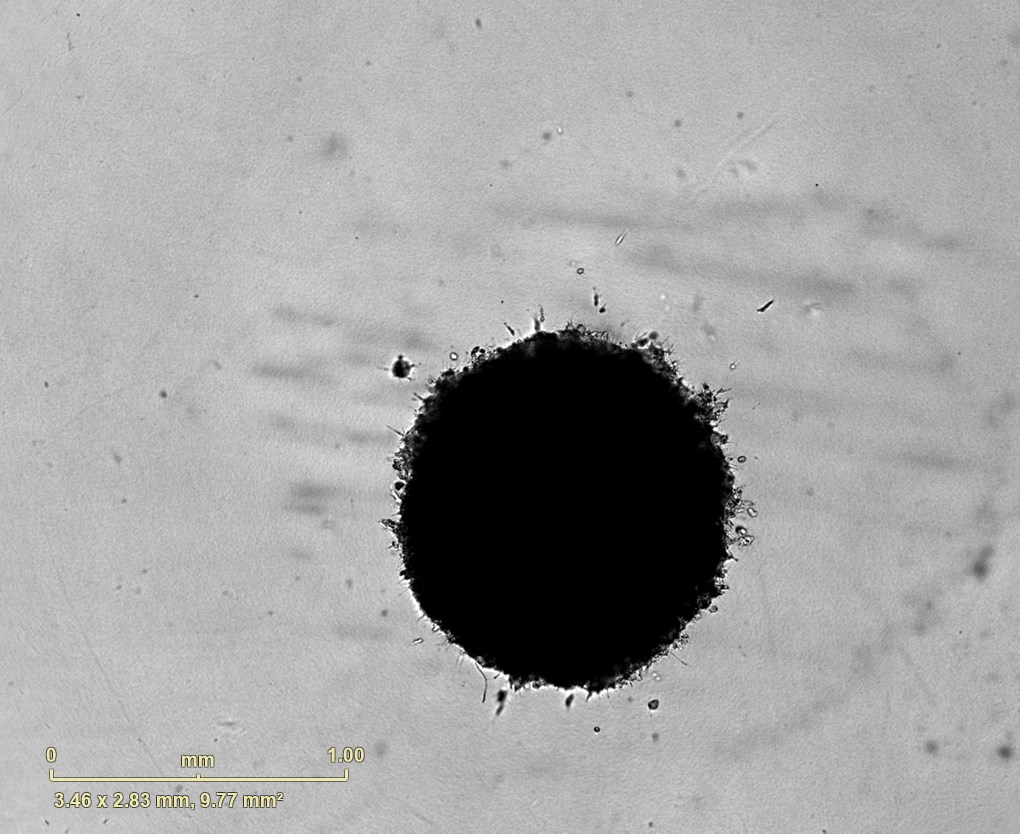

Supplement: Supplementary file 16 — Source data Fig. 3 [file 44318_2024_206_MOESM16_ESM.zip › Figure 3/3F/BE2C Spheroid - AU-15330.jpg]

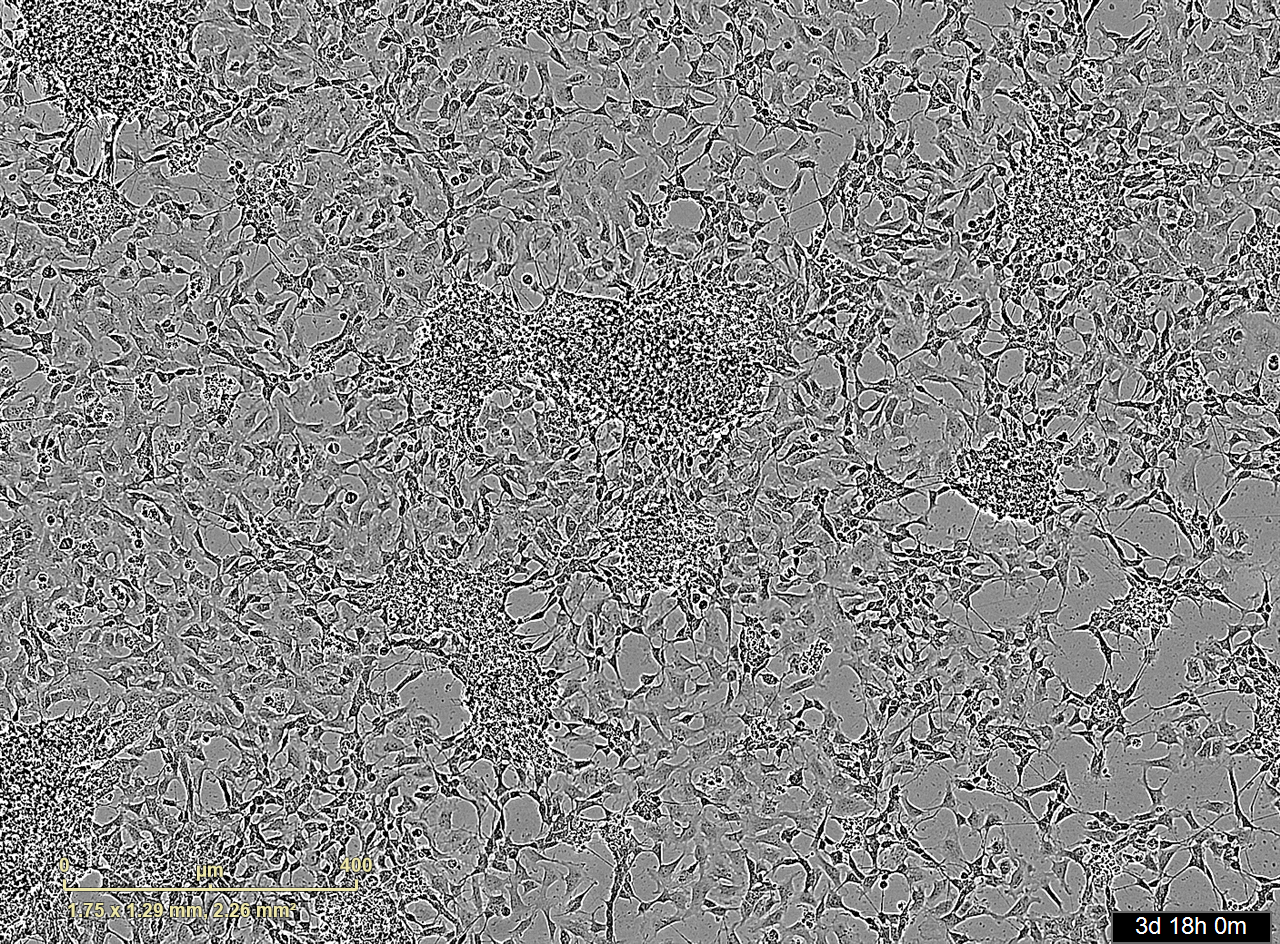

Supplement: Supplementary file 17 — Source data Fig. 5 [file 44318_2024_206_MOESM17_ESM.zip › Figure 5/5A/SJNBL012407_X1- FBS_DMSO.tif]

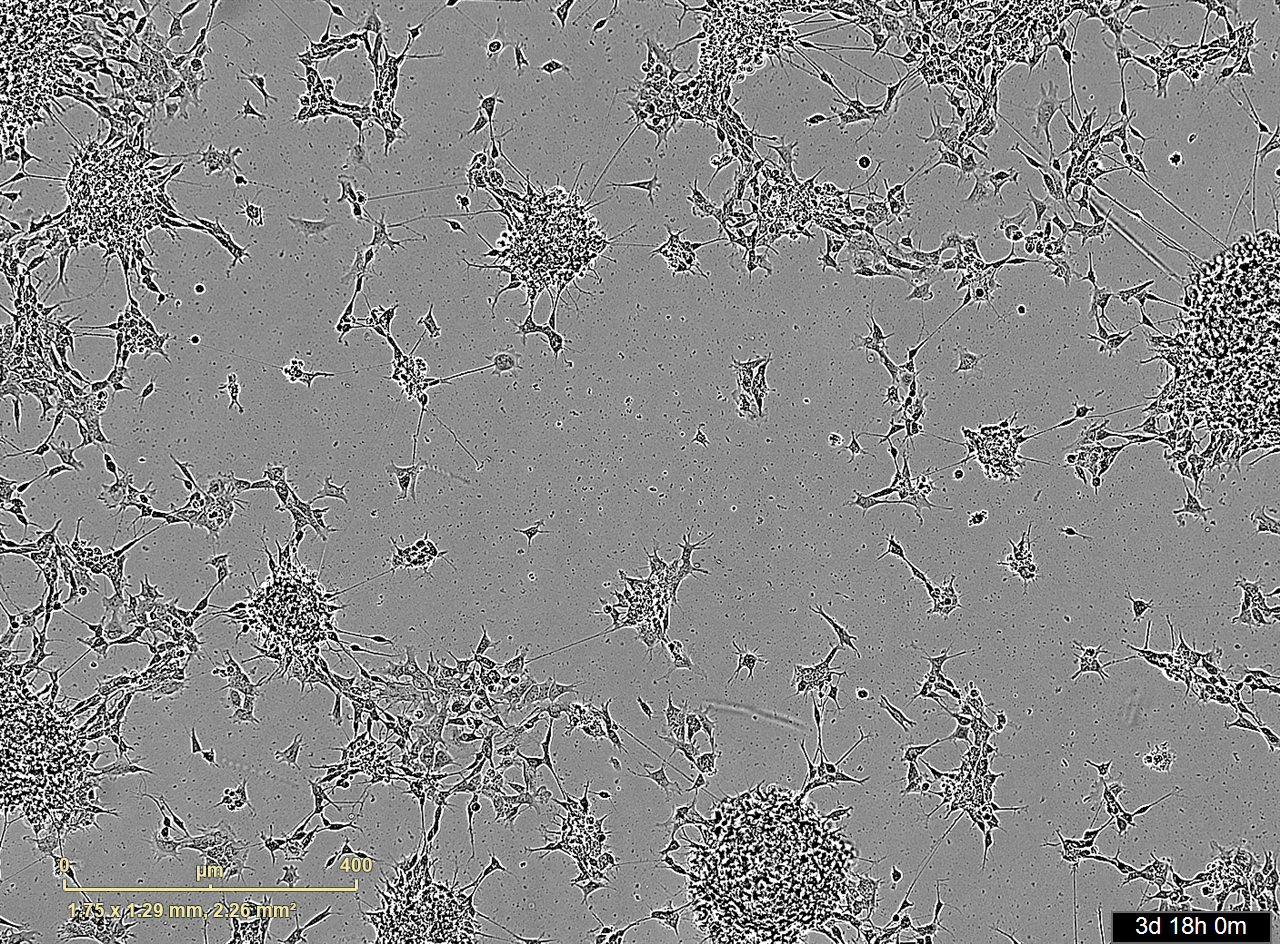

Supplement: Supplementary file 17 — Source data Fig. 5 [file 44318_2024_206_MOESM17_ESM.zip › Figure 5/5A/SJNBL012407_X1- FBS_ACBI1.tif]

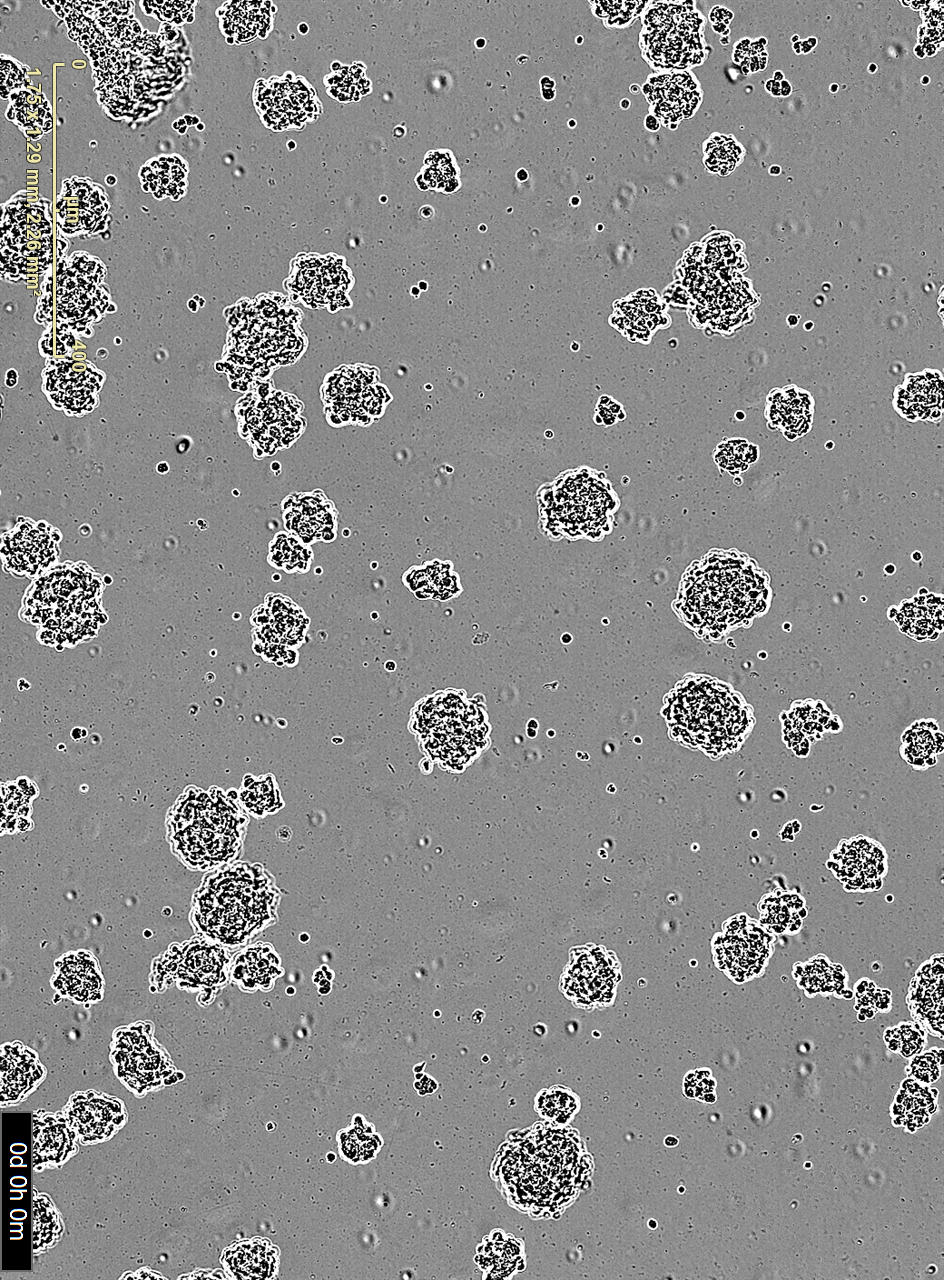

Supplement: Supplementary file 17 — Source data Fig. 5 [file 44318_2024_206_MOESM17_ESM.zip › Figure 5/5A/SJNBL012407_X1- SCM.tif]

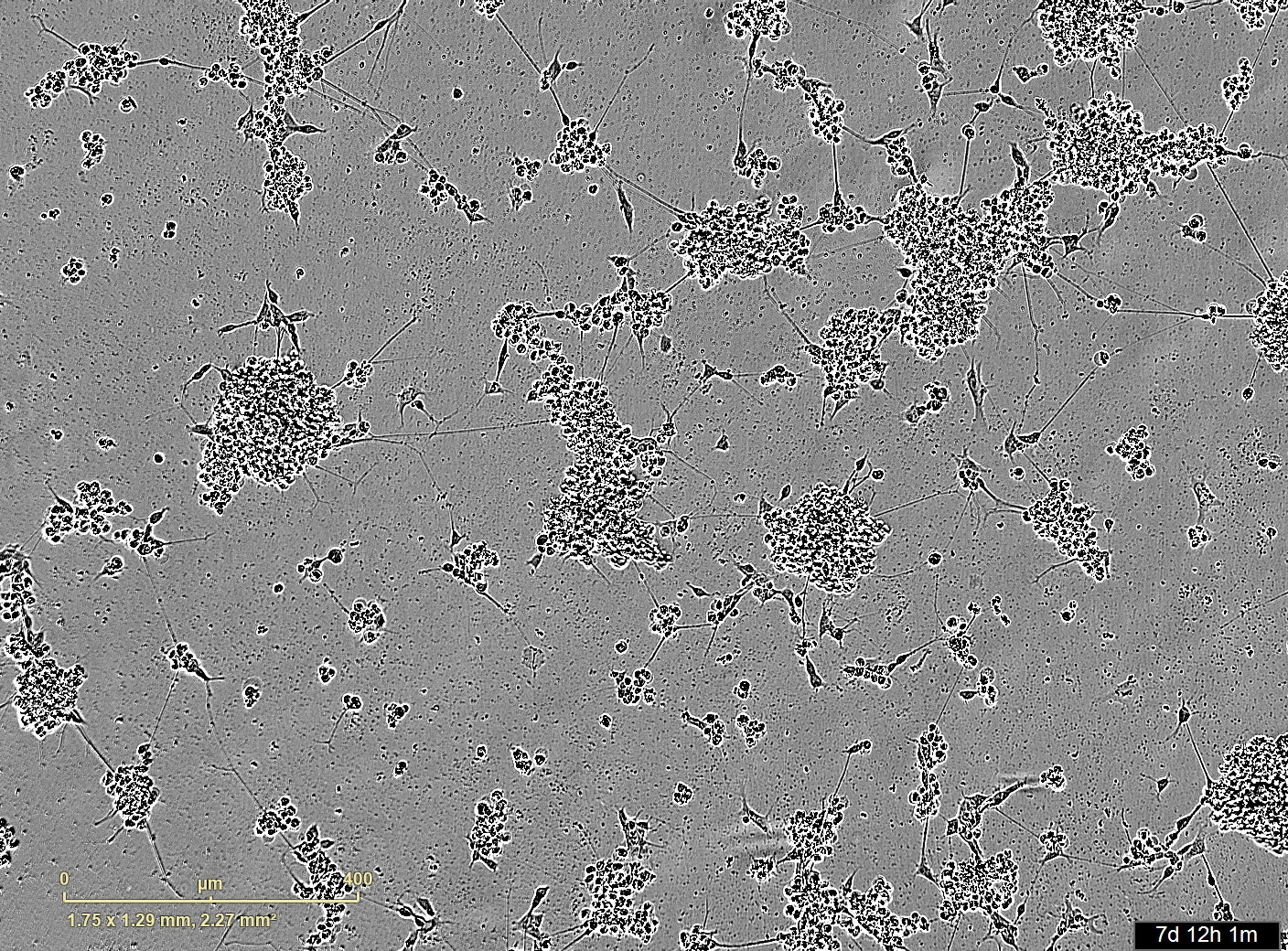

Supplement: Supplementary file 18 — Source data Fig. 6 [file 44318_2024_206_MOESM18_ESM.zip › Figure 6/6E/SJNBL012407_X1 image - A+E.tif]

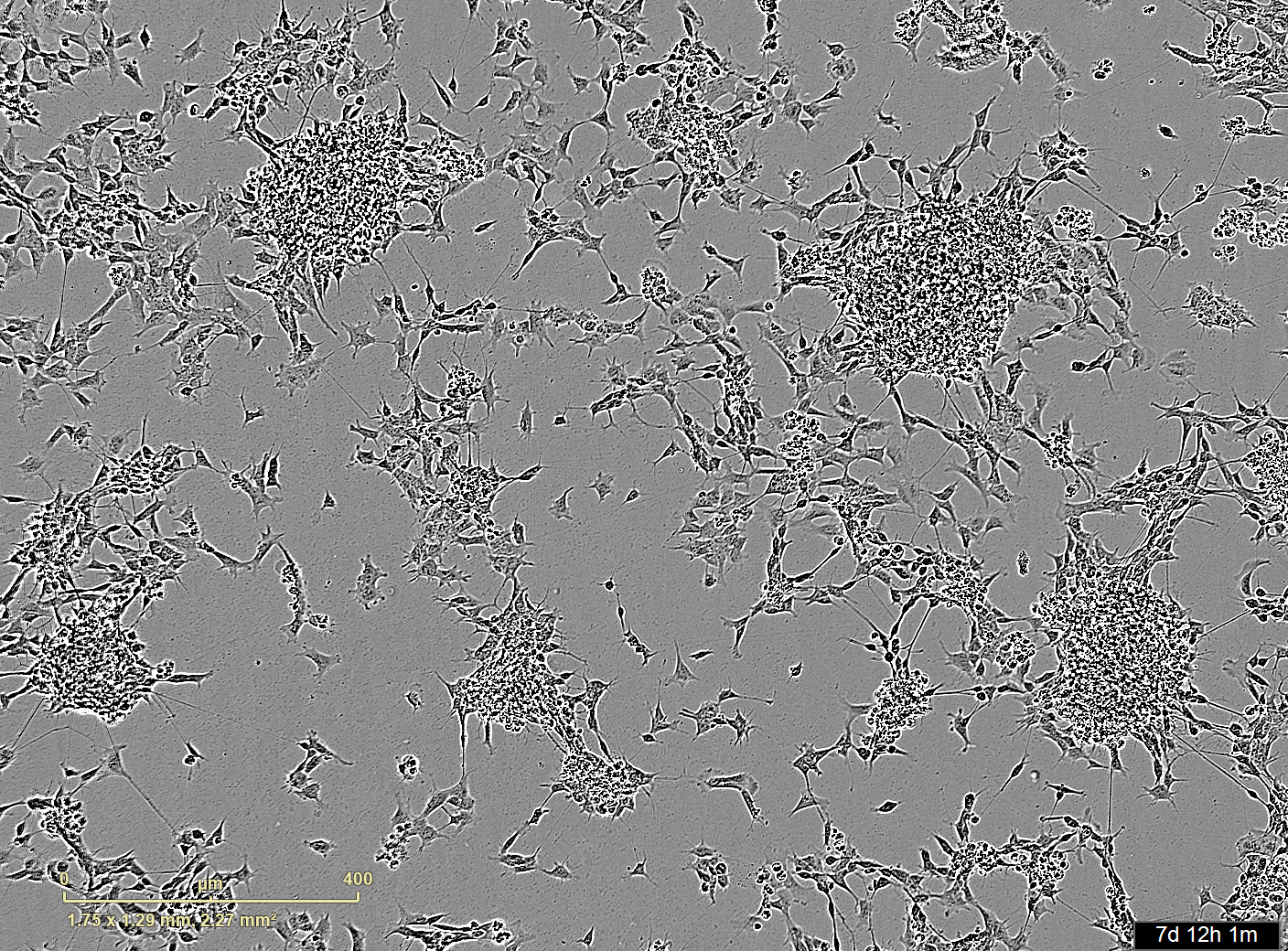

Supplement: Supplementary file 18 — Source data Fig. 6 [file 44318_2024_206_MOESM18_ESM.zip › Figure 6/6E/SJNBL012407_X1 image - ACBI1.tif]

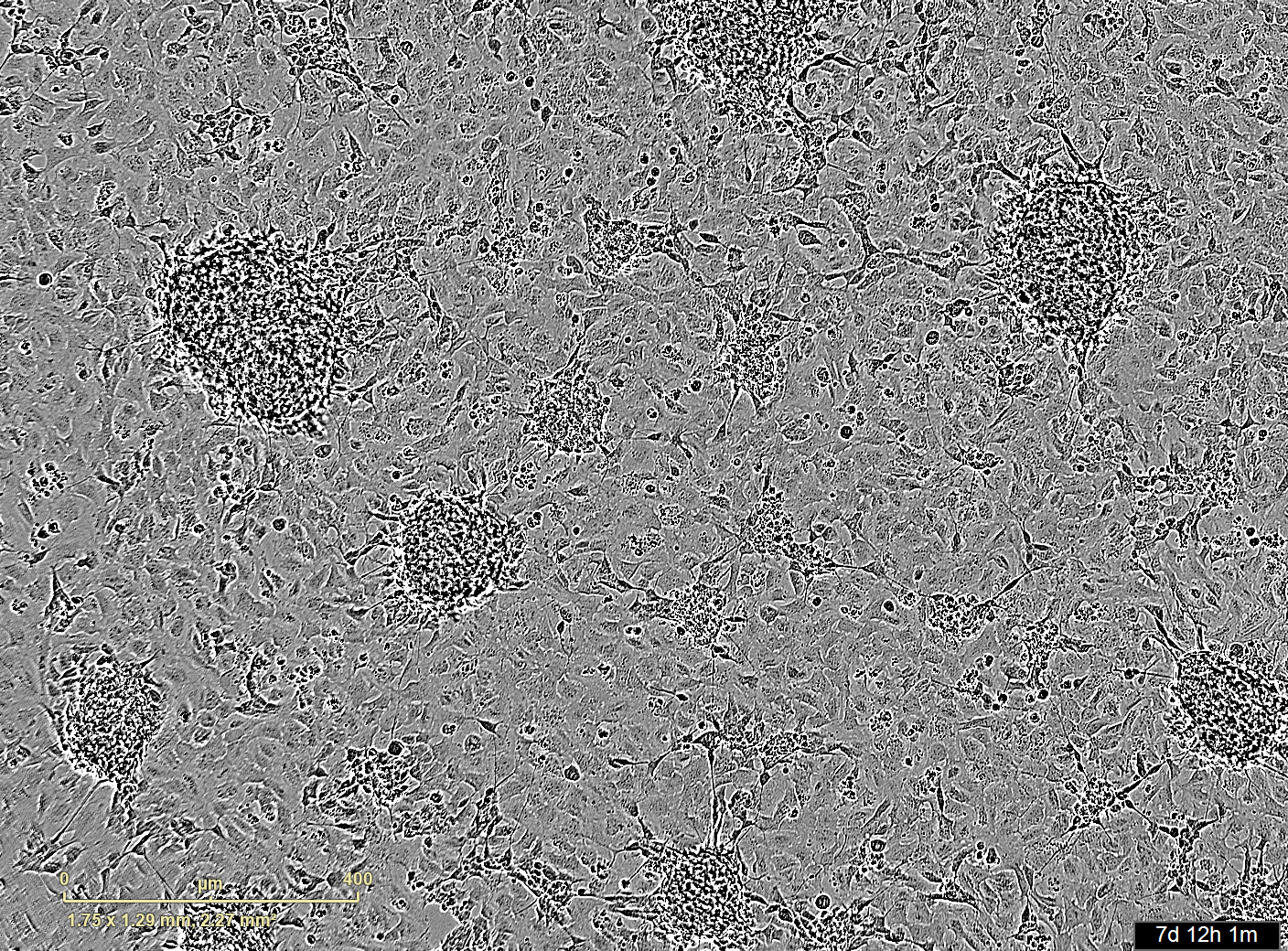

Supplement: Supplementary file 18 — Source data Fig. 6 [file 44318_2024_206_MOESM18_ESM.zip › Figure 6/6E/SJNBL012407_X1 image - DMSO.tif]

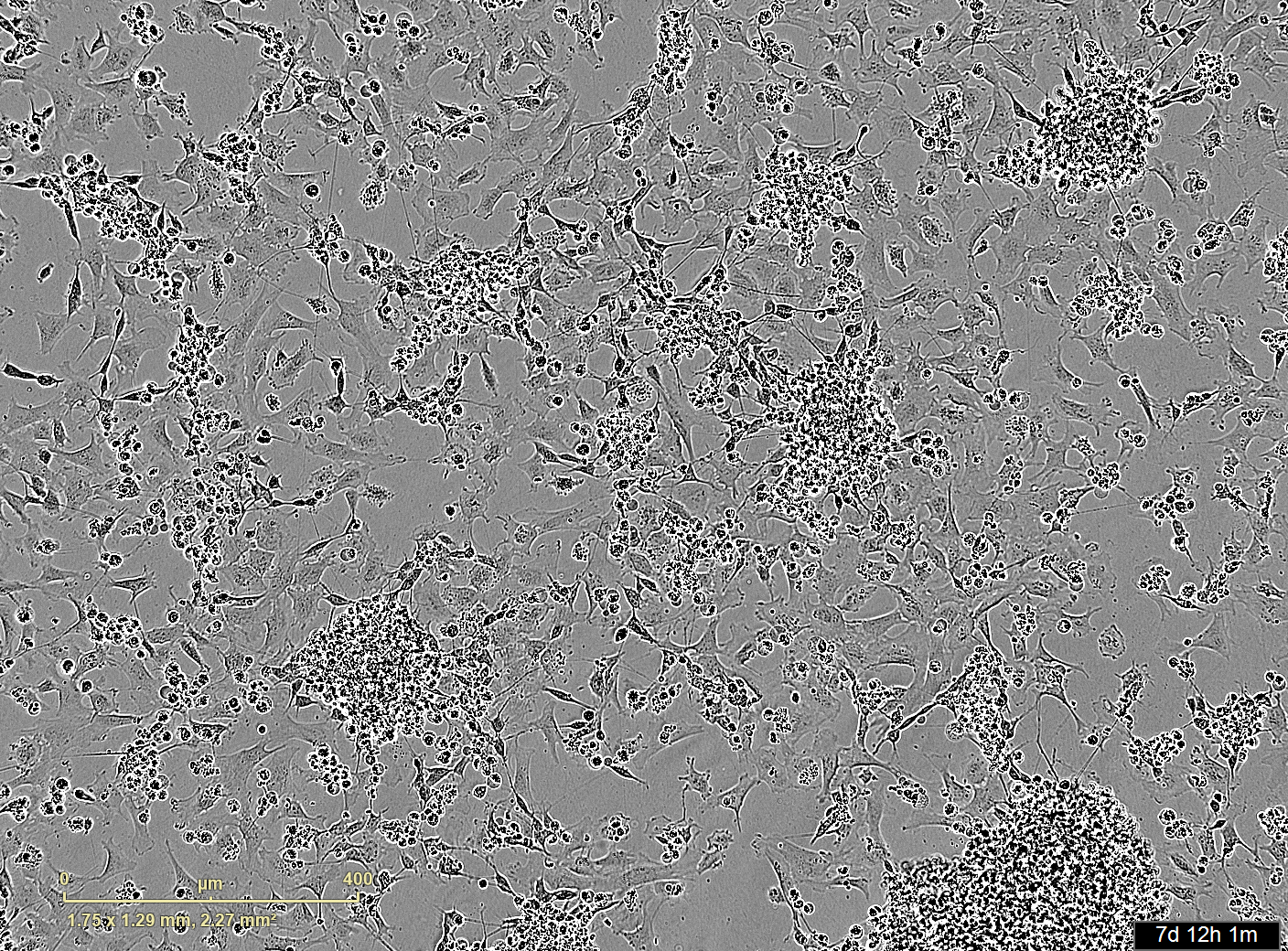

Supplement: Supplementary file 18 — Source data Fig. 6 [file 44318_2024_206_MOESM18_ESM.zip › Figure 6/6E/SJNBL012407_X1 image - Etoposide.tif]
